# Organogenetic transcriptomes of the *Drosophila* embryo at single cell resolution

**DOI:** 10.1242/dev.202097

**Published:** 2024-01-19

**Authors:** Da Peng, Dorian Jackson, Bianca Palicha, Eric Kernfeld, Nathaniel Laughner, Ashleigh Shoemaker, Susan E. Celniker, Rajprasad Loganathan, Patrick Cahan, Deborah J. Andrew

**Affiliations:** ^1^ Department of Biomedical Engineering, Johns Hopkins University School of Medicine, Baltimore, MD 21205, USA; ^2^ Department of Cell Biology, Johns Hopkins University School of Medicine, Baltimore, MD 21205, USA; ^3^ Biological Systems and Engineering Division, Lawrence Berkeley National Laboratory, Berkeley, CA 94720, USA; ^4^ Department of Biological Sciences, Wichita State University, Wichita, KS 67260, USA

**Keywords:** Germ cells, Organogenesis, Plasmatocytes, Proteolysis, Salivary gland, Single cell RNA sequencing (scRNA-seq), Trachea, Matrisome

## Abstract

To gain insight into the transcription programs activated during the formation of *Drosophila* larval structures, we carried out single cell RNA sequencing during two periods of *Drosophila* embryogenesis: stages 10-12, when most organs are first specified and initiate morphological and physiological specialization; and stages 13-16, when organs achieve their final mature architectures and begin to function. Our data confirm previous findings with regards to functional specialization of some organs – the salivary gland and trachea – and clarify the embryonic functions of another – the plasmatocytes. We also identify two early developmental trajectories in germ cells and uncover a potential role for proteolysis during germline stem cell specialization. We identify the likely cell type of origin for key components of the *Drosophila* matrisome and several commonly used *Drosophila* embryonic cell culture lines. Finally, we compare our findings with other recent related studies and with other modalities for identifying tissue-specific gene expression patterns. These data provide a useful community resource for identifying many new players in tissue-specific morphogenesis and functional specialization of developing organs.

## Teaser

**Summary:** Single cell sequencing during key stages of *Drosophila* organogenesis reveals expected and previously unreported developmental trajectories and functions of specialized cell types, and uncovers a potential role for proteolysis in germ cell specialization.

## INTRODUCTION

Organogenesis transforms the genetically and morphologically homogeneous early embryonic cell population into heterogeneous entities, i.e. organs, during development. The crucial cellular transformations required for organogenesis occur primarily via multipotency, gastrulation, specification, diversification, morphogenesis and terminal differentiation (Edlund, 2002; Pispa and Thesleff, 2003; Si-Tayeb et al., 2010; Sivakumar and Kurpios, 2018; Thesleff et al., 1995; Zorn and Wells, 2009). Cells, both individually and collectively, act in concert with the extracellular matrix (ECM) to self-assemble organs and organ systems (Devine et al., 2005; Loganathan et al., 2016c; Serna-Morales et al., 2023; Walma and Yamada, 2020). Shaped by gene regulatory networks and signaling events, transcriptomes drive the transformation of cells into organs (Bhattaram et al., 2010; Castelli-Gair Hombria et al., 2016; Dassaye et al., 2016; Huilgol et al., 2019; Selleri et al., 2019).

An understanding of the molecular underpinnings of organogenesis has gradually emerged over the past 50 years (Castelli-Gair Hombria and Bovolenta, 2016). Historically, discoveries were mutation driven. Naturally occurring mutations and genetic screens for zygotic and maternal-effect mutations that disrupt normal development revealed genes that contribute to global embryonic patterning, morphogenetic processes and organ development (Amsterdam et al., 1999; Boulin and Bessereau, 2007; De Stasio and Dorman, 2001; Hrabe de Angelis et al., 2000; Johnsen and Baillie, 1988; Jurgens et al., 1984; Luschnig et al., 2004; Mitchell et al., 2001; Mullins et al., 1994; Nolan et al., 2000; Nüsslein-Volhard and Wieschaus, 1980; Nüsslein-Volhard et al., 1984; Skarnes et al., 1995; Soewarto et al., 2000; Wansleeben et al., 2011; Wieschaus et al., 1984). These genes were eventually cloned and characterized to reveal information about the corresponding proteins or regulatory RNAs, and about when and where the molecules were expressed and localized (Wieschaus and Nüsslein-Volhard, 2016). Collectively, mutation-driven studies have revealed that developmental genes are not only conserved across animalia (or even more broadly) (Heffer and Pick, 2013) (https://www.sdbonline.org/sites/FLY/aimain/aadevinx.htm) but are also deployed multiple times during development (Calcott, 2014; Monteiro, 2012; Verd et al., 2019). Nonetheless, mutations in some organogenesis-related genes are difficult to detect: mutations in functionally redundant genes, mutations in genes with subtle phenotypes and mutations in genes whose contributions to organogenesis varies temporally (Calcott, 2014; Kafri et al., 2006; Wagner, 1996). Therefore, it is likely that many components of organogenesis remain undiscovered.

Complementing forward genetic approaches, genome-wide approaches, such as microarrays and next-generation sequencing, have been widely used to characterize gene expression patterns across tissues and developmental stages. Consortia have arisen whose goal is the functional annotation of genomic elements across different model organisms and a determination of which genes are expressed in different tissues dissected from various developmental stages (Brown et al., 2014; Gerstein et al., 2010; Graveley et al., 2011). For *Drosophila* embryos, which are too small to isolate individual body parts for gene expression analyses, the Berkeley *Drosophila* Genome Project (BDGP) has used a different approach (Weiszmann et al., 2009). Using optimal cDNA probes, they have carried out whole-mount *in situ* hybridization for 8593 genes (∼63% of known *Drosophila* genes) and have made these data available through a searchable public database with over 140,000 digital images organized by gene and developmental stage. This resource has been the foundation for discovering the regulatory networks that distinguish different organs and for uncovering the functions of a multitude of developmental genes. Nonetheless, even this endeavor remains incomplete and other approaches to discovering the array of transcripts expressed in organs at crucial developmental periods are necessary.

Single cell RNA sequencing (scRNA-seq) has obviated the need to prospectively isolate pure cell populations before determining cell type-specific expression profiles (Macosko et al., 2015; Tang et al., 2009). scRNA-seq has been widely applied to explore embryonic transcriptional states in several model organisms, including *C. elegans* (Cao et al., 2017), *D. melanogaster* (Calderon et al., 2022; Karaiskos et al., 2017; Sakaguchi et al., 2023; Seroka et al., 2022), *X. tropicalis* (Briggs et al., 2018), *D. rerio* (Farrell et al., 2018) and *M. musculus* (Cao et al., 2019; Pijuan-Sala et al., 2019). The *Drosophila* embryo stands out among these model systems as a pre-eminent case study for demonstrating the utility of these studies. For example, scRNA-seq analysis from pre-organogenetic *Drosophila* embryonic cells uncovered a previously unappreciated role for Hippo signaling in breaking the synchronicity of cell cycle re-entry (Karaiskos et al., 2017). A recent study demonstrated the power of scRNA-seq to profile key cell-state transitions required in a single neuroblast lineage during *Drosophila* embryonic neurogenesis (Seroka et al., 2022). A temporally dense scRNA-seq dataset of *Drosophila* embryogenesis led to development of a neural network-based model to predict developmental nuclear age (Calderon et al., 2022). Although these previous investigations capture the single cell transcriptome for early embryogenesis (pre-gastrulation) (Karaiskos et al., 2017), gastrulation (Sakaguchi et al., 2023), neurogenesis (Seroka et al., 2022) and enhancer accessibility/use for key developmental transitions (Calderon et al., 2022), the transcriptomic analyses associated with organogenesis, i.e. cell type specification, growth, migration, terminal differentiation and functional priming, are lacking. Here, we obtain and analyze single cell transcriptomes from early (stage 10-12) and late (stage 13-16) organogenetic stages of the *Drosophila* embryos to focus on the specification and morphogenetic processes required for the assembly and terminal differentiation of three organs: (1) the salivary gland (SG), (2) the trachea and (3) the germline.

Our analysis of the SG demonstrates the sequential deployment of biological processes and the associated transcriptomes in the assembly and functional priming of an exemplary model secretory organ. Our data from the trachea offer insight into the varied transcriptome profiles of cells involved in the assembly of anastomosing tubes and concomitant regulation of tubule growth during epithelial branching morphogenesis. Our investigation of the embryonic germline identifies two early trajectories and suggests that protein catabolism is enriched in presumptive germline stem cells. Further analysis of our scRNA-seq data identifies novel tissue-specific sources of the *Drosophila* matrisome in addition to confirming known sources. To demonstrate the extended utility of our single cell organogenetic transcriptomes, we use these data to track the embryonic origins of some commonly used *Drosophila* tissue culture cells. Finally, we compare our data with the previously published *Drosophila* single cell transcriptomes using an extensive array of tools, finding both similarities and key differences across the available embryonic transcriptome datasets.

## RESULTS

### Obtaining single cell transcriptome data during organogenesis

**Fig. 1.**
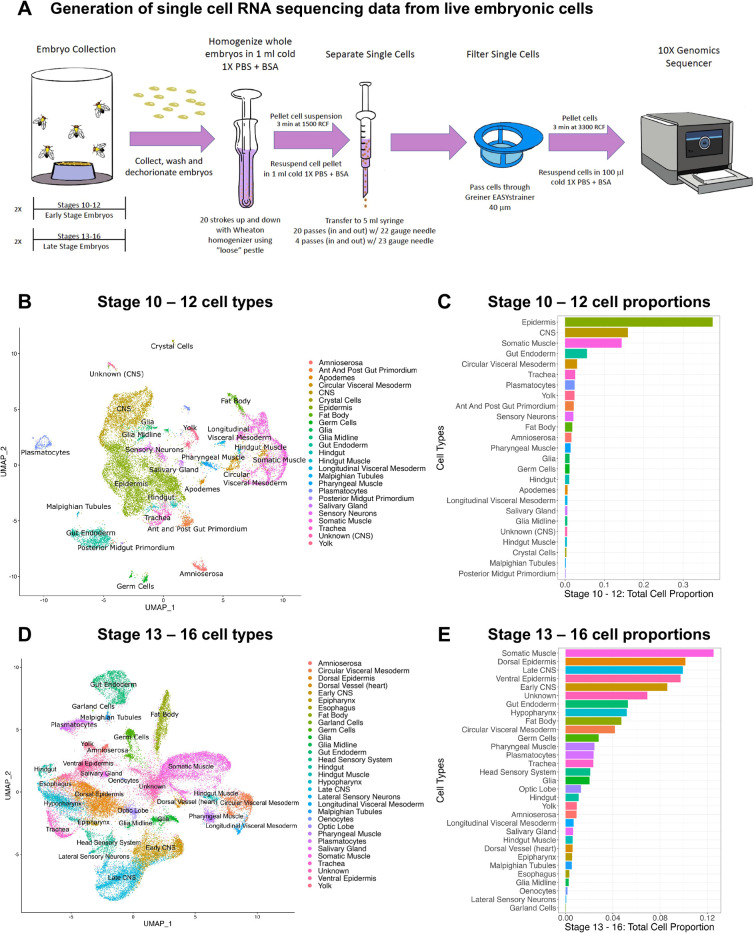
**Single cell isolation and cell type annotations of stage 10-12 and stage 13-16 embryonic cells.** (A) Schematic of embryo collection, single cell isolation and sequencing. (B) Scatter plot showing the UMAP embedding colored by cell types from stage 10-12 embryonic cells. (C) Bar plot showing the cell type proportion of stage 10-12 cells. (D) Scatter plot showing the UMAP embedding colored by cell types from stage 13-16 embryonic cells. (E) Bar plot showing the cell type proportion of stage 13-16 cells.

Single cells were isolated from whole dechorionated wild-type embryos (Fig. 1A). After assessment of overall cell viability (>90%) and concentration, these live cell samples were immediately processed for 10X Genomics scRNAseq. Two biological replicates spanning early (stages 10-12) and late (stages 13-16) organogenesis were obtained; see collection details in the Materials and Methods (Fig. 1A, Table S1). After standard quality control to remove poor-quality reads and likely doublets, 20,585 cells (median UMI count 1065) for stages 10-12 and 42,717 cells (median UMI count 1155) for stages 13-16 were recovered. Standard batch correction of the replicates was carried out using Harmony (Korsunsky et al., 2019a) (Fig. S1A,C). The two replicates from each timepoint were then combined to generate both early (Fig. 1B) and late (Fig. 1D) Uniform Manifold Approximation Projections (UMAPs) using Seurat.

The differentially expressed genes for each cluster were identified (Table S2), and the selected marker genes from the top 50 most differentially expressed genes were cross-referenced with the BDGP *in situ* expression data (Tomancak et al., 2002) (Table S3) to assign 25 distinct cell types (Fig. 1B) from 35 early single-cell clusters (Fig. S1B,E) and 31 distinct cell types (Fig. 1D) from 36 late single-cell clusters (Fig. S1D,F). Although many more gene expression profiles were typically used to assign cell types, key marker genes for each tissue were identified (Table S3).

Twenty cell types were found from both the early and late organogenetic transcriptome signatures: (1) amnioserosa, (2) circular visceral mesoderm, (3) central nervous system (CNS), (4) epidermis, (5) fat body, (6) germ cells, (7) glia, (8) midline glia, (9) gut endoderm, (10) hindgut, (11) hindgut muscle, (12) longitudinal visceral mesoderm, (13) Malpighian tubule, (14) pharyngeal muscle, (15) plasmatocytes, (16) salivary gland, (17) sensory neurons, (18) somatic muscle, (19) trachea and (20) yolk. Unknown cell types – cells that could not be assigned based on the range of expression patterns observed for the most enriched genes in the clusters – were present at both stages, with a smaller number of unknown cells in the early data.

Additional cell types were identified in the early stages only: anterior/posterior gut primordium, apodemes, crystal cells and posterior midgut primordium. Other cell types were identified in the late stages: dorsal vessel (heart), epipharynx, esophagus, garland cells, head sensory system, hypopharynx and oenocytes.

Examination of relative cell proportions revealed that cells of the epidermis, CNS and muscle are the major contributors to both stage 10-12 and stage 13-16 embryos (Fig. 1C,E), validating previous findings (Campos-Ortega and Hartenstein, 2013). We did not, however, recover cells in exact proportion to their representation in the embryo. For example, the relative proportion of muscle cells is expected to decrease in the late samples as muscle cell fusion occurs then and would decrease the total muscle cell number (Bothe and Baylies, 2016). Nonetheless, muscle cells made up ∼20% of cells at both stages (combining cells with ‘mesoderm’ or ‘muscle’ in the name). Likewise, SG cells were under-represented at 0.5% and 0.6% at early and late stages, respectively, when we would expect ∼3% to be SG during both stages. In addition, although we did capture clusters corresponding to rare cell types at both stages, not all cell types were captured as distinct clusters. Some of the cell types that were not represented, such as the somatic gonadal precursors, were found embedded in a cluster of other mesodermal cells. In contrast, plasmatocytes and germ cells have two separated clusters in the early collections.

To ascertain the utility of the data and to confirm and/or gain insight into the transcriptome dynamics underlying organ formation, we chose three organs – the salivary gland, trachea and germline (all of which were identified in both staged datasets) – for more in-depth analysis.

### Embryonic SG single cell transcriptome: an organogenetic blueprint for assembling a tissue that is specialized for secretion

**Fig. 2.**
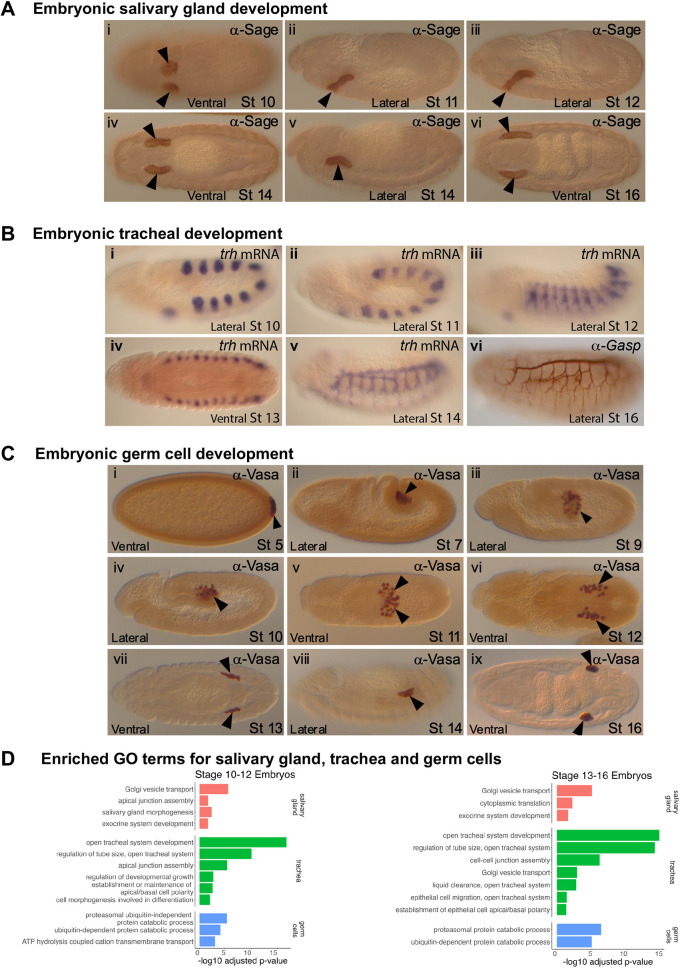
**Embryonic development and gene set enrichment analysis of salivary gland, tracheal and germ cells.** (A-C) Protein and mRNA staining of (A) Sage (protein), (B) *trh* (mRNA) and 2A12 (Gasp) (protein), and (C) Vasa (protein) to demonstrate the formation of the salivary gland (SG; A), trachea (B) and germline cells (GCs; C) across different developmental stages in *Drosophila* embryos. Arrowheads indicate the secretory cells of the salivary gland in A and the germ cells in C. (D) Selected gene sets enriched in SG, trachea and GCs relative to the rest of the embryonic cells during stage 10-12 (on the left) and stage 13-16 (on the right). For the full enrichment analysis results, see Table S4.

Salivary glands (SGs) are high-capacity secretory organs, known for production and secretion of the glue proteins that allow the pupa to adhere to a solid substrate during metamorphosis (Fraenkel and Brookes, 1953). SGs originate from a pair of ventrally positioned ectodermal placodes in the posterior head (Panzer et al., 1992) (Fig. 2Ai) that undergo dorsally directed internalization and elongation (Fig. 2Aii) (Myat and Andrew, 2000a,b), and then migrate posteriorly along the overlying visceral mesoderm (Fig. 2Aiii) to reach their final orientation and position in the embryo (Fig. 2Aiv-vi) (Bradley et al., 2003; Vining et al., 2005). SGs form simple unbranched tubes that migrate as fully polarized epithelia.

The expression patterns of the most differentially expressed genes in each Seurat cluster revealed the SG to be cluster 27 in the early set and cluster 25 in the late set (Fig. S1E,F), as supported by several SG marker genes (Fig. S2, Table S3). Gene set enrichment analysis (GSEA) of SGs compared with all other embryonic cells (Fig. 2D; Table S4) revealed that both early and late SG cells are highly enriched in gene ontology (GO) terms associated with secretory function, e.g. ‘Golgi vesicle transport’ and ‘exocrine system development’, consistent with the SG being the organ with the highest secretory capacity in the embryo and larva (Loganathan et al., 2021). Not surprisingly, the term ‘salivary gland morphogenesis’ is enriched in the early dataset, supporting correct cluster assignment, as is ‘apical junction assembly’, consistent with the cell shape changes and rearrangements that accompany early SG morphogenesis (Girdler and Röper, 2014; Myat and Andrew, 2000b; Sánchez-Corrales et al., 2021).

**Fig. 3.**
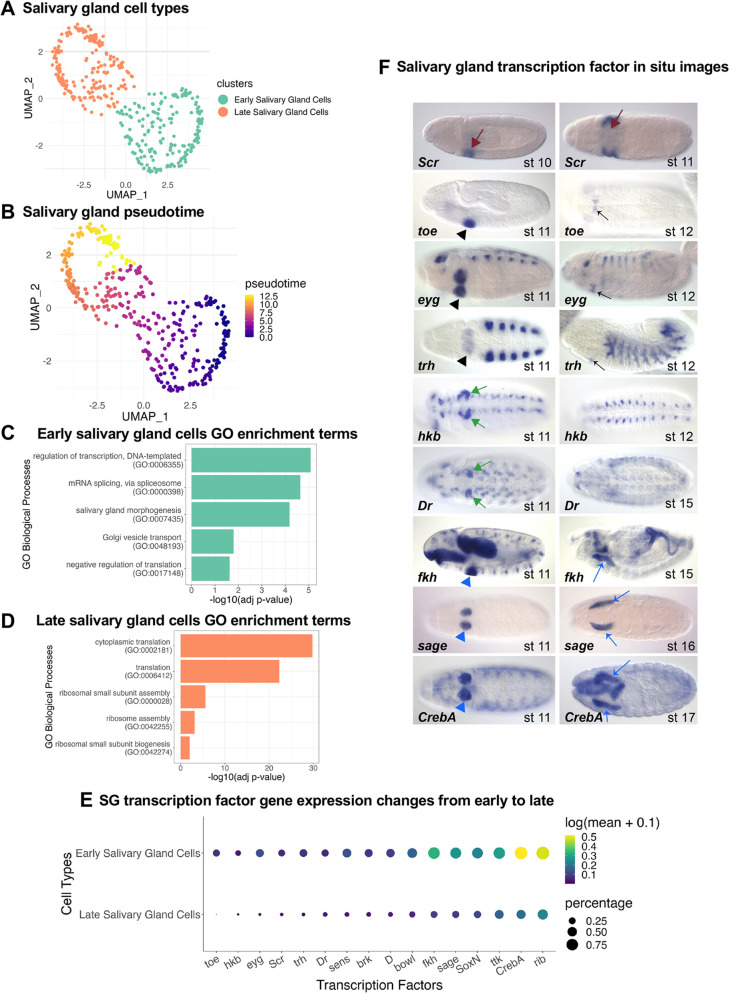
**In-depth analysis of SG cells.** (A,B) UMAP embedding of salivary gland (SG) cells colored by (A) SG sub-populations and (B) pseudotime ordering. (C,D) Selected enrichment of biological processes for (C) early and (D) late SG cells. For the full enrichment analysis results, see Table S6. (E) Expression pattern of SG-specific transcription factors (TFs) between early and late SG cells. The size of the dot represents the percentage of cells in the SG subtype in which the gene is detected; the color of the dot represents the mean expression of the gene in the SG subtype. (F) Selected *in situ* images of SG-specific TFs from the Berkeley *Drosophila* Genome Project across different stages. Black arrowheads, transient expression in the entire SG placode; black arrows, expression in the salivary duct; green arrows, transient expression in a subset of SG placode cells; red arrows, expression in SG precursors in stage 10 that disappears during stage 11; blue arrowheads, early expression of SG genes that are expressed persistently in the SG; blue arrows, late expression of SG genes that are expressed persistently in the SG.

Cluster identification and pseudotime analysis of the SG transcriptome, based on 114 cells from the stage 10-12 collections and 271 cells from the stage 13-16 collections, suggested that SG cells form a continuum (Fig. 3A,B). Based on pseudotime analysis and clustering, we identified two major clusters: early and late. The early cluster is primarily composed of stage 10-12 (both replicates) and replicate 2 of stage 13-16 cells, and the late cluster is primarily composed of stage 13-16 replicate 1 cells (Fig. 3A,B, Fig. S3A,B). Importantly, the pseudotime analysis indicates that our two stage 13-16 collections were differentially enriched for either earlier-stage (∼13-14; replicate 2) or later-stage embryos (∼15-16; replicate 1) (Fig. S3A,B). To investigate the transcriptional differences between the two clusters, we computed the differentially expressed genes (Table S5) and found that the early-stage SG transcriptome is significantly enriched for transcriptional regulation, mRNA splicing, morphogenesis and secretion (Fig. 3C, Fig. S3C, Table S6). The late-stage SG transcriptome is relatively enriched for translation, which is presumably for growth and to support secretory function (Fig. 3D, Fig. S3D, Table S6; Loganathan et al., 2016b, 2022).

Although SG secretory cells are a relatively homogeneous cell population, they express several genes in temporally or spatially restricted patterns. We identified 16 transcription factors (TFs) that are specific to SG relative to rest of the embryonic cells (percentage of expressing cells >15%; log2FC>0; Wilcoxon test adj *P*-val<0.05) in either stage 10-12 and/or stage 13-16 embryos. Among these are the HOX TF that specifies the SG (*Sex combs reduced* – *Scr*) (Andrew et al., 1994; Panzer et al., 1992) and the SP1-like TF required for spatially ordered internalization of the primordia (*hückebein* – *hkb*) (Myat and Andrew, 2000b, 2002; Sánchez-Corrales et al., 2021). Also among the temporally regulated SG TFs are those initially expressed in the entire primordium (duct and secretory cells) that later become restricted to only duct [*twin of eyegone* (*toe*), *eyegone* (*eyg*) and *trachealess* (*trh*)] (Isaac and Andrew, 1996; Jones et al., 1998). Other SG TF genes, especially those regulating SG physiology, are activated early and continue to be expressed in all SG cells through metamorphosis and in the adult SG [*Cyclic-AMP response element binding protein A* (*CrebA*), *fork head* (*fkh*), *sage* and *senseless* (*sens*)] (Abrams and Andrew, 2005; Abrams et al., 2006; Chandrasekaran and Beckendorf, 2003; Fox et al., 2010, 2013; Zhou et al., 2001). Consistent with the known patterns, we find expression of *Scr*, *hkb*, *toe*, *eyg* and *trh* in only the early SG secretory cells, whereas expression of *CrebA*, *fkh*, *Sage* and *sens* is in both the early and late SG (Fig. 3F,E). Our analysis also uncovered additional TFs heretofore not known to be expressed in the SG (Fig. 3E, Table S2), including two that are expressed only in the early gland – *Dichaete* (*D*) and *Drop* (*Dr*). Interestingly, *Dr* is expressed only early and only in the subset of SG cells that internalize first (Fig. 3E). Thus, although our scRNA-seq captured fewer SG cells than expected based on the known number of SG cells (∼140) and estimates of the number of embryonic cells (∼6000–8000), our read depth was sufficient to identify many new SG genes, including those expressed only transiently and/or in only a subset of SG cells.

### Embryonic tracheal single cell transcriptome reveals the regulatory signature for growth in branching morphogenesis

The *Drosophila* trachea is an ectodermal derivative that forms from ten pair of ectodermal placodes (segments T2 through A8) (Fig. 2Bi) (Loganathan et al., 2016a). Placodal cells invaginate to form rudimentary epithelial sacs (Fig. 2Bii) that subsequently extend segmentally restricted primary branches that migrate along distinct trajectories to eventually reach cells throughout the animal (Fig. 2Biii-iv) (Samakovlis et al., 1996). Tracheal fusion at the ends of many branches (Fig. 2Bv) connects the individual tracheal segments into a contiguous tubular network for gas exchange (Fig. 2Bvi). Although trachea undergo branching morphogenesis, they migrate as fully polarized epithelia like the SG.

Cluster 10 in the early UMAP and cluster 16 in the late UMAP correspond to tracheal cells based on the expression of key marker genes (Fig. 1B,D, Fig. S4, Table S3). Both early and late tracheal cells were enriched in the following GO terms: open tracheal system development, tube size regulation, cell-cell junctions and epithelial cell apical/basal polarity (Fig. 2D, Table S4). Early trachea was also enriched in GO terms associated with growth and morphogenesis, whereas late trachea was enriched in Golgi vesicle transport and liquid clearance, consistent with processes that occur late in tracheal maturation (Tsarouhas et al., 2007). mRNA splicing terms and ATP synthesis were relatively lower in the trachea compared with other embryonic cells (Table S4).

**Fig. 4.**
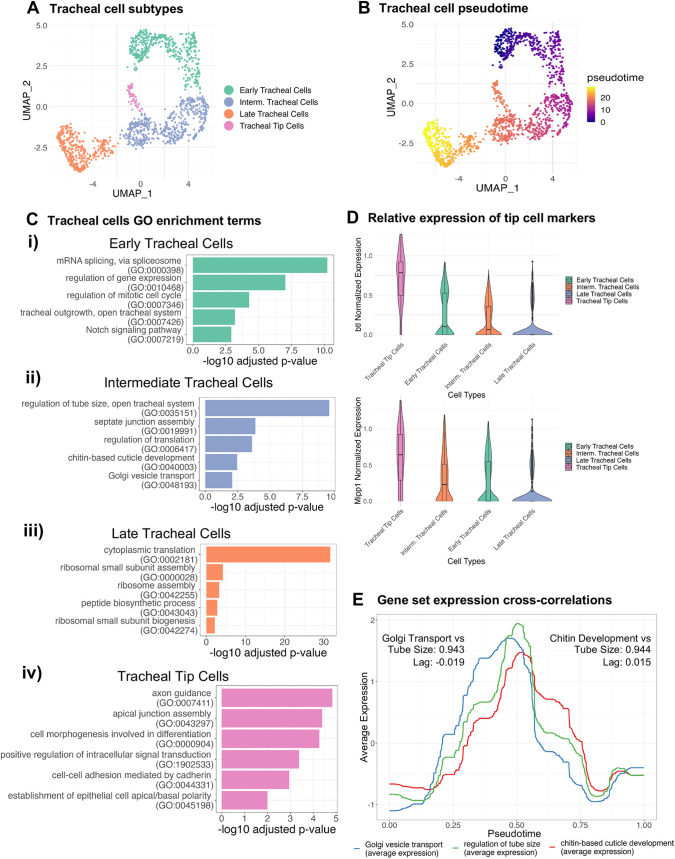
**In-depth analysis of tracheal cells.** (A,B) UMAP embedding of tracheal cells colored by (A) tracheal sub-populations and (B) pseudotime ordering. (C) Selected enrichment of biological processes for different sub-populations of tracheal cells: (i) early tracheal cells, (ii) intermediate tracheal cells, (iii) late tracheal cells and (iv) tracheal tip cells. For the full enrichment analysis results, see Table S8. (D) Normalized expression of tracheal tip cell markers *btl* and *Mipp1* across different tracheal sub-populations. (E) The dynamic time-series of averaged and scaled gene set expression of ‘Golgi vesicle transport’, ‘regulation of tube size’ and ‘chitin-based cuticle development’ across pseudotime. The cross-correlation value and lag between Golgi Transport and Tube Size is on the left, and the cross-correlation value and lag between Chitin Development and Tube Size is on the right.

Cluster identification and pseudotime analysis of tracheal cells, based on 522 cells from stages 10-12 and 1003 cells from stages 13-16, not only confirmed the continuum of developmental stages (Fig. 4A,B), paralleling our observations from the SG transcriptome data, but also allowed us to categorize nine clusters into the following cell subtypes: (1) early, (2) intermediate and (3) late tracheal cells, along with a fourth category that captured a subset identifiable as tracheal tip cells (Fig. 4A, Fig. S5A). As observed in SGs, the early tracheal cells are mainly composed of cells from the replicates of stage 10-12, the late tracheal cells are mainly composed of cells from stage 13-16 replicate 1, and intermediate tracheal cells are mainly composed of cells from stage 13-16 replicate 2 (Fig. S5B,C).

We performed differential expression analysis on the different tracheal subpopulations (Table S7) and found the biological processes enriched in different subpopulations (Fig. 4C, Table S8). The early tracheal cell transcriptomes were enriched for mRNA splicing, regulation of mitotic cycle and tracheal outgrowth (Fig. 4Ci). The intermediate cell subtype transcriptomes were enriched for biological processes integral to tracheal branching morphogenesis, such as regulation of tube size, septate junction assembly, chitin-based cuticle development and Golgi vesicle transport (Fig. 4Cii). Transcriptomes of the late cell subtype, moreover, were predominantly enriched for translation, likely supporting the apical secretions and corresponding diametrical size increases that occur at late stages (Fig. 4Ciii) (Beitel and Krasnow, 2000).

The tip cell population is a highly dynamic cell subtype found at the ends of migrating branches and was identified within tracheal cell clusters by its high levels of expression of the migratory markers *breathless* (*btl*), which encodes a tracheal-specific fibroblast growth factor receptor (Klämbt et al., 1992), and *Multiple inositol polyphosphate phosphatase 1* (*Mipp1*), a gene that increases the filopodial extensions in tip cells to facilitate migration (Fig. 4D) (Cheng and Andrew, 2015). The transcriptomes of this cell subtype are almost evenly enriched for junction assembly, morphogenesis, cell-cell adhesion and regulators of intracellular signal transduction (Fig. 4Civ). Moreover, tip cells are also enriched in axon guidance terms, suggesting that similar molecular programs are used in cell migration and in elongating axons (Fig. 4Civ) (Aberle, 2019). Although the tip cells have representation from both stage 10-12 and stage 13-16, there is a separation of cells from the different time points (Fig. S5B). Among the top differentially expressed genes *Bearded* (*Brd*), a regulator of Notch signaling that could facilitate fate choices among tip cells, is enriched in stage 10-12 tip cells (Fontana and Posakony, 2009; Ikeya and Hayashi, 1999) (Fig. S5D). Furthermore, *verm* and *serp* are enriched in stage 13-16 tip cells, which we might expect based on their roles in tube elongation and growth (Luschnig et al., 2006) (Fig. S5D).

Together, the tracheal organogenetic transcriptome reveals the significance of tube size regulation during branching morphogenesis of a vital organ (Fig. 2D, Table S4). As the tubules permeate the entire embryonic volume to ensure gas exchange with every cell, genetic mechanisms for the fine-tuning of tubule size (from ∼60 µm for the largest diameter tubules to ∼100 nm for the smallest) are expected to play a leading role in directing morphogenesis (Beitel and Krasnow, 2000). Tracheal tube size regulation is accomplished by coordinated regulation of (1) apical vesicle trafficking, (2) chitin production, modification and structural transformation within the lumen, and (3) organization and function of the septate junctions (Hayashi and Dong, 2017). Thus, we asked whether these functionalities are indeed correlated in the single-cell dataset. We used cross-correlation (Derrick and Thomas, 2004), a metric used to measure the temporal association between two time-series signals to: (1) find the correlation between the average expression along the pseudotime trajectory of the two gene sets and (2) find the lag at which the correlation between the average expression of the two gene-sets is the strongest. The latter metric would inform us which biological function initiates/leads and which follows.

Our results provide evidence for high cross-correlation between Golgi vesicle transport gene set expression dynamics and regulation of tube size gene set expression dynamics (Fig. 4E). The high correlation suggests that the two gene sets have similar average dynamics across pseudotime with a negative lag, indicating that vesicle transport leads the biological process for tube size regulation despite the absence of overlapping genes between sets (Fig. S6A,B). Another high correlation was observed between chitin development and tube size regulation (Fig. 4E), with the expression of chitin-based cuticle development genes trailing that of genes implicated in tube size regulation. Unlike with the Golgi transport genes, the chitin-based cuticle development gene set contains seven genes also in the set for tube size regulation (Fig. S6A,C). Altogether, our analysis of the tracheal organogenetic transcriptome reveals the subtle dynamics that underlie growth in branching morphogenesis, while simultaneously capturing genes preferentially expressed in the highly dynamic tip cell population.

### Embryonic germ cell transcriptome reveals two developmental trajectories and suggests a role for proteolysis in the germline

Germ cells (GCs) arise at the posterior end of the embryo (Fig. 2Ci) (Dansereau and Lasko, 2008). During germ band extension, they are passively internalized into the posterior midgut pocket (Fig. 2Cii) (Warrior, 1994). GCs then migrate dorsally through the midgut endoderm into the overlying mesoderm (embryonic stages 9-10) (Fig. 2Ciii-v), subsequently forming separate populations, one on each side of the midline (Fig. 2Cvi). GC migration ceases once GCs and SGPs coalesce to form the gonads (Fig. 2Cviii) (Warrior, 1994). The position of GCs within the coalesced gonads (Fig. 2Cix) determines which cells eventually form the adult germline stem cells and which cells either begin to differentiate (male GCs) or go into a ‘holding pattern’ until pupal stages (female GCs) (Dansereau and Lasko, 2008).

GCs were identified as two nearby clusters in the early UMAP (clusters 24 and 32) and in a single split cluster in the late UMAP (cluster 12) (Fig. S1B,D-F) based on expression of known GC marker genes (Fig. S7, Table S3). GSEA of stage 10-12 GCs revealed significant enrichment of terms associated with two major processes relative to rest of the embryo (Fig. 2D): (1) proteolysis (both ubiquitin-dependent and ubiquitin-independent) and (2) ATP-coupled transport. GSEA of stage 13-16 GCs revealed proteolysis as the major process represented in GCs relative to all other cells (Fig. 2D, Table S4).

**Fig. 5.**
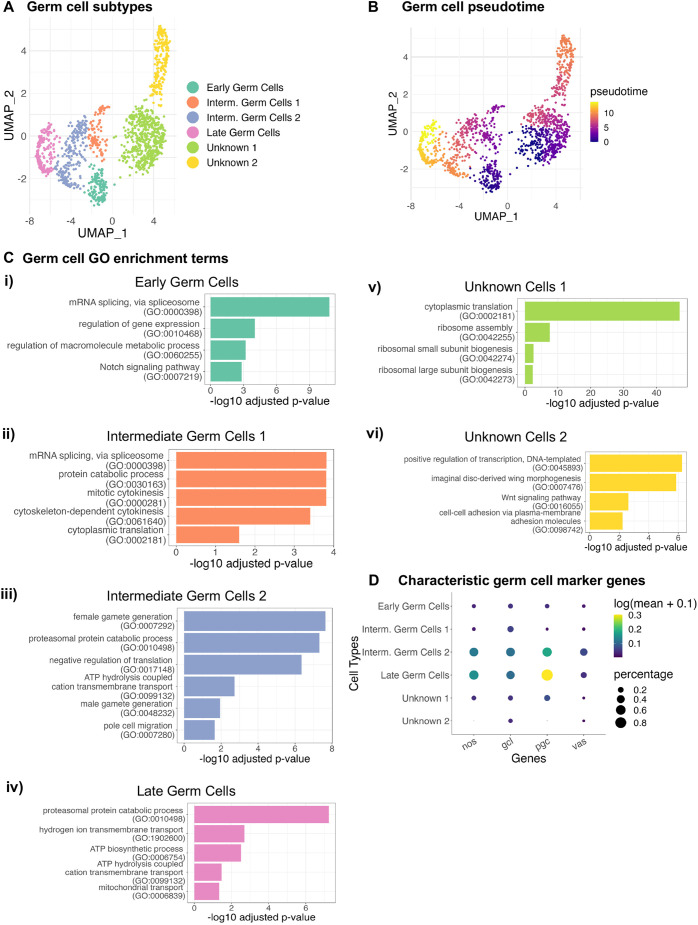
**In-depth analysis of germ cells.** (A,B) UMAP embedding of germ cells (GCs) colored by (A) GC sub-populations and (B) pseudotime ordering. (C) Selected enrichment of biological processes for different GC sub-populations: (i) early germ cells, (ii) intermediate germ cells 1, (iii) intermediate germ cells 2, (iv) late germ cells, (v) unknown cells 1 and (vi) unknown cells 2. For the full enrichment analysis, see Table S10. (D) Dot plot showing the normalized expression and percentage expression of characteristic germ cell marker genes. The size of the dot represents the percentage of cells in the GC subtype in which the gene is detected; the color of the dot represents the mean expression of the gene in the GC subtype.

To learn how distinct expression profiles correlate with the different stages and populations of embryonic GCs, we clustered cells within the larger GC populations from both stages. This analysis revealed six sub-populations of cells that populate two distinct trajectories (Fig. 5A, Fig. S8A). Pseudotime analysis (Fig. 5B) shows that the likely earliest cluster – ‘early GCs’ – is populated by cells from the stage 10-12 replicates with cells from replicate 2 contained almost entirely within this cluster (Fig. S8B). Stage 10-12 replicate 1 includes cells that also populate ‘intermediate cluster 1’ and ‘intermediate cluster 2’ (Fig. S8B). Much as observed in SGs and trachea, cells from stage 13-16 replicate 2 (Fig. S8B,C) reside between stage 10-12 and stage 13-16 replicate 1 on the continuum and primarily populate intermediate cluster 2. What we refer to as unknown clusters 1 and 2 comprise cells almost entirely from stage 13-16 replicate 1, our latest sample, based on the pseudotime analysis of the SG and trachea. Pseudotime analysis suggests that cells in cluster unknown 1 are earlier than cells in cluster unknown 2 (Fig. 5B).

As for SG and trachea, we performed differential expression analysis (Table S9) and gene set enrichment analysis (GSEA) (Table S10) on the different subpopulations of GCs. GSEA comparing each GC cluster revealed that early GCs show relatively high enrichment of splicing, transcription, macromolecular biosynthesis and morphogenesis (Fig. 5Ci, Table S10). Intermediate cluster 1 GCs are enriched in terms associated with splicing, cell division and translation (Fig. 5Cii). Importantly, protein degradation is an enriched process in the intermediate 1 cluster and this process is also highly enriched in both the intermediate 2 and late GC clusters (Fig. 5Cii-iv). Intermediate cluster 2 GCs are relatively enriched in gametogenesis and pole cell migration (Fig. 5Ciii). Although both female and male gamete generation-related processes are enriched in this cluster, female gamete generation processes are more significantly enriched. Finally, both intermediate cluster 2 GCs and late GCs are enriched in ATP (biosynthesis and usage) processes (Fig. 5Ciii,iv). The most enriched process for unknown cluster 1 is translation, with ribosomal protein genes and genes encoding factors involved in the maturation of ribosomal RNAs showing the most significant enrichment (Fig. 5Cv). On the other hand, the most significantly enriched processes in unknown cluster 2 include transcription, morphogenesis, signaling and genes involved in junction assembly (Fig. 5Cvi).

To determine what cell types correspond to the different GC clusters, we examined expression of GC genes that are maternally encoded and play key roles in GCs; *nanos* (*nos*), *germ cell-less* (*gcl*), *polar granule component* (*pgc*) and *vasa* (*vas*) all show highest levels of expression in intermediate 2 and late GCs (Fig. 5D, Fig. S9A). Although late GC expression of both *gcl* and *pgc* is unexpected, both *nos* and *vas* are known to be zygotically expressed in GCs of late-stage embryos (Van Doren et al., 1998; Yatsu et al., 2008), consistent with this trajectory representing germline stem cells (Fig. 5A). Similarly, two genes known to be differentially expressed in the female germline stem cells (*ovo* and *otu*) exhibit relatively higher-level expression in the intermediate 2 and late GC clusters (Fig. S9B). Notably, although expression of several of the male GC genes are relatively lower in unknown cluster 1, their expression levels are high in unknown cluster 2 (Fig. S9C). In contrast, the female germline genes show the lowest relative expression of all in unknown cluster 2 (Fig. S9B). Perhaps also noteworthy, the early GC cluster and both unknown clusters had the smallest number of UMIs (Fig. S9D). This finding suggests that the unknown clusters had relatively fewer transcripts and may be lower quality or contain more differentiating cells. Based on pseudotime analysis and on observations from other labs regarding the link between increased translation and GC differentiation (Sanchez et al., 2016), we propose that unknown cluster 1 includes GCs destined to differentiate earlier and not become germline stem cells in the adult. Based on the relative levels of expression of male transcripts in unknown cluster 2, including two that are upregulated in spermatogonia (*CG6701* and *Pp2C1*), we hypothesize that cells in this cluster are male GCs that will differentiate into spermatogonia, a process that likely commences at the end of embryogenesis, based on the presence of spermatogonia in first instar larva (Fuller, 1993).

Collectively, our GC clustering, pseudotime and GSEA results confirm processes known to direct GC differentiation while highlighting the potential contribution of less well studied processes (protein catabolism and translation) and cell populations in germline formation.

### Tissue-specific sources of the *Drosophila* matrisome

Access to transcripts expressed in every tissue of the developing embryo will inform future studies on the formation and specialization of individual organs. Indeed, we have explored the gene sets expressed at crucial stages in the formation of the SG, trachea and germline. Organogenesis, however, also involves extracellular constituents (Abrams et al., 2006; Devine et al., 2005; Serna-Morales et al., 2023; Walma and Yamada, 2020). Hence, to further test the power and versatility of our organogenetic transcriptomes, we used our data to identify the embryonic cellular sources of expression for the matrisome – the ensemble of 641 genes expressing the *Drosophila* extracellular matrix (ECM) and ECM-associated proteins (Davis et al., 2019). The matrisome includes genes encoding core ECM proteins (collagens, glycoproteins and proteoglycans), matrix-associated proteins, ECM regulators (enzymes that modify ECM proteins) and a variety of secreted substances, including glue proteins, chorion and vitelline membrane components, mucin-like proteins and the chitinous materials that form the exoskeleton. To learn which embryonic cell types express different matrisome, we analyzed relative expression levels of selected matrisomal genes in our stage 10-12 and stage 13-16 datasets (Tables S11, S12).

**Fig. 6.**
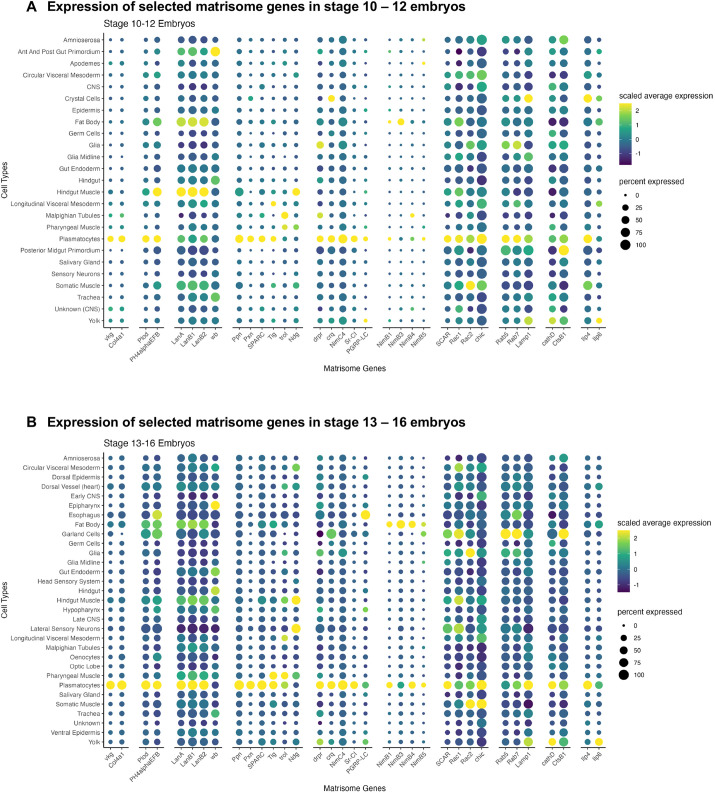
**In-depth analysis of selected matrisomal genes across stage 10-12 and stage 13-16 embryonic cell types.** (A,B) Dot plots of selected matrisomal genes across embryonic cell types in (A) stage 10-12 embryos and (B) stage 13-16 embryos. The size of the dot represents the percentage of cells in the embryonic cell type in which the gene is detected; the color of the dot represents the mean expression of the gene in the embryonic cell type.

Many matrisomal genes are expressed in the different cell types identified in our early and late clusters (Fig. 6A,B). The *Drosophila* ‘blood cells’, known as hemocytes, include two cell types in the embryo: the abundant plasmatocytes, which function in phagocytosis and the production of anti-microbial peptides (AMPs); and the less abundant crystal cells (<5% of hemocytes), which produce the enzymes required during larval stages for the deposition of melanin around parasites and wounds (Rizki and Rizki, 1959; Schulz and Fossett, 2005; Vlisidou and Wood, 2015). Accordingly, we observe high level expression of a wide range of genes required for phagocytosis, recovering many of the same genes identified by others in bulk sequencing of purified plasmatocytes from stage 16 embryos (Fig. 6A,B) (Cattenoz et al., 2020). Another function of plasmatocytes during embryogenesis and larval stages is the production of the specialized ECM, known as the basement membrane or basal lamina, which surrounds and compartmentalizes different tissues and organs (Olofsson and Page, 2005). Thus, it is not surprising to find that plasmatocytes express the highest levels of the two major collagen genes produced in embryos, *viking* and *Col4a1*, and of the genes encoding the enzymes required for collagen maturation: *Plod*, which encodes procollagen lysyl hydroxylase, and *PH4αEFB*, which encodes a prolyl-4-alpha hydroxylase (Fig. 6A,B). These ER-localized enzymes hydroxylate the lysines and prolines on collagen propeptides – post-translational modifications that are required for the cross-linking and secretion of collagen. Hindgut muscle and fat body express high levels of genes encoding the other major constituents of the basal lamina, the laminins (LanA1, LanB1 and LanB2) (Sánchez-Sánchez et al., 2017), during embryonic stages 10-12, whereas plasmatocytes express the highest levels of these transcripts in stage 13-16 embryos (Fig. 6A,B). The other Laminin A gene [*wingblister* (*wb*)] is expressed to highest levels in the anterior and posterior midgut primordia at early stages and in the esophagus at late stages (Fig. 6A,B). Additional basement membrane proteins and ECM glycoprotein genes (*Ppn*, *Pxn* and *SPARC*) are also most highly expressed in plasmatocytes (Fig. 6A,B). The ECM glycoprotein gene *Tiggrin* is expressed at high levels in both pharyngeal muscle and plasmatocytes. Perlecan and Trol are expressed at their highest levels in pharyngeal muscle. The Nidogen gene is expressed at its highest levels in the hindgut muscle at early stages and in both hindgut muscle and lateral sensory neurons at late stages (Fig. 6A,B).

We observe plasmatocyte expression of many genes implicated in phagocytosis (Melcarne et al., 2019), including genes encoding phagocytic receptors and opsinins (secreted molecules that bind microbes to promote efficient uptake by phagocytic cells). The phagocytic receptor genes include those encoding Draper (*drpr*) and Croquemort (*crq*), which recognize bacteria and apoptotic cells, Nim4C, which is specific to apoptotic cells, and dSr-Cl and PGRP-LC, which are bacteria specific (Fig. 6A,B). Several secreted Nim genes (*NimB1*, *NimB3*, *NimB4* and *NimB5*) are expressed in a much higher percentage of cells in later stage plasmatocytes than in early (Fig. 6A,B). Factors involved in engulfment, including SCAR, Rac1, Rac2 and profilin (*chic*), are highly upregulated in plasmatocytes at both stages. Two Rabs implicated in phagosome maturation, Rab5 and Rab7, are also expressed in plasmatocytes, as is LAMP1, a lysosomal protein needed for fusion of the lysosome and phagosome. Altogether, these findings suggest plasmatocytes begin expressing components of the ECM/basal lamina and key mediators of phagocytosis quite early.

Although our transcript analyses fully support the roles of embryonic plasmatocytes in phagocytosis and secretion of the basal lamina, we find no evidence that these cells express the anti-microbial peptides (AMPs) genes that they produce at later stages (Ferrandon et al., 2007). Transcripts for Drosomycin, Drosocin, Diptericin, Bomanins, Cecropins, Defensins and Metchnikowin are undetectable in plasmatocytes (Fig. S10A,B). This is in keeping with the protected (sterile) environment of the embryo inside two protective layers (the chorion and vitelline membranes) and could suggest that the phagocytic activity of embryonic plasmatocytes is primarily the engulfment of apoptotic cells.

Our analysis of the matrisome also revealed that, quite unexpectedly, two insulin-like protein genes, *Ilp4* and *Ilp6*, are expressed most highly in embryonic hemocytes (both in plasmatocytes and crystal cells) and in yolk cells, respectively (Fig. 6A,B). The SG, fat body and other tissues are the major sources of these growth-promoting hormones during larval stages (Brogiolo et al., 2001; Okamoto et al., 2009), and the fat body is the primary source of Ilp6 in adults, based on scRNA-seq from Fly Cell Atlas (Li et al., 2022) (Fig. S11A-C). Perhaps having the abundant yolk and widely distributed hemocytes as the major sources of these growth-promoting hormones ensures their availability across all tissues in embryonic and early larval stages. Although the growth of embryonic cells has not, for the most part, been measured, we have discovered that both SG and tracheal cells double in volume during embryogenesis (Loganathan et al., 2022).

Our matrisome analysis also revealed the dynamics of transcripts for cuticle synthesis and processing during embryogenesis. During stages 10-12, the apodemes (which function as ‘tendons’ to attach muscles to the exoskeleton) express both the highest levels and highest variety of these genes (Fig. S12). High-level expression of subsets of cuticle genes is detected in the esophagus, hypopharynx and lateral sensory neurons during only stages 13-16 (Fig. S13), suggesting that the transcriptome for cuticle synthesis and secretion is tissue and stage specific. Supporting this idea, we did not observe high expression of cuticle protein genes in epidermal cells of either early or late embryos. This suggests that cuticle genes are not highly expressed in the epidermis until immediately before the synthesis and secretion of the larval cuticle (stage 17). In summary, detailed analysis of our embryonic single cell transcriptomes reveals both expected and unexpected findings that provide potential insight into ECM-mediated cellular functions during organogenesis.

### Transcriptome comparisons reveal that the majority of the embryonic cell lines are most similar to plasmatocytes

*Drosophila* embryonic cell lines are commonly derived spontaneously from primary cultures of embryos (Simcox et al., 2008). As a result, the tissue of origin is often unclear, an issue further complicated by the difficulty of cleanly isolating embryonic tissues for comparison. Here, we use scRNA-seq data to identify the likely tissue of origin by assessing transcriptome similarities between embryonic cell lines and primary tissues using our scRNA-seq data. We downloaded bulk RNA-seq data of five embryonic cell lines, S3, S1, Kc167, GM2 and 1182.4H from FlyBase (Gramates et al., 2022). To ensure compatible comparisons of scRNA-seq and bulk expression profiles, we averaged the expression profiles of all the cell types in our stage 10-12 and stage 13-16 scRNA-seq data into *pseudo-bulk* expression profiles that serve as a cell type reference expression matrix with 1604 unique tissue marker genes. To test the capability of our cell type reference expression matrix, we gathered 27 expression profiles from adult *Drosophila* tissues (Li et al., 2022) and computed Spearman's rank correlation coefficients with the reference matrix (Fig. S14A,B). Overall, the top correlated tissues from the scRNA-seq data match well with the annotations of bulk expression data, except for adult midgut and adult/larval salivary gland (Fig. S14B).

**Fig. 7.**
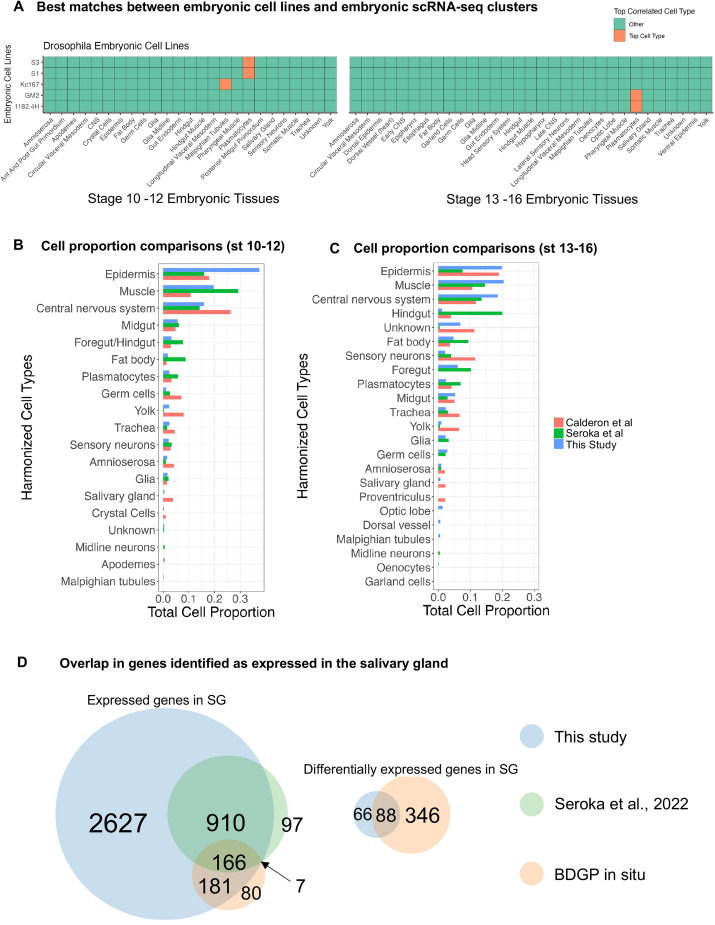
**Alignment of *Drosophila* embryonic cell line transcriptional profiles with transcriptional profiles of embryonic cell types in stage 10-12 and stage 13-16.** (A) Heatmap showing the best match between the bulk expression of embryonic cell lines and (stage 10-12 and stage 13-16) the pseudo-bulk expression of embryonic scRNA-seq cell types. The orange squares represent the cell type with which the embryonic cell lines are most correlated in terms of the transcriptional profiles of 1604 tissue marker genes. (B,C) Cell type proportion comparisons across three single-cell datasets: Calderon et al. (2022), Seroka et al. (2022) and this study for cells in (B) stage 10-12 and (C) stage 13-16. (D) Comparison of the number of genes detected in SG cells from this study, Seroka et al. (2022) and the Berkeley *Drosophila* Genome Project *in situ* hybridizations.

We then computed Spearman's correlations between the expression profiles of the query embryonic cell-line and the cell type reference expression matrix. We found that four out of five embryonic cell lines are most correlated with plasmatocytes (Fig. 7A, Fig. S14C). S3 and S1 correlate most with stage 10-12 plasmatocytes, and GM2 and 1182.4H correlate most with stage 13-16 plasmatocytes (Fig. 7A). Surprisingly, although Kc167 was previously found to have plasmatocyte-like properties (Klonaros et al., 2023), we found that it is transcriptionally most like Malpighian tubules (Fig. 7A).

### Cross-validation of the organogenetic transcriptome with other *Drosophila* embryonic scRNA-seq datasets

At least four major single cell sequencing datasets have been generated from *Drosophila* embryos (Calderon et al., 2022; Karaiskos et al., 2017; Sakaguchi et al., 2023; Seroka et al., 2022); two of those overlap the stages examined in our study (Calderon et al., 2022; Seroka et al., 2022). Calderon et al. acquired continuum single nucleus RNA sequencing data for embryogenesis and focused their analyses on resolving the transcriptional regulatory dynamics at high temporal resolution. Seroka et al. acquired data from embryonic stages 12-16, comparable with our organogenesis datasets, focusing primarily on *Drosophila* neurogenesis. To contextualize and compare our data with these two previously published datasets, we performed a suite of comparative analyses, beginning with tabulation of the key features of raw data acquisition that showed the read depth of our data was comparable with that of Seroka et al. and five- to tenfold higher than that of Calderon et al. (Table 1).

**Table 1.**
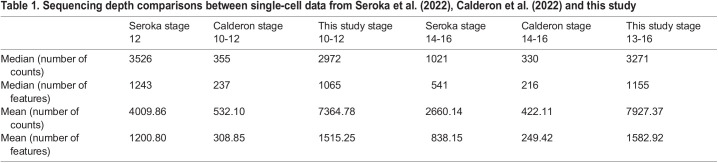
Sequencing depth comparisons between single-cell data from Seroka et al. (2022), Calderon et al. (2022) and this study

To ensure fair comparisons of cell types across all three datasets, we harmonized and/or unified the different cell labels across the three datasets (Table S13). Harmonized cell type comparisons show that, all three datasets largely capture epidermis, muscle and central nervous system as the top three clusters with the highest proportions of cells (Fig. 7B,C). Different from the other two studies, the hindgut cell proportion from the Seroka et al. (2022) (stage 14-16) dataset is relatively higher, whereas the epidermis proportion is relatively lower (Fig. 7C).

Next, to compare the agreement of cell type annotations across the three datasets, we used the post-harmonization data from the other two studies as reference data to train SingleCellNet (SCN) classifiers (Tan and Cahan, 2019). Remarkably, our cell typing results largely agree with those of Seroka et al. (Seroka et al., 2022), except for the foregut/hindgut (hindgut and anterior/posterior gut primordium), which were classified as a mix between midgut and epidermis in early stages (Fig. S15A). The foregut (epipharynx, hypopharynx and esophagus) and hindgut from our data, moreover, are classified as epidermis in late stages using Seroka et al.’s data as a reference (Fig. S15B). The SCN classifier trained on Calderon et al. (2022), however, showed inconsistencies between our cell typing and theirs (Fig. S15A,B); the misclassification is due likely to differences in sequencing depth (Table 1).

In addition to the major cell types identified by all datasets, many cell types were detected only in our dataset, which we validated with the *in situ* data from the Berkeley Drosophila Genome Project (BDGP) (Tomancak et al., 2007; Weiszmann et al., 2009). The rare cell types identified from our early-stage organogenetic transcriptomes that were missing in at least one of the other datasets are: (1) apodemes (Fig. S16), (2) SG (Fig. S17), (3) Malpighian tubules (Fig. S18) and (4) crystal cells (Fig. S19). The rare cell types identified from our late-stage organogenetic transcriptomes that were missing in at least one of the other datasets are: (1) optic lobe (Fig. S20), (2) SG (Fig. S21), (3) dorsal vessel (Fig. S22), (4) Malpighian tubules (Fig. S23), (5) oenocytes (Fig. S24), (6) garland cells (Fig. S25), (7) epipharynx (Fig. S26), (8) esophagus (Fig. S27), (9) hypopharynx (Fig. S28), (10) hindgut (Fig. S29), (11) hindgut muscle (Fig. S30) and (12) pharyngeal muscle (Fig. S31).

Two possibilities could account for the absence of these rare cell types from the previously published organogenetic transcriptomes (Calderon et al., 2022; Seroka et al., 2022): (1) the rare cells may have been lost either during the cell isolation steps or subsequent sequencing steps and/or (2) the rare cell types may have been either missed during the annotation step or misannotated. To determine whether the rare cell types exist in the published datasets under different labels, we used our early- and late-stage datasets and their corresponding labels as training data for SCN classifiers, and applied them to the previously published datasets. Using this approach, we successfully identified crystal cells but not apodemes, SG cells or Malpighian tubules in the stage 12 single cell transcriptomes from Seroka et al. (Fig. S32A-D). The crystal cells identified by our SCN classifiers are a small population previously annotated as hemocytes (Seroka et al., 2022). We identified a tiny population of apodeme cells in Calderon et al. (Fig. S32E). Our SCN classifiers, moreover, successfully detected the presence of small clusters of cells for optic lobe, SG, dorsal vessel, Malpighian tubules, oenocytes and garland cells from Seroka et al.’s datasets for stages 14 and 16 (Fig. S33). Our cross-validation analysis with the data from Seroka et al. suggests that these rare cell types were previously annotated under more general labels, with some even falling into the unknown category (oenocytes). With the stage 14-16 datasets from Calderon et al., however, our SCN classifiers were not able to identify sufficient cells that resemble any of the rare cell types (Fig. S34).

Finally, to test the gene capture efficacy of our single-cell organogenetic data, we compared the genes captured by scRNA-seq technology with *in situ* data from the BDGP. We chose the SG to perform this test because of the transcriptional homogeneity in the tissue and the ease of its identification (simple morphology) both early and late by *in situ*. For the analysis, we selected all genes that were expressed in at least 10% of the SG cells in our scRNA-seq data from either stage 10-12 or stage 13-16 and all the genes that were expressed in at least 10% of the SG classified cell clusters (Fig. S33B) from all three timed collections in Seroka et al. (2022), and compared with genes expressed in the SG based on *in situ* data from the BDGP (Fig. 7D). In total, we identified 3884 SG expressed genes in our scRNA-seq data, 1180 SG expressed genes in the Seroka et al. data and 434 genes annotated as expressed in SG from the BDGP (Fig. 7D). Broadly, 91% of the SG genes (1076/1180) from the Seroka et al. data and 80% (347/434) of the BDGP genes were captured in our scRNA-seq data. Notably, only seven BDGP genes not found in our data were found in the Seroka et al. data, whereas 80 BDGP genes were not captured in scRNA-seq data. Furthermore, from the SG expressed genes in our dataset, we identified 154 genes that were significantly upregulated in the SG compared with all other cell types (*P*_adj_≤0.05), a conservative estimate of SG-specific genes. Among these, 88 genes (57%) were identified by BDGP as SG expressed. Collectively, this demonstrates that scRNA-seq can capture most expressed genes in tissues found by *in situ* hybridization (80%) and can be used as a complementary high-throughput method to *in situ* hybridization for studying gene expression across tissues.

## DISCUSSION

In this study, we acquired single cell transcriptomes capturing organogenesis in the *Drosophila* embryo. Our initial analysis of the data from three organs – the SG, trachea and the germline cells (GCs) – provides glimpses into the potential of these datasets. The data can reveal when specific cellular and biochemical processes that are crucial for morphogenesis and physiological changes ensue. They can reveal entire gene expression programs for each organ as well as for specialized cell types within organs and they can expose previously unappreciated processes likely to be crucial for cellular programming. Through the interrogation of the expression of single genes across cell types, our data provide insight into the cellular origins of the embryonic matrisome – a key component of organ function and mechanics. Finally, by creating pseudo-bulk expression profiles from our scRNA-seq data, we identified the likely cell type of origin for commonly used *Drosophila* tissue culture cell lines.

### Transcriptomic analysis of somatic tissues reveals temporal details in the expression of TFs and downstream events

Our analyses of SG gene expression reveals that the most enriched process in the SG compared with all other cell types is secretion. For both staged collections, the top three enriched biological processes of the SG transcriptomes contain genes encoding core secretory machinery (Table S4). This is not surprising; we have previously shown this gene set to be upregulated in the SG through microarray and *in situ* studies, where we have further demonstrated that genes encoding the secretory machinery are under the direct control of a single early and continuously expressed TF – CrebA (Abrams and Andrew, 2005; Fox et al., 2010; Johnson et al., 2020). The upregulation of the translation machinery (e.g. ribosomal protein genes), which shows a slight temporal lag relative to secretory function based on pseudotime ordering (Fig. S3C,D), likely provides for the corresponding increases in translation that support increased secretory load. Although increases in translation also support cellular growth, we suspect that the heightened enrichment of this process in the late SG more likely reflects its support of secretory function, as SG cell growth is continuous during embryonic development (Loganathan et al., 2022). Our examination of SG TF expression reveals that, although we recovered fewer SG cells than expected at both stages, we easily detected TF transcripts that were only transiently expressed, some of which were also expressed in only a subset of SG cells. Importantly, 91% of SG transcripts derived from the Seroka et al. (2022) data and 80% of SG annotated genes from BDGP *in situ* data were included in our set. Importantly, sharing our findings with the BDGP lead to the re-examination and re-annotation of several genes; *CG2663*, *CG11052*, *SodH1*, *CG10365*, *Slc45-1*, *ng1* and *Inx7* are no longer listed as SG expressed. Three genes were re-annotated to indicate their expression in the adult SG primordia (imaginal ring) and not in the embryonic SG: *CG2157*, *CG43394* and *Tsp74F*. Finally, the BDGP team carried out *in situ* hybridizations with several SG DE genes from our dataset and found that six had clear SG expression (*Tpst*, *pio*, *CG31808*, *CG33169*, *CG34190* and *CG46385*). *In situ* hybridization images for these genes are now available at the BDGP (https://insitu.fruitfly.org/cgi-bin/ex/insitu.pl).

Tracheal growth is tightly regulated and crucial for tracheal function, with the large bore tubes (dorsal trunk) being close to the posterior spiracles, the source of atmospheric gases, and the small bore tubules, which carry out gas exchange, being closest to the target tissues (Beitel and Krasnow, 2000; Samakovlis et al., 1996). Processes required for diametrical expansion of the tracheal lumen have been linked to the canonical secretory pathway (Jayaram et al., 2008) and to the production and secretion of a chitin-based apical ECM (Devine et al., 2005). Tube length regulation, on the other hand, has been linked to expression of chitin deacetylases (enzymes involved in the modification of the chitinous apical ECM), septate junction assembly, apical-basal polarity and planar cell polarity genes (Hayashi and Dong, 2017). We find that expression of the secretory machinery genes, chitin production and modification genes, and known regulators of tube growth are tightly correlated (Fig. 4E; correlations of 0.943 and 0.944), with ‘Golgi mediated vesicle transport’ slightly preceding expression of tube size regulator genes, which, in turn, slightly precedes expression of genes affecting the chitin-based cuticle. Interestingly, whereas tracheal tube size control has focused on events affecting apical junctions and the apical ECM, the Llimargas group has discovered a role for the basement membrane (basal ECM) in tube size (Klussmann-Fricke et al., 2022). They demonstrated that loss of laminin genes or of the tracheal proteins that serve as receptors for the laminins (αPS3βPS integrin and Dystroglycan) results in over-elongated and mispositioned tracheal tubes. Given that the primary source of laminins is the plasmatocytes, this process must require careful temporal and spatial coordination between these cell populations, reflecting on the stages of organogenesis where tissue maturation becomes less autonomous and more dependent on other cell types.

Our data also captured the developmental trajectory of tracheal tip cells, which are a minor proportion of the tracheal cell population. Further characterization of the genes upregulated in this cell population should provide new insight into branching morphogenesis, a process that is crucial for the elaboration of many tissues across phyla.

### Germ cell analysis reveals two potential trajectories and suggests a role for widespread proteolysis in GC development

In contrast to the transcriptomic results from the SG and trachea, which largely confirm current models of organogenesis, the data from GCs highlight unexpected biological processes during gonad formation. Our analyses of GC transcriptomes reveal two potential trajectories (Fig. 5A,B). Based on expression of GC-specific determinant genes (*nos*, *gcl*, *pgc* and *vas*) and both male- and female-specific GC genes, we propose that one trajectory leads to the committed germline stem cell fate (the early-intermediate-late GC trajectory; Fig. 5A, Fig. S9A,B). We propose that the other GC clusters correspond to cells that do not become germline stem cells and that eventually become cystoblasts in females and spermatogonia in males, with ‘unknown 1’ including both male and female differentiating cells (based on their high expression of translation components) (Tables S9, S10) (Sanchez et al., 2016) and ‘unknown 2’ corresponding to the cells that will later form spermatogonia – based on expression levels of known spermatogonia genes, *CG6701* and *Pp2C1*, and diminished expression of *otu* and *ovo*, two oogenesis-specific genes (Fig. S9A,B).

Other than with the unknown 2 germ cell cluster, we did not find any additional evidence of separation of GCs based on sex in the stages that we were observing; this is in contrast to the findings from a recent report on scRNA-seq of 0-8 h (embryonic stages 1-12) GCs selected on the basis of expression of Vasa-GFP (for GCs) or Sxl-GFP expression (for sex) (Li et al., 2021). This group reported that as zygotic transcription commenced in the germline, two separate GC clusters emerged, one with relatively higher expression of X-linked to autosomal genes. Based on analysis of scRNAseq of the Sxl-GFP sorted embryos, the cluster with higher expression of X-linked genes corresponded to female GCs and the other to male GCs, indicating that germline dosage compensation has not been fully deployed. Our comparison of gene expression ratio between the unknown cluster cells (unknown 1 and unknown 2) and the main trajectory cells (early germ cells, intermediate germ cells 1, intermediate germ cells 2 and late germ cells) does not reveal any differences in expression of X-linked versus autosomal genes (Fig. S35A,B). This suggests that GC dosage compensation is fully activated by stage 10-16 and cannot be used to distinguish female versus male GCs at these stages. Furthermore, we directly examined the expression of marker genes listed as ‘female’ versus ‘male’ in their unsexed and sexed data files, and did not observe any notable correlation with any of our GC clusters (Figs S35C,D, S36A,B).

Finding multiple suspected distinct GC populations in our data suggests that GCs are receiving signals regarding their fates as they migrate and subsequently coalesce with the SGPs to form the gonads. This is in keeping with recent findings in flies and worms indicating that the final commitment to the germline stem cell fate is a sequential process that is partially induced (at least in flies) and may only be locked in once primordial GCs contact their supporting niche (reminiscent of mammalian GCs) (Cassani and Seydoux, 2022; Colonnetta et al., 2022; Hakes and Gavis, 2023). Thus, our discovery of a split trajectory within the GC population during stages 10-16, many hours after GCs were previously thought to be irreversibly committed, seems particularly relevant. Exploring the functions of the genes expressed in cell populations within each trajectory as well as the spatial and temporal localization of distinct markers for each cluster should enlighten future studies regarding when GCs become immortalized and commit to forming gametes.

Our GC transcriptomic data also revealed that the upregulation of genes encoding the proteosomal machinery in the developmental trajectory we propose leads to the formation of germline stem cells. Selective RNA degradation has been shown to be a crucial step in the specification and formation of germline stem cells (Cassani and Seydoux, 2022; Hakes and Gavis, 2023) and recent studies in worms also suggest a role for protein degradation: the E3-ubiquitin ligase GRIF-1 promotes the degradation of maternal proteins in PGCs and is required redundantly with Nanos for fertility (Lee et al., 2017; Oyewale and Eckmann, 2022). Our finding that ubiquitin-dependent and -independent proteasomal pathways are highly enriched in the germline stem-cell trajectory (intermediate and late GCs) in *Drosophila* is consistent with a conserved role for protein degradation in early GC development.

### Matrisome analysis confirms known sources for some components and clarifies sources of others

Our investigation of the organogenetic transcriptome included the embryonic matrisome as it is an essential substrate for cell morphogenesis, tissue architecture and organ formation (Klussmann-Fricke et al., 2022; Loganathan et al., 2016c; Walma and Yamada, 2020). Previous studies of the role of the *Drosophila* ECM in organogenesis have shown the plasmatocytes as the key source of the embryonic ECM (Matsubayashi et al., 2017). Our organogenetic transcriptome data confirms this model and extends the likely role of hemocytes (both plasmatocytes and crystal cells), along with yolk cells, as being the key embryonic source of insulin-like peptides. Indeed, *in situ* hybridizations recently carried out by the BDGP confirm *Ilp4* expression in plasmatocytes and crystal cells (Fig. S37A) and *Ilp6* expression in yolk (Fig. S37B). Surprisingly, we find that other major components of the embryonic ECM, the cuticle, Tweedle proteins and chitin-binding domain-containing proteins, are mainly produced by apodemes during early organogenetic stages, and by the esophagus, hypopharynx and lateral sensory neurons during late organogenetic stages. This contrasts with findings reported by Cattenoz et al. (2020) on the expression of these genes in plasmatocytes. Their findings were based on bulk seq of purified cells expressing UAS-DsRed under the control of *srp*(*hemo*)-Gal4. Our findings could be reconciled if the Gal4 driver used to isolate plasmatocytes (Brückner et al., 2004) is also expressed in other cell types.

### Concluding remarks

We note several important limitations and future improvements to our experimental framework. First, we recognize that, although our clusters captured all major organs known to exist in the embryo, there are still rare cell types within those organs missing from the dataset, such as salivary duct, proventriculus and somatic gonadal precursor cells. The missing cell types were either not captured or were absorbed in other cell clusters. Second, as shown in this study, not all SG genes were captured by scRNA-seq. This suggests that a high amount of dropout is still a problem where weakly or transiently expressed genes would not be captured (Chen et al., 2019). Third, with the rapid development of single-cell multi-omics technologies (Lee et al., 2020), characterizing multiple data modalities from a single-cell or nucleus, molecular changes beyond transcriptional changes during organogenesis will be possible (e.g. chromatin structure, methylation status and protein expression). Last, whereas pseudotime can provide a relative ordering of cells during development, the exact developmental stages of cells are unknown. Development of novel computational methods such as the one described by Calderon et al. (2022) could potentially help us characterize the developmental stages of single cells.

In summary, we have generated organogenetic transcriptome data and used these data for an extensive evaluation of their discovery and prediction characteristics. Our single cell transcriptome analyses not only reveal large numbers of genes that are differentially expressed in developing organs and in subsets of cells within those organs, but they have also uncovered unappreciated processes driving organogenesis, e.g. the potential role of plasmatocytes and yolk as sources of growth signals and the possible role of protein catabolism in GC programming. As more *Drosophila* embryonic single-cell datasets are released, it is crucial that studies compare their datasets with those of previously published studies for experimental conditions, harmonization of cell types, and cross-validation of findings and predictions. Accordingly, we have compared our data with those of the two closely related datasets currently available (Calderon et al., 2022; Seroka et al., 2022). Our results show that sequencing depth is a key parameter in the utility of available transcriptomic data, and underscore the need for cross-validation and comparison of emerging high-throughput data with archived datasets to enhance the informative power of single cell transcriptome methods for the study of development.

## MATERIALS AND METHODS

### Single cell RNA-seq data collection

Embryos were collected and aged on apple juice caps seeded with yeast paste. For stage 10-12 (early), embryos were collected at 25°C for 5 h and aged at 18°C for 12 h. For stage 13-16 (late), embryos were collected at room temperature for 7 h and aged at room temperature for 9 h. Embryos were harvested in collection baskets, soaked in 50% bleach for 3 min to remove the chorion, then rinsed thoroughly and visually inspected under a dissecting microscope to verify staging. Embryos were transferred to a Dounce homogenizer containing 1 ml ice-cold 1×PBS. Embryos were disrupted using 20 strokes (up and down) of the loose pestle. The material was transferred to a 1.5 ml microfuge tube and pelleted by a 3 min spin in a tabletop centrifuge at 1500 RCF (***g***) at 4°C. Supernatant was removed and material was resuspended in 1 ml fresh ice-cold 1×PBS, then passaged 20 times through a 22-gauge needle, then four times through a 23-gauge needle. The cell suspension was then passed through a 40 μm cell strainer into a 1.5 ml microcentrifuge tube. Cells were pelleted by a 3 min spin in a tabletop centrifuge set at 3300 RCF at 4°C, then resuspended in 100 μl ice-cold 1×PBS and immediately subjected to single cell isolation and transcriptome sequencing library preparation with the 10X Genomics Chromium, using the 3′ Single Cell V3 chemistry. Two independent wild-type (OregonR) samples were prepared and processed for each timed collection.

### Antibody staining

Embryo fixation and immunohistochemistry were performed as described previously (Reuter and Scott, 1990). Fkh (Fox et al., 2013) and Vasa (Santa Cruz Biotechnology, sc-30210, RRID:AB_793874) rabbit polyclonal antisera were used at 1:2000 and 1:1000, respectively. αGasp (Developmental Studies Hybridoma Bank, 2A12; AB_528492; https://dshb.biology.uiowa.edu) was used at 1:100.

### *In situ* hybridization

*In situ* hybridization was performed as described previously (Lehmann and Tautz, 1994). Antisense RNA *trh* probes were generated from the cDNA clones isolated by Isaac and Andrew (1996).

### Single cell RNA-seq processing

Sequence reads were processed with CellRanger (Zheng et al., 2017) version 6.1.2 using *Drosophila* genome version 6.33 (downloaded from FlyBase; Gramates et al., 2022) resulting in gene counts for each cell. The filtered count matrix from each sample was loaded and subjected to standard computational pre-processing and cell-level quality control (Luecken and Theis, 2019) before integration of time point replicates. All the computational analyses were performed using R (version 4.1.2) and Seurat (Hao et al., 2021) (version 4.2.1). Cells with high mitochondrial transcript counts proportion (>25%) were excluded from further analysis. Standard Seurat workflow was performed to cluster the cells. Cells in clusters with fewer than 50 marker genes that were above average log2-fold change of 0.25 were removed as they likely represent empty droplets. After these empty samples were removed, DoubletFinder (McGinnis et al., 2019) (version 2.0.3) was used to remove doublets in each sample. After the poor quality, empty and doublet cells were filtered out from each sample, the two Seurat datasets from the same stages were merged. The merged dataset was log-normalized using standard Seurat function, and the top 2000 variable genes were used for scaling and downstream principal component analysis (PCA). Principal components (PCs) with eigenvalues greater than the upper bound of Marchenko-Pastur distribution of a random matrix with the same dimensions, found by using R package RMTstat (version 0.3.1) (Johnstone et al., 2022), were retained as significant PCs for downstream analysis. Harmony (Korsunsky et al., 2019a) (version 0.1.0) was used to embed cells from each timepoint replicate in a shared, dimensionally reduced space to reduce the impact of batch effect on cell clustering. Batch-corrected PCs from Harmony were used as the basis for constructing the nearest-neighbor graph, cell clustering Louvain clustering (Blondel et al., 2008) and 2D embedding with UMAP (McInnes et al., 2018 preprint).

To find the marker genes of cell clusters, differential gene expression analysis was performed with Seurat's ‘FindAllMarkers()’ function using a likelihood-ratio test to identify preferentially expressed genes for each cluster. The results of ‘FindAllMarkers()’ function are in Table S2. An adjusted *P*-value threshold of <0.05 was applied to identify significantly expressed genes for each cluster. We selected 4-6 diagnostic marker genes for each cluster from among the genes with the top 50 highest log-fold change, and matched them to the cell type annotations curated from the BDGP database (Table S3).

### Cell type gene set enrichment analysis

Logfold-changes of genes for each cell type relative to rest of the embryonic cells were computed using R package Presto (Korsunsky et al., 2019b preprint) (version 1.0.0). The genes with less than 10% expression in both cell type of interest and the rest of the embryonic cells were removed as they are most likely to be lowly expressed genes. The logfold changes for each gene were used as stats to be inputted into ‘fgsea()’ function from R package fgsea (Korotkevich et al., 2016 preprint) (version 1.20.0) to perform preranked gene set enrichment analysis. Gene sets fewer than 10 or more than 500 genes were not included in the GSEA analysis. Otherwise, standard parameters were used to perform GSEA with the number of permutations set to 1000 for the estimation of *P*-value. The gene set library GO_Biological_Process_2018 was downloaded from modEnrichR (Kuleshov et al., 2019).

### Pseudotime analysis

Raw count matrix from the cell type of interest (SG, trachea and GC) were first parsed out and Monocle3 (Cao et al., 2019) (version 1.3.1) was used to perform all the downstream analysis, including dimensional reduction, clustering and pseudotime inference. No batch correction was used.

Cross-correlation was used to elucidate the temporal associations between two gene set expressions along the pseudotime ordering for the main trajectory of trachea cells (tip cells were removed from downstream analysis). First, the expression of leading-edge genes (from Table S8) in biological processed gene sets [Golgi vesicle transport (GO:0048193), regulation of tube size, open tracheal system (GO:0035151) and chitin-based cuticle development (GO:0040003)] were averaged, smoothed using base R function ‘ksmooth()’ across pseudotime, and scaled using base R function ‘scale()’. The purpose of the previous step is to average the dynamics of all the genes in the biological processes to get average expression dynamics of the biological processes. Cross-correlations between averaged and smoothened expression of gene sets were calculated using base R function ‘ccf()’.

### SingleCellNet classifications

pySingleCellNet (Tan et al., 2021 preprint) was installed from the Github repo (https://github.com/CahanLab/PySingleCellNet) (commit fb440247f6f372367b8cca015c40b82f9f388573) and Python (version 3.9.12) was used for performing cell type classification. To perform classification on our data, the raw count matrix and the accompanying harmonized cell type labels from Seroka et al. (2022) and Calderon et al. (2022) scRNA-seq data (with the unknown cell type removed) were used as training data. SingleCellNet automatically generates random profiles and uses them as training for the random or unknown categories, which are completely different from the unknown cell types from the scRNA-seq data. For more information on the workflow of SingleCellNet, see Tan and Cahan (2019). Each cell was annotated as the cell type with the highest classification score. The proportions of classified cell types in each of the cell types annotated by us were calculated to compare the agreement between the cell typing of Seroka et al. and Calderon et al. with ours. If the majority of cells in the cell type annotated by us are classified as the same cell type, then there is a high agreement in terms of cell typing. In total, two classifiers (for stage 10-12 and stage 13-16) were created for scRNA-seq data of both Seroka et al. and Calderon et al. for comparing cell typing agreement.

To use our data to identify the rare cell types in the other datasets, we trained two classifiers using our stage 10-12 and stage 13-16 data, respectively, with the unknown cell types removed. The cell type with the highest classification score was annotated as the classified cell type for each cell. The original UMAP embeddings from Seroka et al. and Calderon et al. were used for plotting the location of classified rare cell type clusters.

### Embryonic cell line comparisons

The bulk expression profiles (in RPKM) for the embryonic cell lines and adult tissues were downloaded from FlyBase (Gramates et al., 2022). The bulk RNA-seq data for embryonic cell lines were originally from Brown et al. (2014) and the bulk RNA-seq data for adult tissues were originally from Leader et al. (2018). The top 60 most preferentially expressed genes were selected for each cell type in both stage 10-12 and 13-16 scRNA-seq, which led to total of 1604 unique marker genes. The cell type reference expression matrix was compiled through averaging single cell expressions in that cell type for the 1604 unique marker genes. Spearman correlations between query bulk RNA-seq and reference expression matrix were calculated to identify the most transcriptionally similar cell type for each cell line.

## Supplementary Material

10.1242/develop.202097_sup1 Supplementary information

Table S1. Experimental details of embryo collections.

Table S2. Differentially expressed genes for individual clusters relative to rest of the embryonic cells in stage 10-12 and stage 13-16 embryos.

Table S3. Selected markers genes for individual clusters in stage 10-12 and stage 13-16 embryos that are cross-referenced with BDGP for cell type identification.

Table S4. GSEA results for SG, trachea and germ cells relative to rest of the embryonic cells in stage 10-12 and stage 13-16 embryos.

Table S5. Differentially expressed genes for SG sub-populations (early and late SG cells) relative to all other SG cells.

Table S6. GSEA results for different SG sub-populations (early and late SG cells) relative to all other SG cells.

Table S7. Differentially expressed genes for trachea sub-populations (early tracheal cells, intermediate tracheal cells, late tracheal cells, and tracheal tip cells) relative to all other tracheal cells.

Table S8. GSEA results for different trachea sub-populations (early tracheal cells, intermediate tracheal cells, late tracheal cells, and tracheal tip cells) relative to all other tracheal cells.

Table S9. Differentially expressed genes for germ cells sub-populations (early germ cells, intermediate germ cells 1, intermediate germ cells 2, late germ cells, unknown 1 and unknown 2) r lative to all other germ cells.

Table S10. GSEA results for germ cell sub-populations (early germ cells, intermediate germ cells 1, intermediate germ cells 2, late germ cells, unknown 1 and unknown 2) relative to all other germ cells.

Table S11. The average expression and cell percent expression of all matrisome genes shown in Figure 6A, S10A, S12 for different cell types in stage 10-12 embryos.

Table S12. The average expression and cell percent expression of all matrisome genes shown in Figure 6B, S10B, S13 for different cell types in stage 13-16 embryos.

Table S13. Cell type harmonization for Seroka et al, Calderon et al and this study.
