# Supplementary material for: Organogenetic transcriptomes of the Drosophila embryo at single cell resolution
Source: Development. 2024 Jan 19;151(2):dev202097. doi: 10.1242/dev.202097 (PMC10820837; doi:10.1242/dev.202097)
Supplement: Supplementary information [file develop-151-202097-s1.pdf]

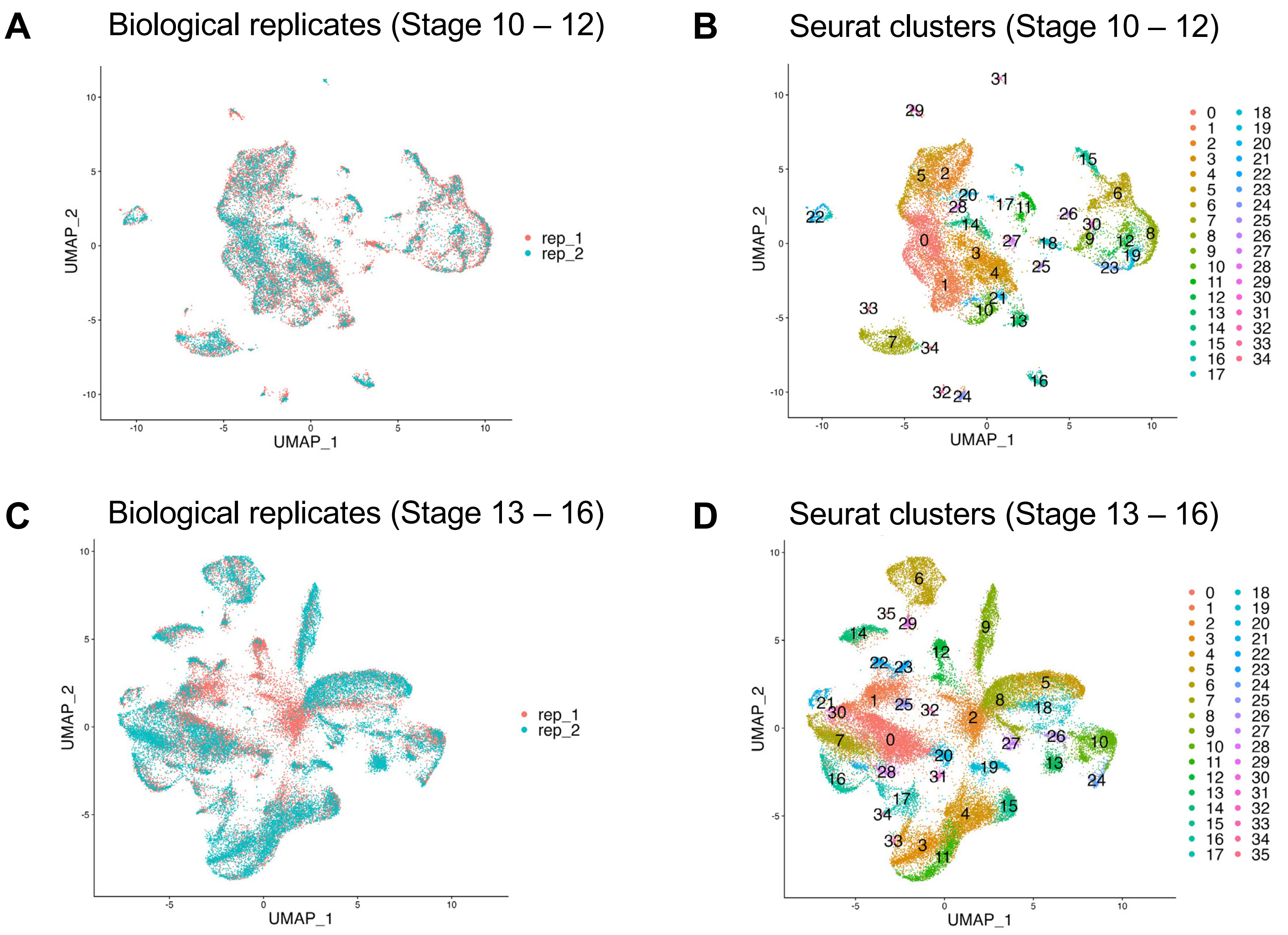

E Cluster groupings (Stage 10 – 12)

Stage 10 - 12 Cell Clusters - Assignments	Stage 10 - 12 Grouped Cell Types
Cluster 0 -- Epidermis 1	Epidermis
Cluster 1 -- Epidermis 2	Epidermis
Cluster 3 -- Epidermis 3	Epidermis
Cluster 4-- Epidermis 4	Epidermis
Cluster 2 -- CNS 1	CNS
Cluster 5 -- CNS 2	CNS
Cluster 6-- Somatic muscle 1	Somatic muscle
Cluster 8 -- Somatic muscle 2	Somatic muscle
Cluster 12 -- Somatic muscle 3	Somatic muscle
Cluster 19 -- Somatic muscle 4	Somatic muscle
Cluster 23 -- Somatic muscles 5	Somatic muscle
Cluster 17 -- Plasmatocytes 1	Plasmatocytes
Cluster 22 -- Plasmatocytes 2	Plasmatocytes
Cluster 24 --Germ cells 1	Germ cells
Cluster 32 -- Germ cells 2	Germ cells
Cluster 20 -- Glia	Glia
Cluster 14 -- Sensory neurons	Sensory neurons
Cluster 7 -- Gut endoderm	Gut endoderm
Cluster 9 -- Circular visceral mesoderm	Circular visceral mesoderm
Cluster 10 --Trachea	Trachea
Cluster 11 --Yolk	Yolk
Cluster 13 --Anterior and posterior gut primordium	Anterior and posterior gut primordium
Cluster 15 -- Fat Body	Fat Body
Cluster 16 -- Amnioserosa	Amnioserosa
Cluster 18 -- Pharyngeal muscle	Pharyngeal muscle
Cluster 21 -- Hindgut	Hindgut
Cluster 25 -- Apodemes	Apodemes
Cluster 26 -- Longitudinal visceral mesoderm	Longitudinal visceral mesoderm
Cluster 27 -- Salivary gland	Salivary gland
Cluster 28 -- Midline Glia	Midline Glia
Cluster 29 -- Unknown (CNS?)	Unknown (CNS?)
Cluster 30 -- Hindgut muscle	Hindgut muscle
Cluster 31 -- Crystal cells	Crystal cells
Cluster 33 -- Malphighian tubules	Malphighian tubules
Cluster 34 -- Posterior midgut primordium	Posterior midgut primordium

F Cluster groupings (Stage 13 - 16)

Stage 13 - 16 Cell Clusters - Assignments	Stage 13 -16 Grouped Cell Types
Cluster 0 -- Dorsal epidermis	Epidermis
Cluster 1 --Ventral epidermis	Epidermis
Cluster 3 -- CNS 1	CNS
Cluster 4 -- CNS 2	CNS
Cluster 11 -- CNS 3	CNS
Cluster 15 -- CNS 4	CNS
Cluster 33 -- CNS 5	CNS
Cluster 5 -- Somatic muscle 1	Somatic muscle
Cluster 8 -- Somatic muscle 2	Somatic muscle
Cluster 18 -- Somatic muscle 3	Somatic muscle
Cluster 14 -- Plasmatocytes	Plasmatocytes
Cluster 12 -- Germ cells	Germ cells
Cluster 19 -- Glia 1	Glia
Cluster 31 -- Glia 2	Glia
Cluster 17 -- Head sensory neurons	Sensory neurons
Cluster 34 -- Lateral sensory neurons	Sensory neurons
Cluster 2 -- Unknown	Unknown
Cluster 6-- Gut endoderm	Gut endoderm
Cluster 7 -- Hypopharynx	Hypopharynx
Cluster 9 -- Fat body	Fat body
Cluster 10 --Circular visceral mesoderm	Circular visceral mesoderm
Cluster 13 -- Pharyngeal muscle	Pharyngeal muscle
Cluster 16 --Trachea	Trachea
Cluster 20 -- Optic lobe	Optic lobe
Cluster 21 -- Hindgut	Hindgut
Cluster 22 -- Yolk	Yolk
Cluster 23 -- Amnioserosa	amnioserosa
Cluster 24 -- Longitudinal visceral mesoderm	Longitudinal visceral mesoderm
Cluster 25 -- Salivary gland	Salivary gland
Cluster 26 -- Hindgut muscle	Hindgut muscle
Cluster 27 -- Dorsal vessel	Dorsal vessel
Cluster 28 -- Epipharynx	Epipharynx
Cluster 29 -- Malpighian tubules	Malpighian tubules
Cluster 30 -- Esophagus	Esophagus
Cluster 32 -- Oenocytes	Oenocytes
Cluster 35 -- Garland cells	Garland cells

Fig. S1. UMAPs showing the (A) batch information and (B) Seurat clusters for stage 10-12 embryos. UMAPs showing the (C) batch information and (D) Seurat clusters for stage 13-16 embryos. Tables that map Seurat clusters to cell types for (E) stage 10-12 embryos and (F) stage 13-16 embryos.

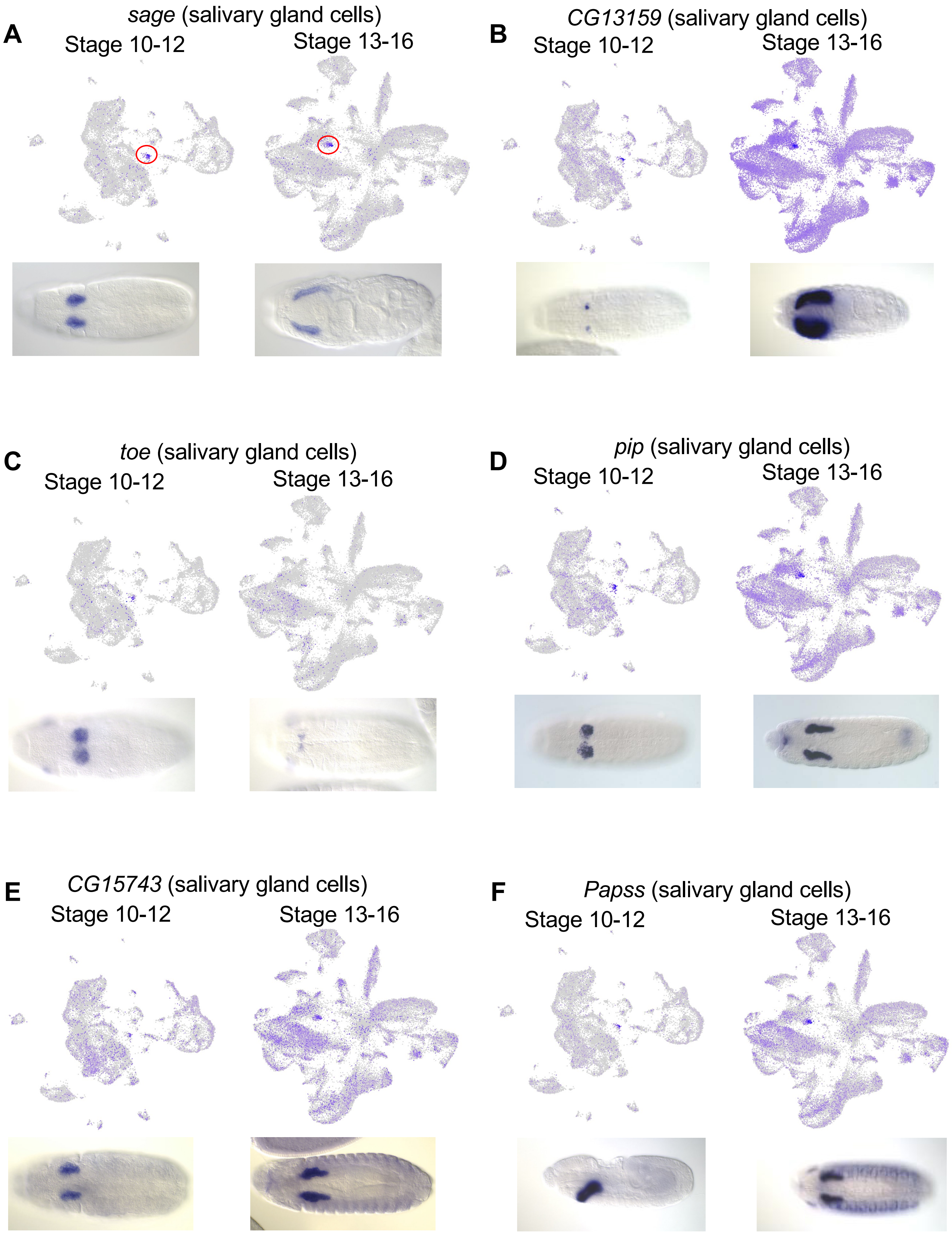

Fig. S2. UMAPs with six salivary gland marker genes' expression ((A) *sage*, (B) *CG13159*, (C) *toe*, (D) *pip*, (E) *CG15743* and (F) *Papss*) overlayed in both stage 10-12 and stage 13-16 embryos. Underneath the UMAPs are in situs from BDGP of the six salivary gland marker genes in stage 10-12 and stage 13-16 embryos. The red circles indicate the approximate UMAP locations of salivary gland cells for stage 10-12 and stage 13-16 embryos.

Development • Supplementary information

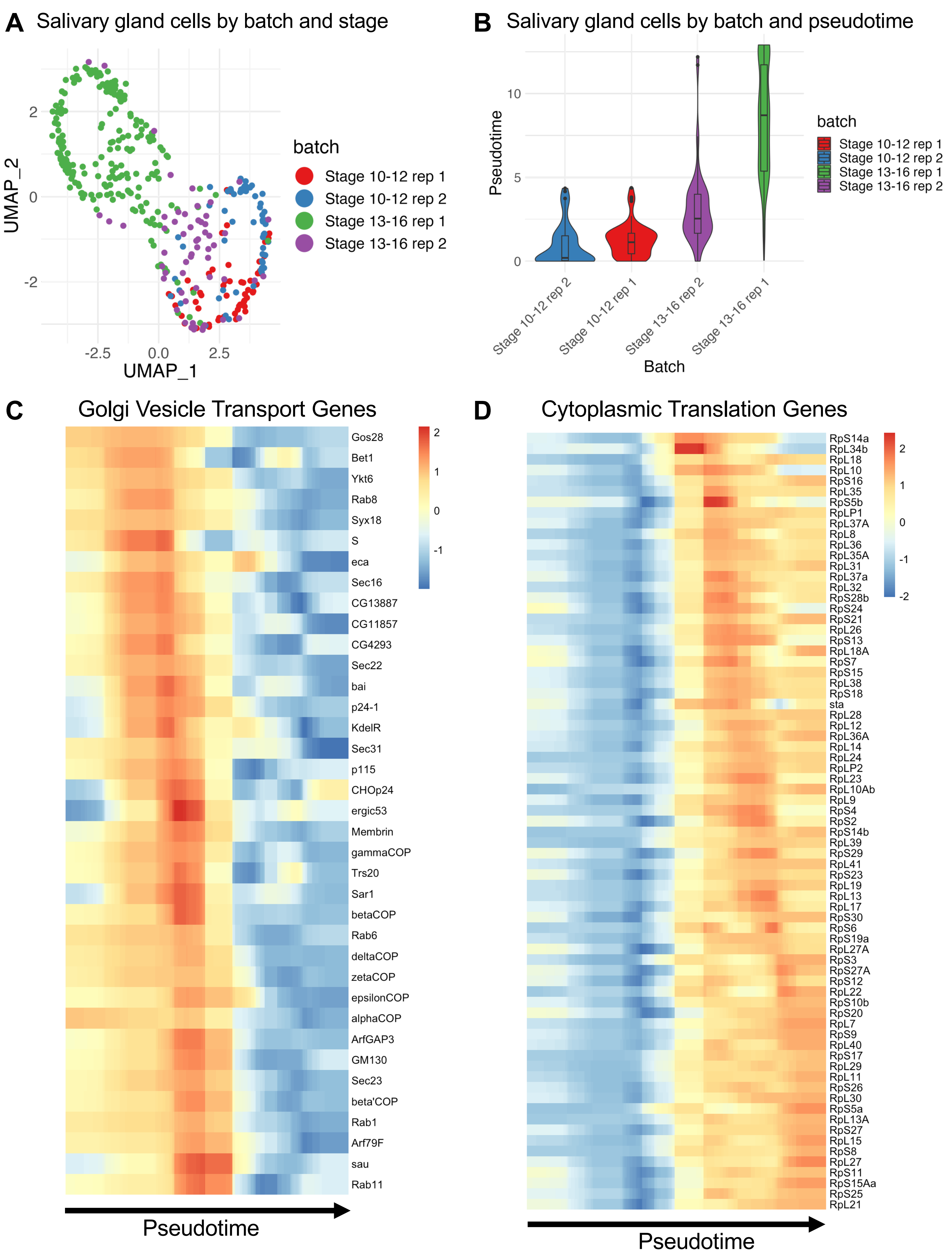

Fig. S3. (A) UMAP showing the batch information for SG cells. (B) Violin plot showing the distribution of pseudotime across different batches for SG cells. Scaled and smoothed expression of the leading edge genes in (C) Golgi Vesicle Transport gene set and (D) Cytoplasmic Translation gene set for SG cells across pseudotime.

Development • Supplementary information

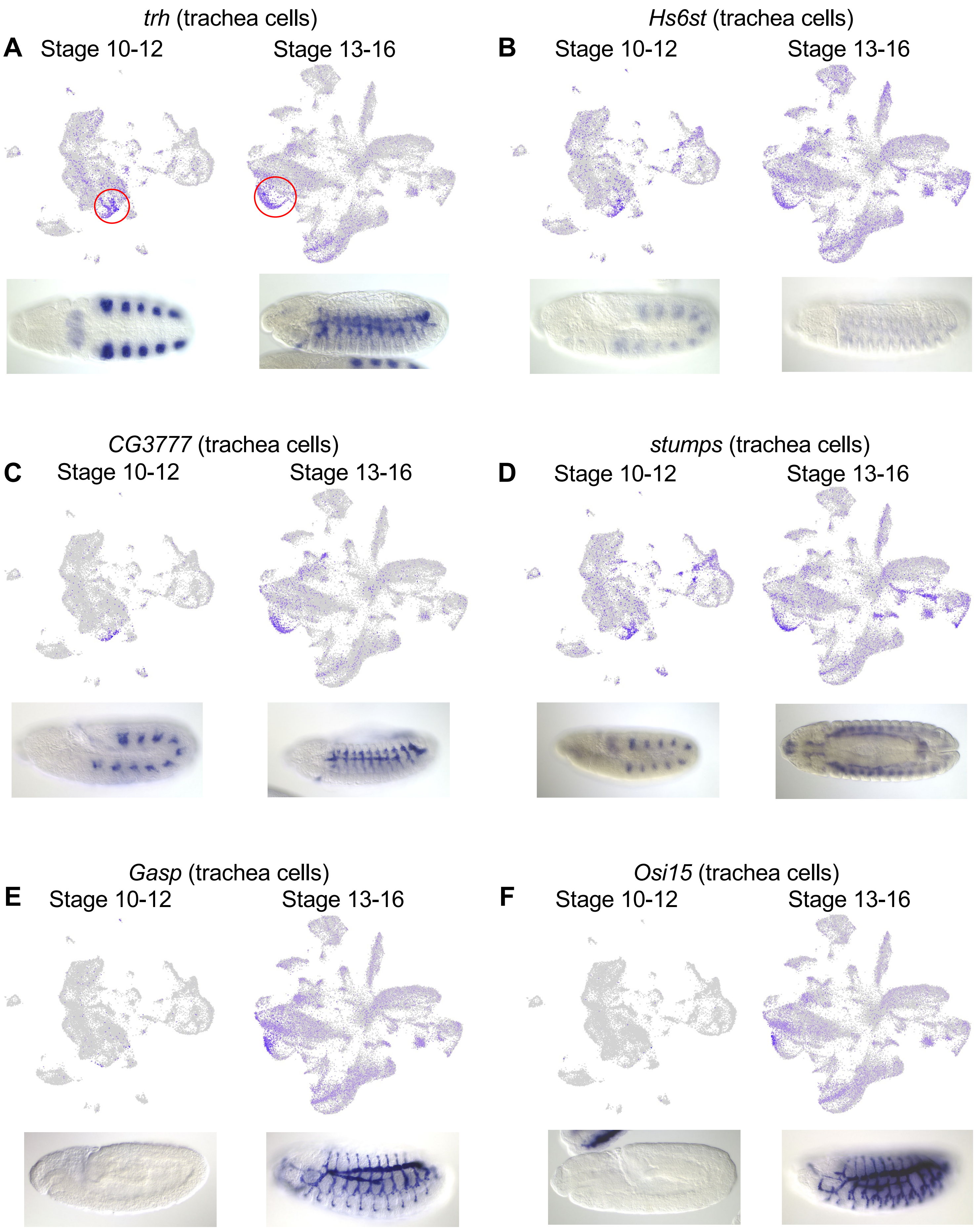

Fig. S4. UMAPs with six tracheal marker genes' expression ((A) *trh*, (B) *Hs6st*, (C) *CG3777*, (D) *stumps*, (E) *Gasp*, (F) *Osi15*) overlayed in both stage 10-12 and stage 13-16 embryos. Underneath the UMAPs are in situs from BDGP of the six tracheal marker genes in stage 10-12 and stage 13-16 embryos. The red circles indicate the approximate UMAP locations of tracheal cells for stage 10-12 and stage 13-16 embryos.

Development • Supplementary information

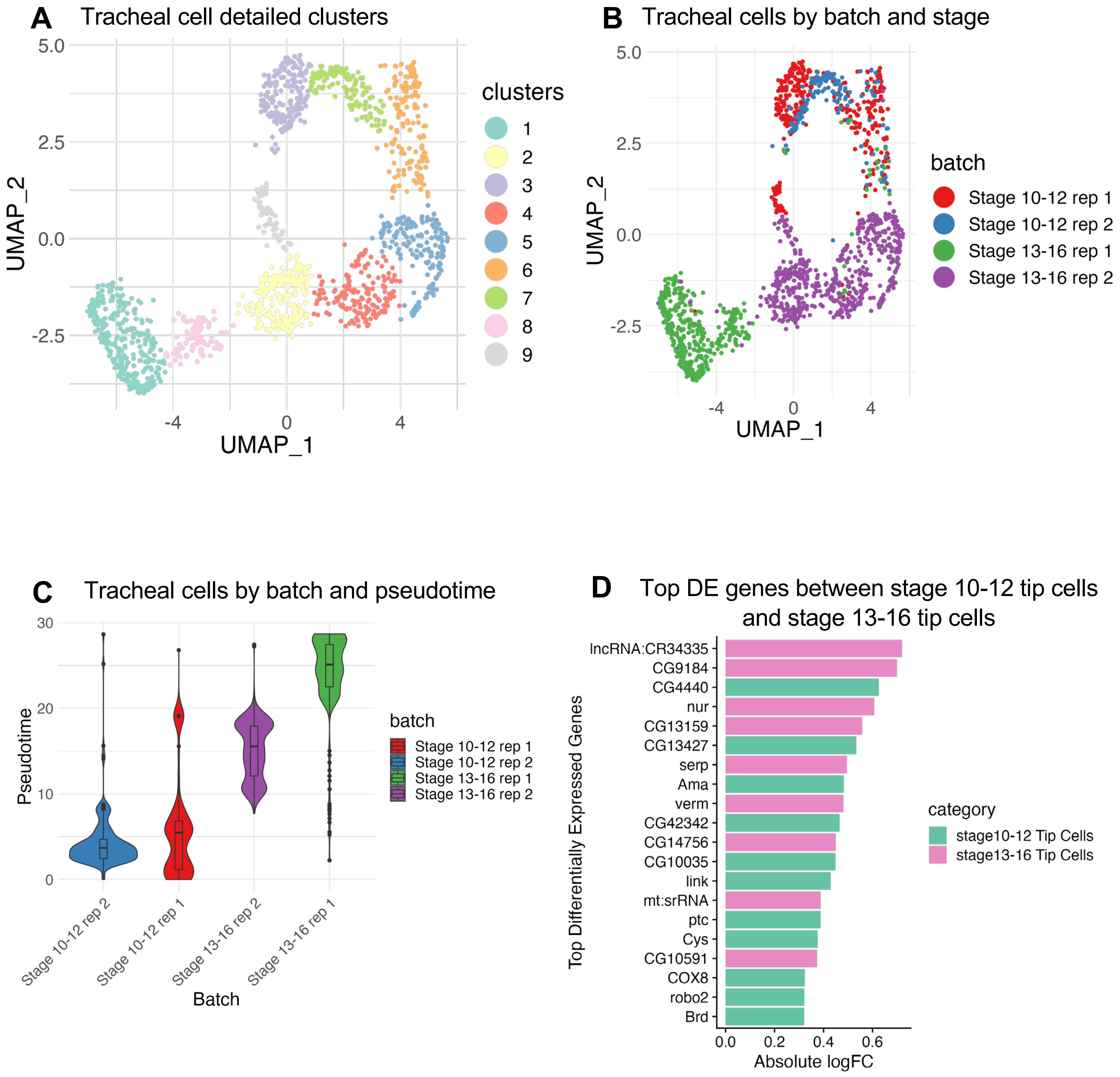

Fig. S5. UMAPs showing the (A) clusters and (B) batch information for tracheal cells. (C) Violin plot showing the distribution of pseudotime across different batches for tracheal cells. (D) Bar plot showing the top 20 most differentially expressed genes between stage 10-12 tip cells and stage 13-16 tip cells. The green color of the bar represents genes that are expressed higher in stage 10-12 tip cells. The purple color of the bar represents genes that are expressed higher in stage 13-16 tip cells.

Development • Supplementary information

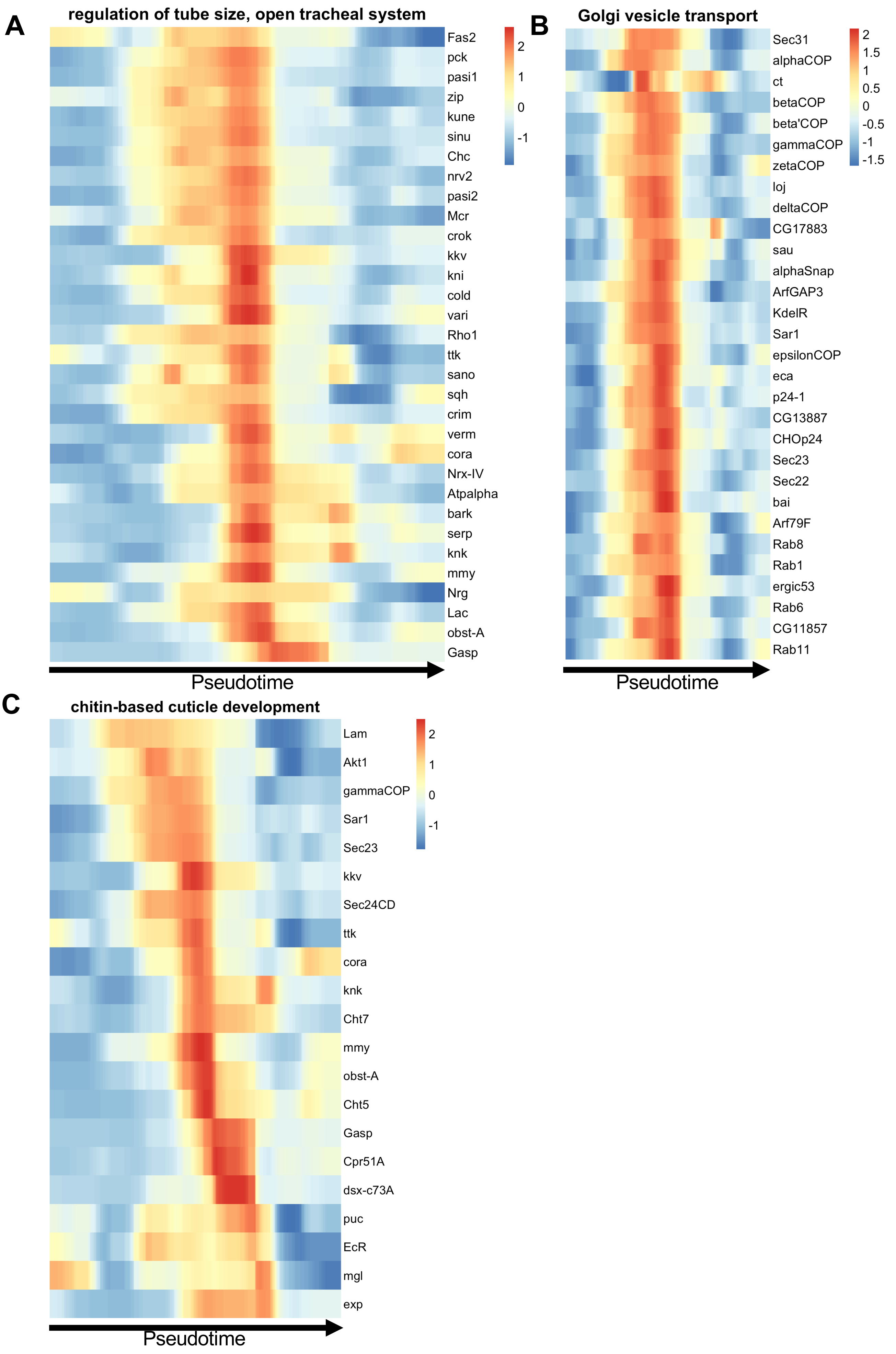

Fig. S6. Scaled and smoothed expression of leading edge genes in (A) regulation of tube size, open tracheal system, (B) Golgi vesicle transport and (C) chitin-based cuticle development gene sets for tracheal cells (excluding the tracheal tip cells) across pseudotime.

Development • Supplementary information

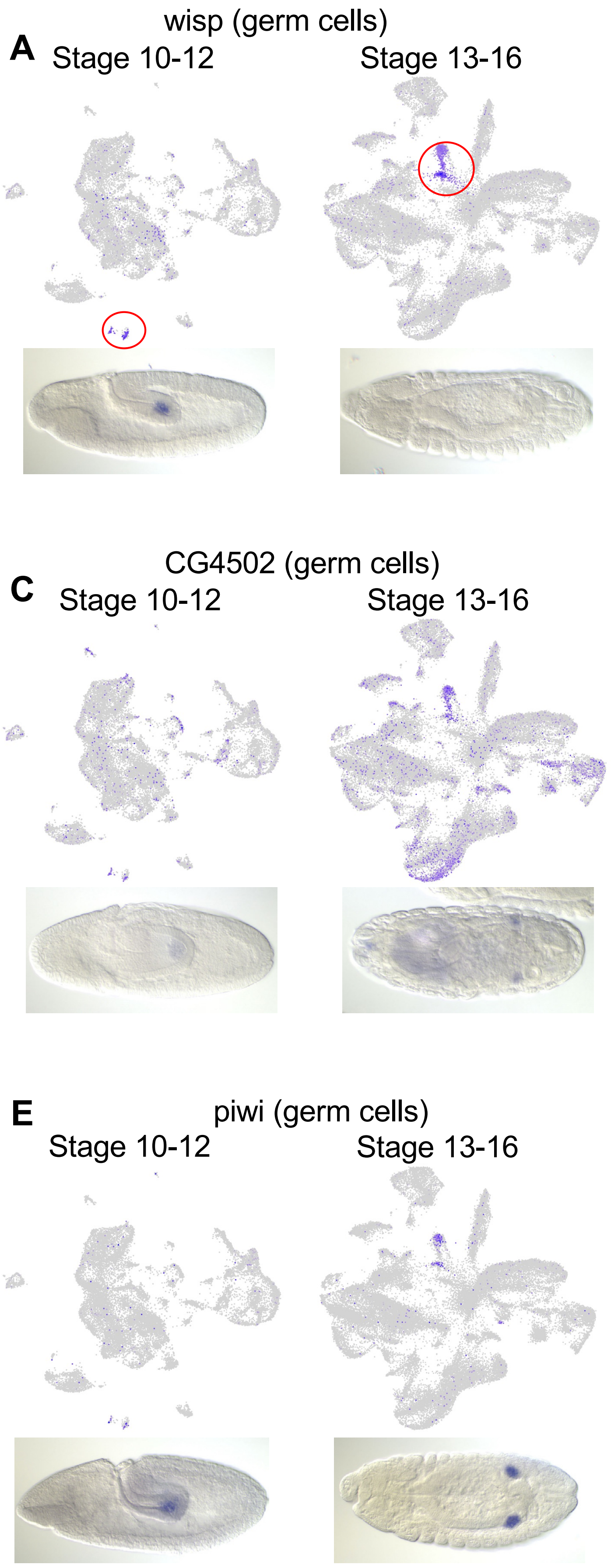

Fig. S7. UMAPs with six germ cell marker genes' expression ((A) *wisp*, (B) *stai*, (C) *CG4502*, (D) *pgc*, (E) *piwi*, (F) *Hsp26*) overlayed in both stage 10-12 and stage 13-16 embryos. Note that this is the only late-stage embryo image available at BDGP for *wisp*. Underneath the UMAPs are in-situs from BDGP of the six germ cell marker genes in stage 10-12 and stage 13-16 embryos. The red circles indicate the approximate UMAP locations of germ cells for stage 10-12 and stage 13-16 embryos.

Development • Supplementary information

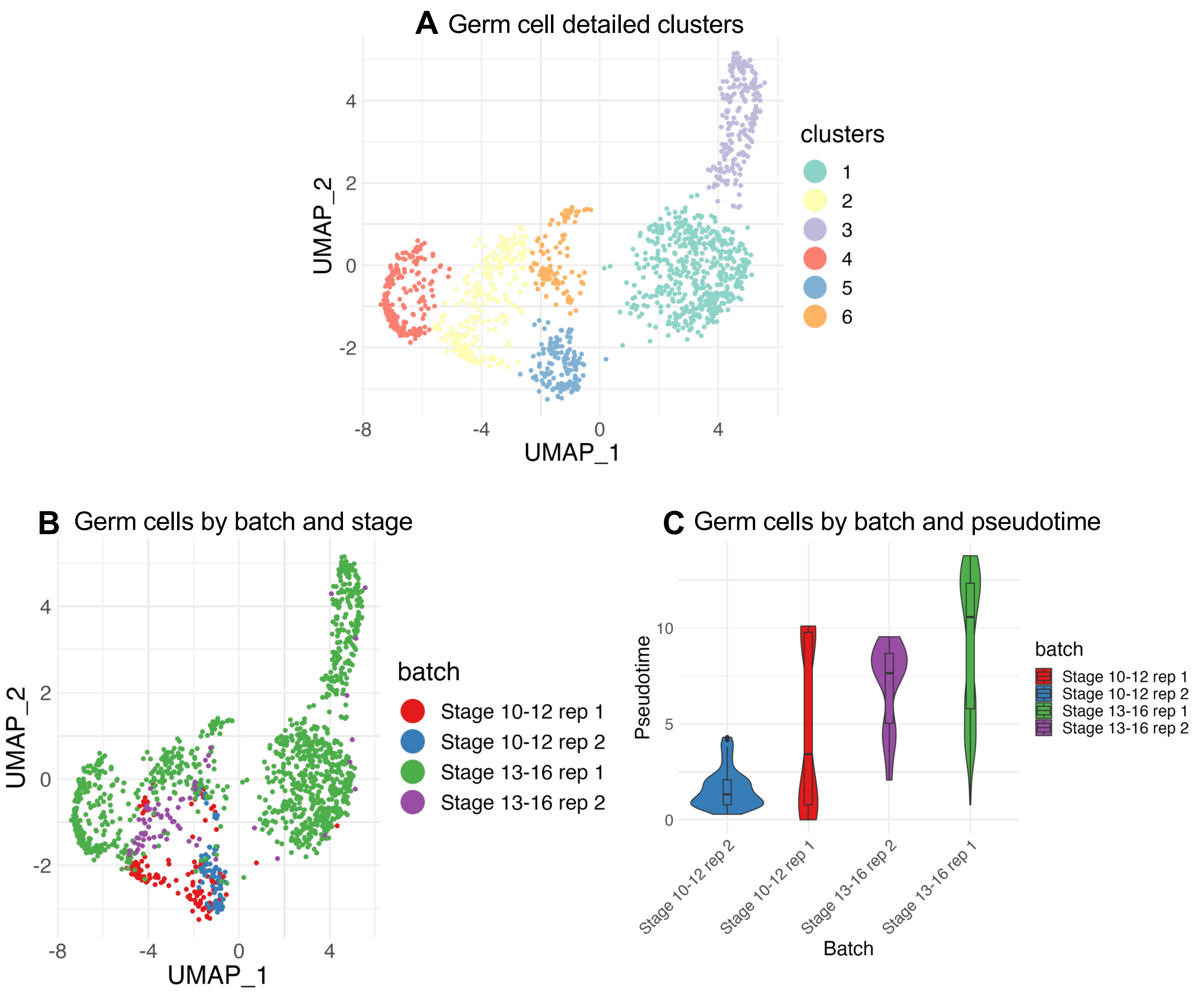

Fig. S8. UMAPs showing the (A) clusters and (B) batch information for germ cells. (C) Violin plot showing the distribution of pseudotime for the main trajectory (Early Germ Cells, Interm. Germ Cells 1, Interm. Germ Cells 2, Late Germ Cells) across different batches for germ cells.

Development • Supplementary information

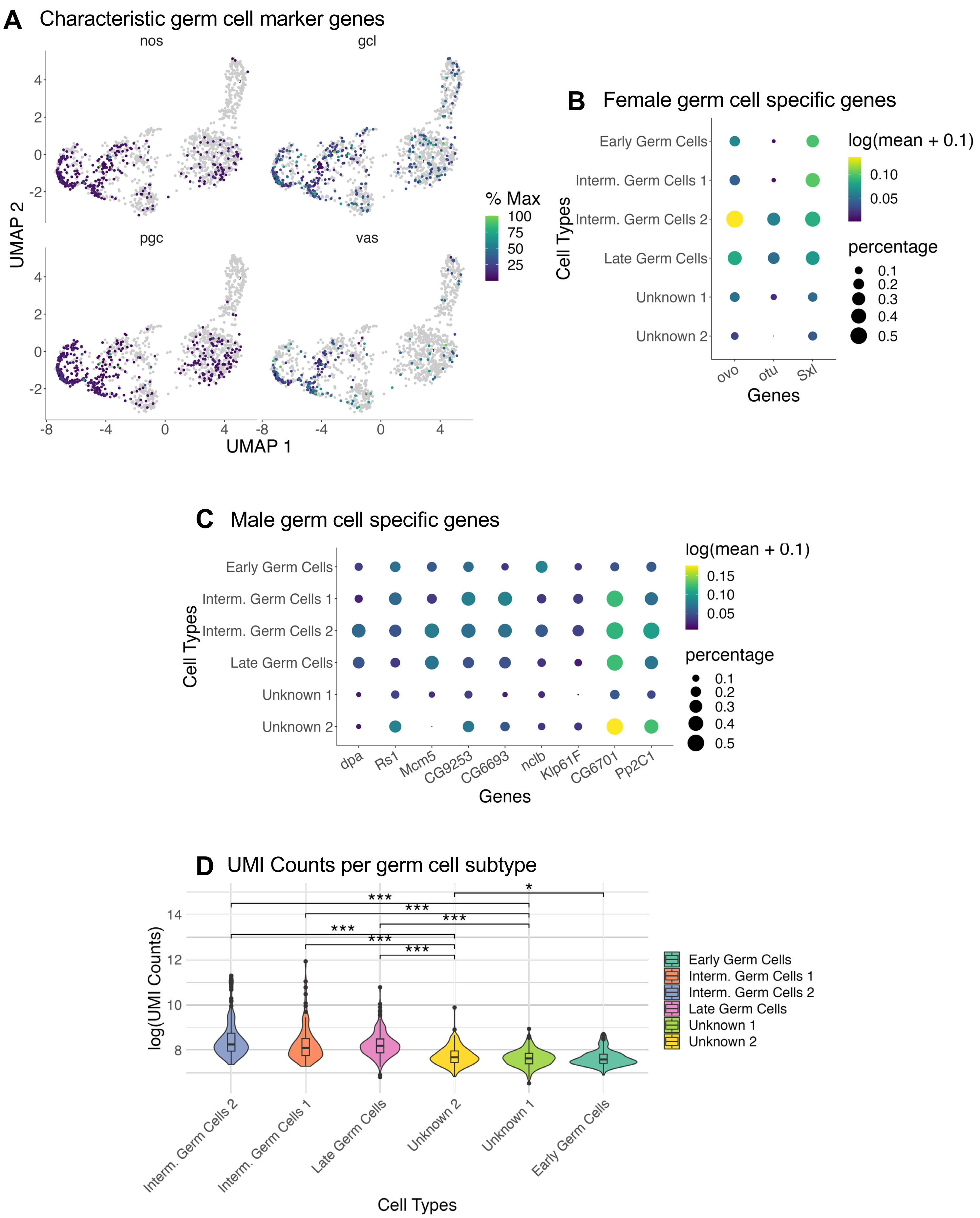

Fig. S9. (A) UMAPs of germ cells with characteristic germ cell marker genes overlayed. Dot plots of characteristic marker genes for (B) female GC specificity and (C) male GC specificity. The size of the dot represents the percentage of cells in the GC sub-population in which the gene is detected and the color of the dot represents the mean expression of the gene in the GC sub-population. (D) Violin plot of log UMI counts across different GC sub-populations.

Development • Supplementary information

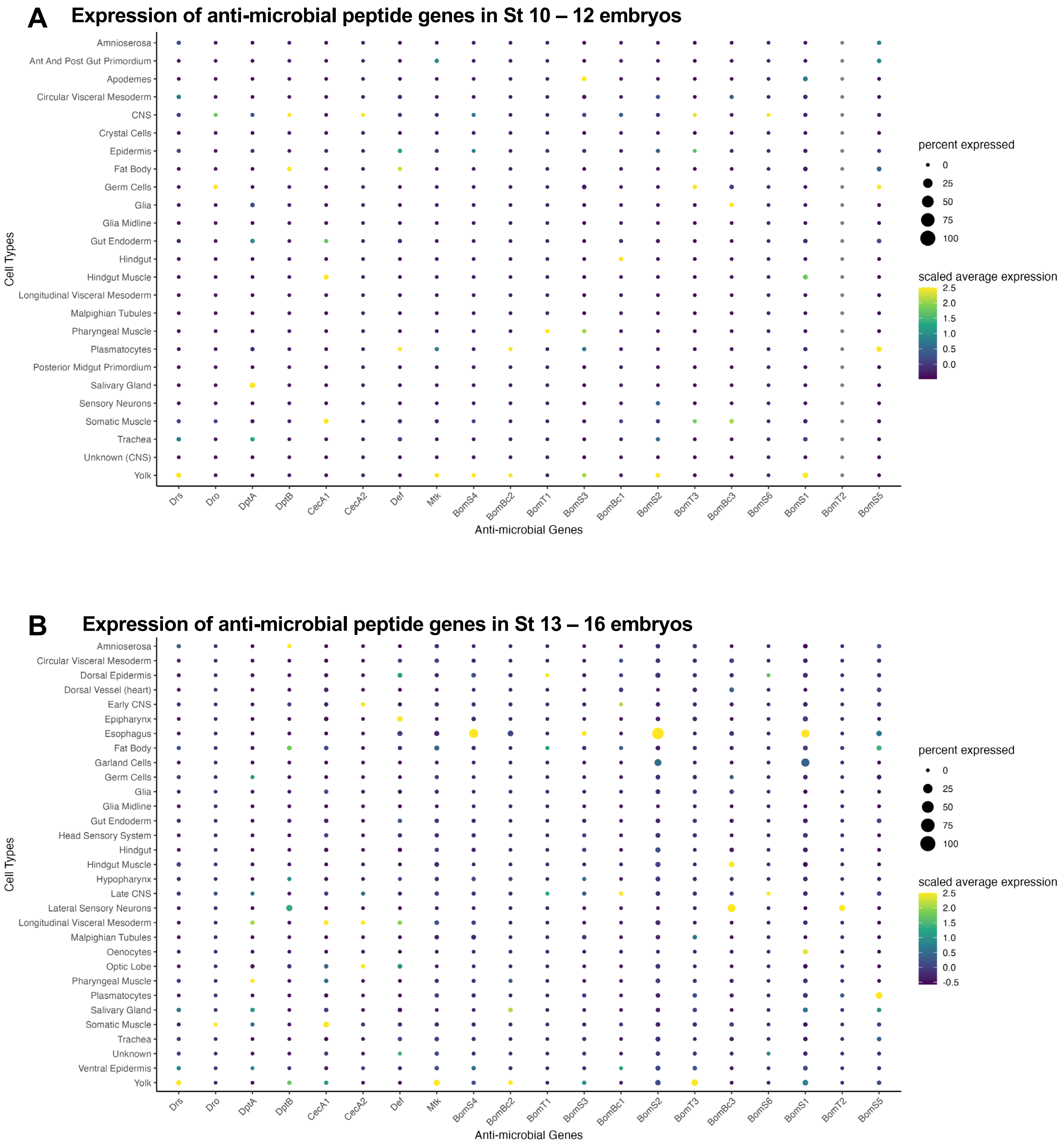

Fig. S10. Dot plots of anti-microbial genes expressions across cell types in (A) stage 10-12 embryos and (B) stage 13-16 embryos. The size of the dot represents the percentage of cells in the embryonic cell type in which the gene is detected and the color of the dot represents the mean expression of the gene in the embryonic cell type.

Development • Supplementary information

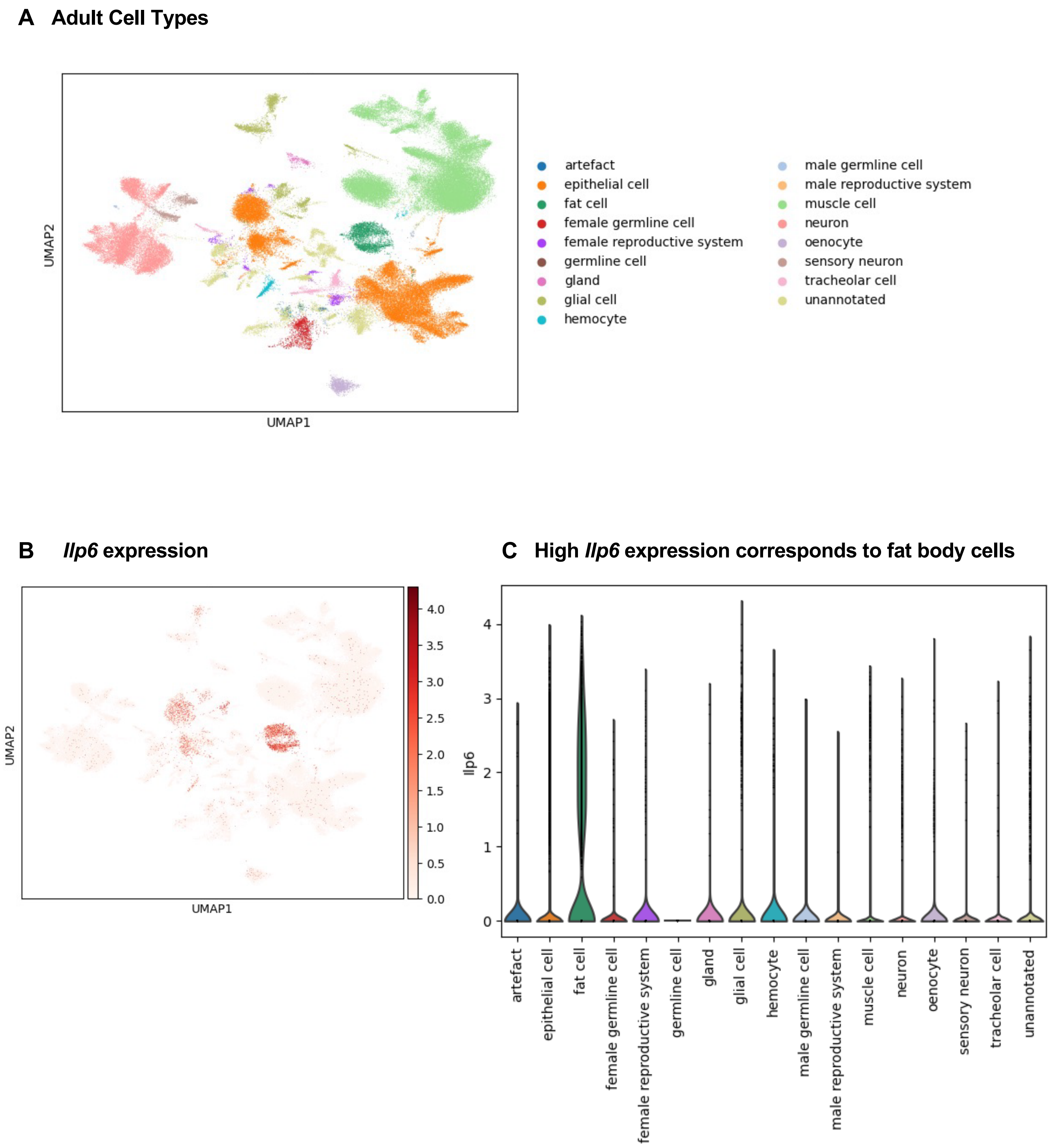

Fig. S11. *Ilp6* expression patterns in adult fruit fly scRNA-seq from Fly Cell Atlas. (A) UMAP showing adult fruit fly cell types. (B) UMAP showing *Ilp6* expression. (C) Violin plot of *Ilp6* expression across different adult fruit fly cell types.

Development • Supplementary information

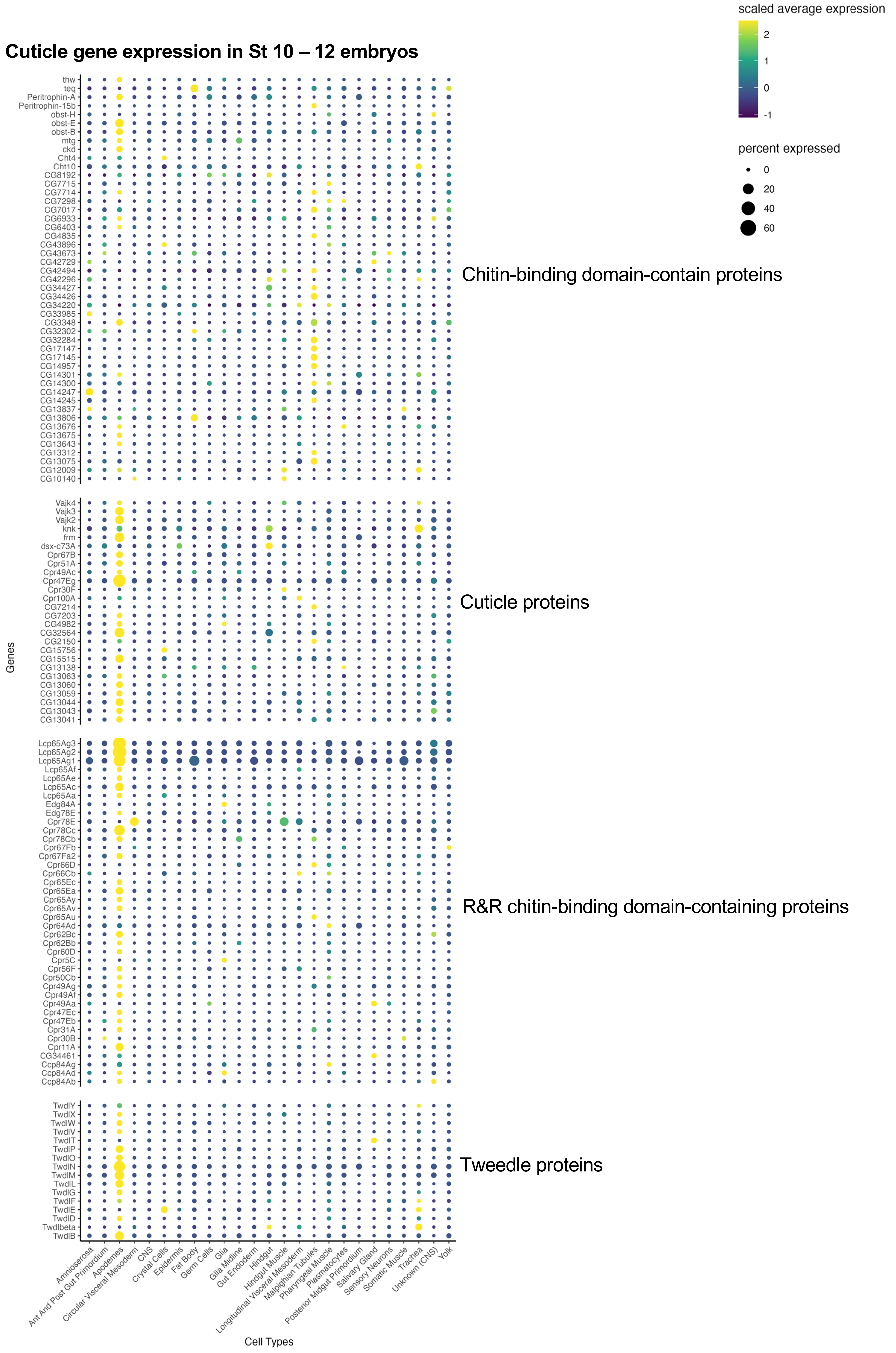

Fig. S12. Dot plot of cuticle genes across cell types in stage 10-12 embryos. Genes that have percentage of expressing cells less than 1% in all the cell types were removed as lowly expressed genes. The size of the dot represents the percentage of cells in the embryonic cell type in which the gene is detected and the color of the dot represents the mean expression of the gene in the embryonic cell type.

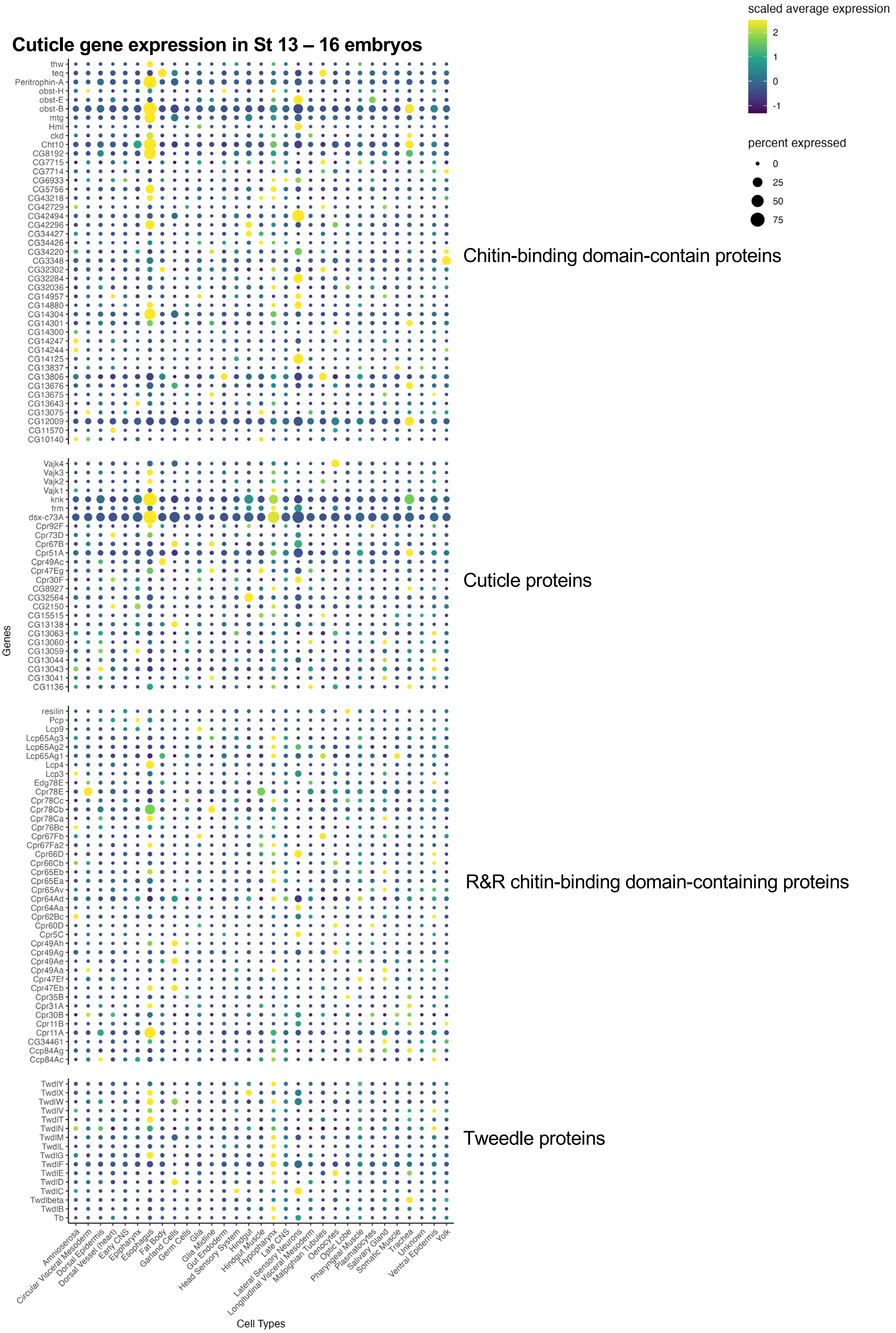

Fig. S13. Dot plot of cuticle genes across cell types in stage 13-16 embryos. Genes that have percentage of expressing cells less than 1% in all of the cell types are removed as lowly expressed genes. The size of the dot represents the percentage of cells in the embryonic cell type in which the gene is detected and the color of the dot represents the mean expression of the gene in the embryonic cell type.

Development • Supplementary information

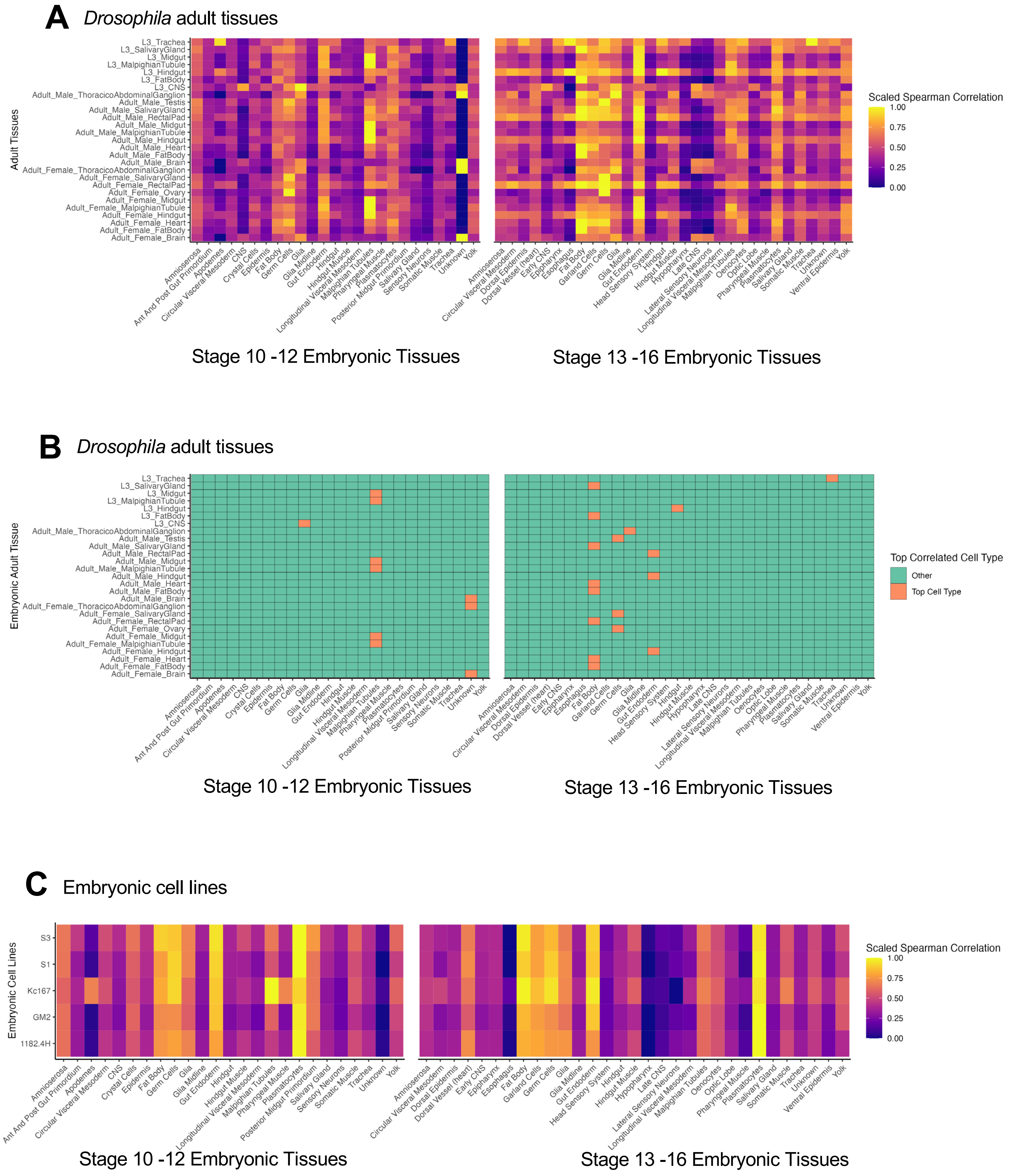

Fig. S14. Heatmaps of (A) scaled Spearman correlation between adult *Drosophila* tissue expression profiles and average pseudo-bulk expression profiles from single-cell type in terms of the 1604 tissue marker genes and (B) top correlated single-cell cell type clusters for adult *Drosophila* tissues. (C) Heatmap of scaled Spearman correlation between *Drosophila* embryonic cell lines' expression profiles and average pseudo-bulk expression profiles from single-cell cell type clusters in terms of the 1604 tissue marker genes.

Development • Supplementary information

A Comparisons between the scRNA-seq cell types in this study and in other published work, St 10 -12

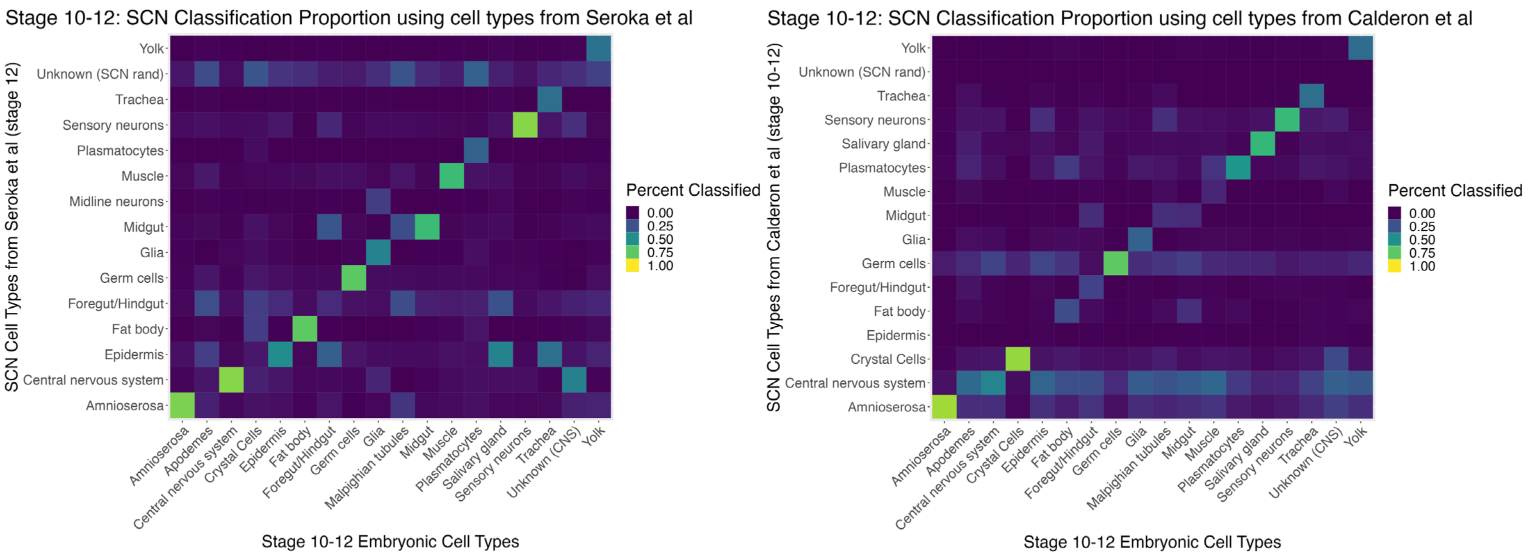

B Comparisons between the scRNA-seq cell types in this study and in other published work, St 13-16

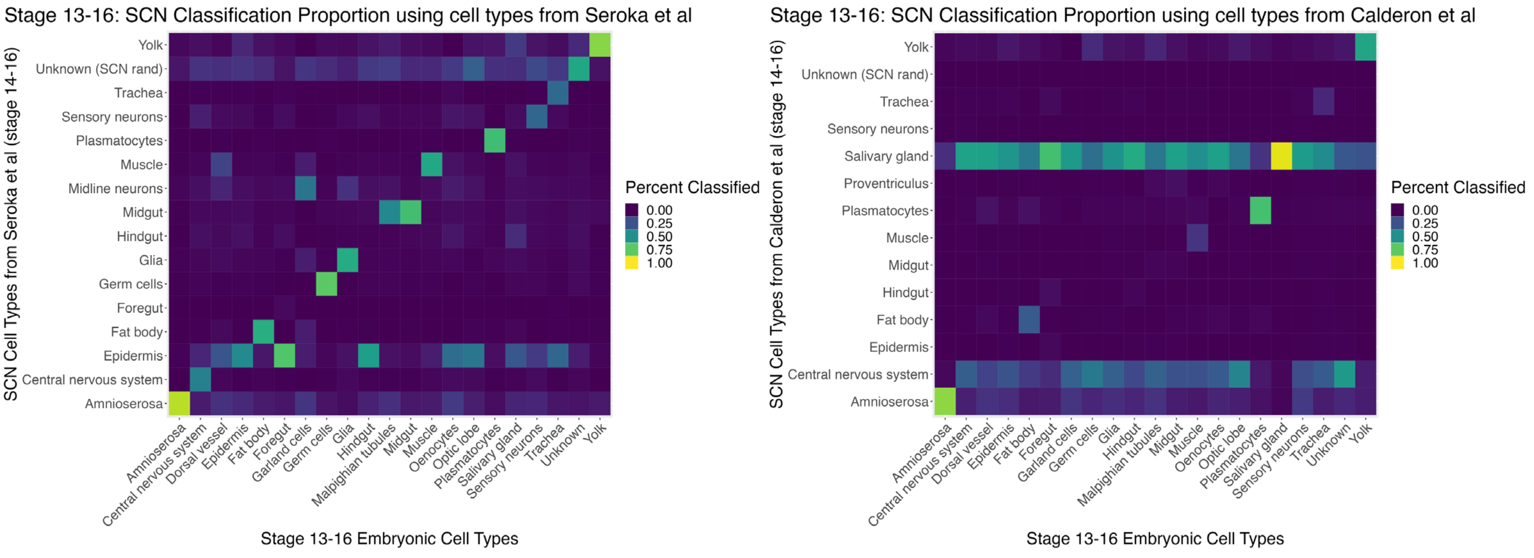

Fig. S15. Classification results of SingleCellNet classifier trained on single-cell data from Seroka et al and Calderon et al and applied on the single-cell data in this study. Heatmaps showing the percentage of stage 10-12 cells in our embryonic cell-types that are classified as harmonized cell types from (A) Seroka et al (on the left) and Calderon et al (on the right). Heatmaps showing the percentage of stage 13-16 cells in our embryonic cell-types that are classified as harmonized cell types from (B) Seroka et al (on the left) and Calderon et al (on the right).

Development • Supplementary information

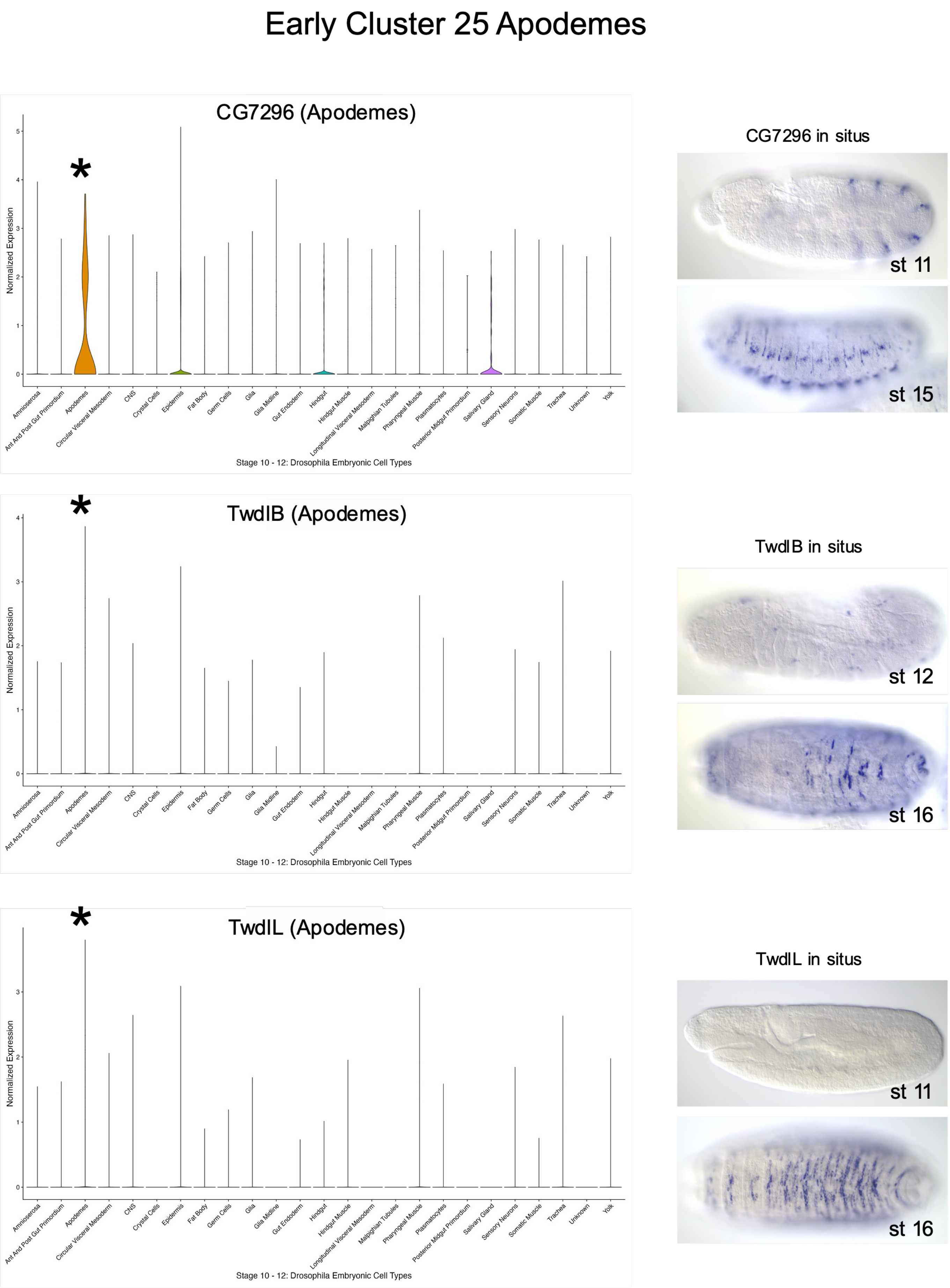

Fig. S16. Violin plots (on the left) and in-situ images from BDGP (on the right) of early apodemes marker genes: *CG7296* (top), *TwdlB* (middle) and *TwdlL* (bottom).

Development • Supplementary information

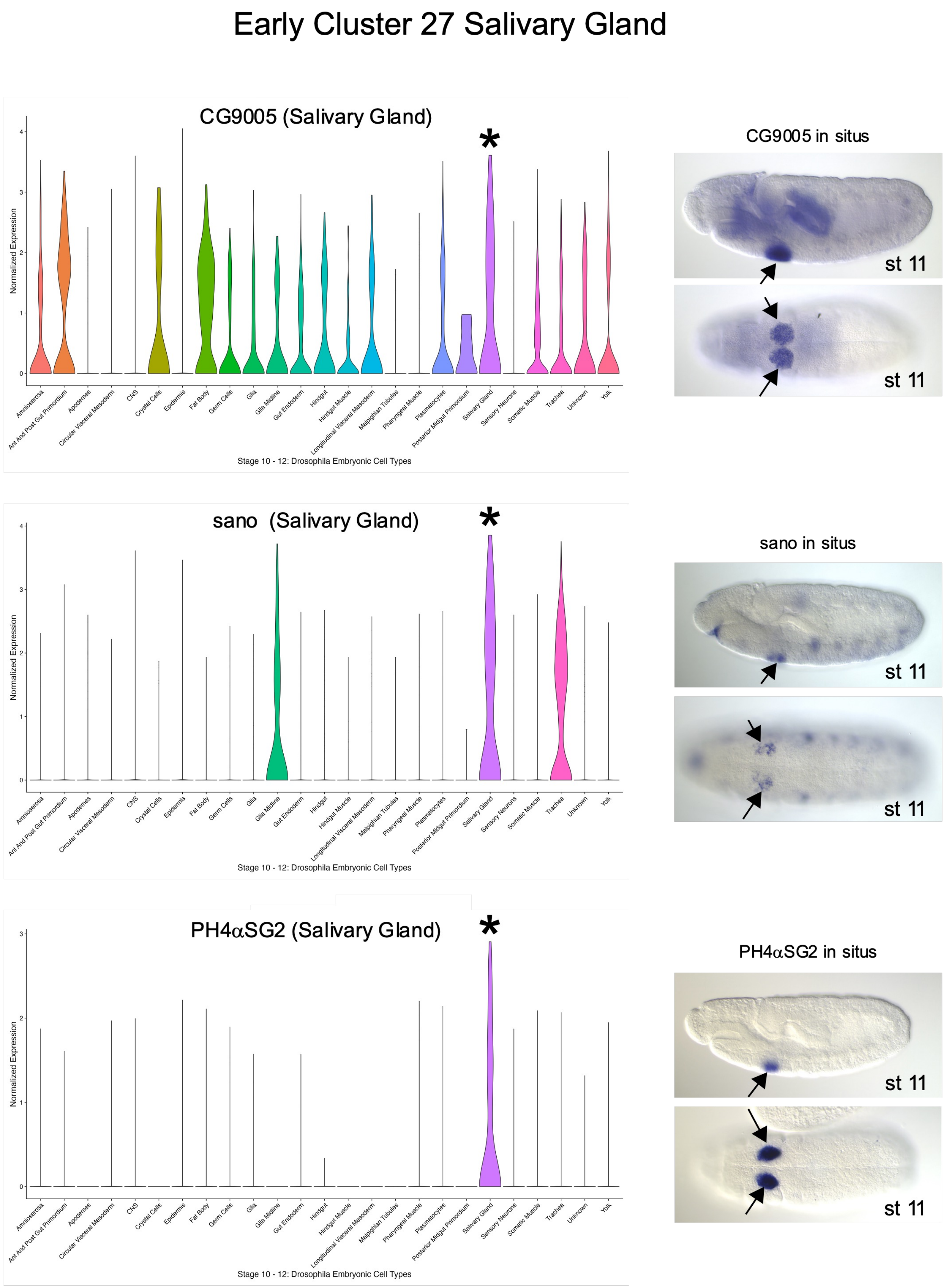

Fig. S17. Violin plots (on the left) and in-situs images from BDGP (on the right) of early SG marker genes: *CG9005* (top), *sano* (middle) and *PH4αSG2* (bottom).

Development • Supplementary information

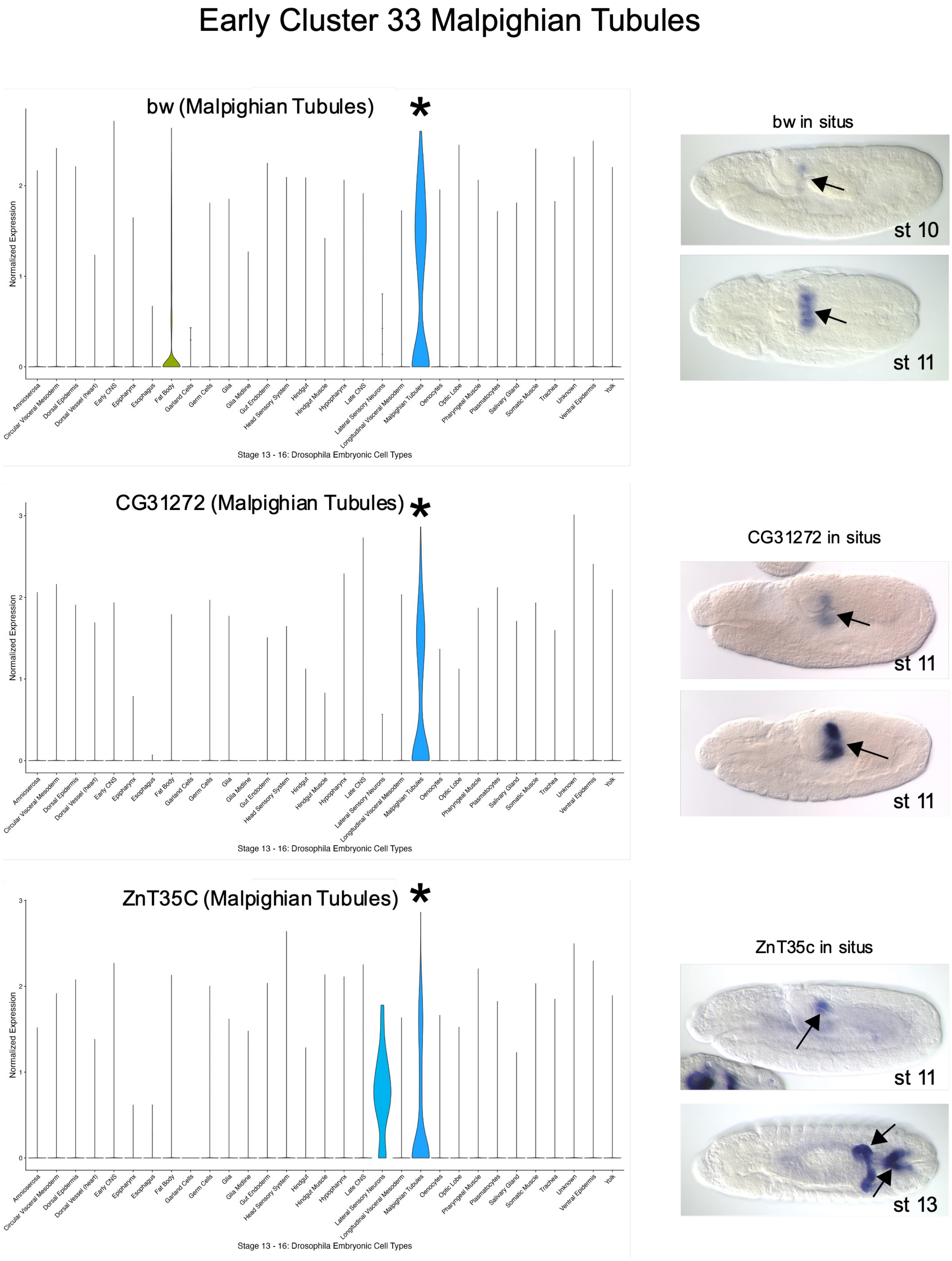

Fig. S18. Violin plots (on the left) and in-situ images from BDGP (on the right) of early Malpighian tubules marker genes: *bw* (top), *CG31272* (middle) and *ZnT35C* (bottom).

Development • Supplementary information

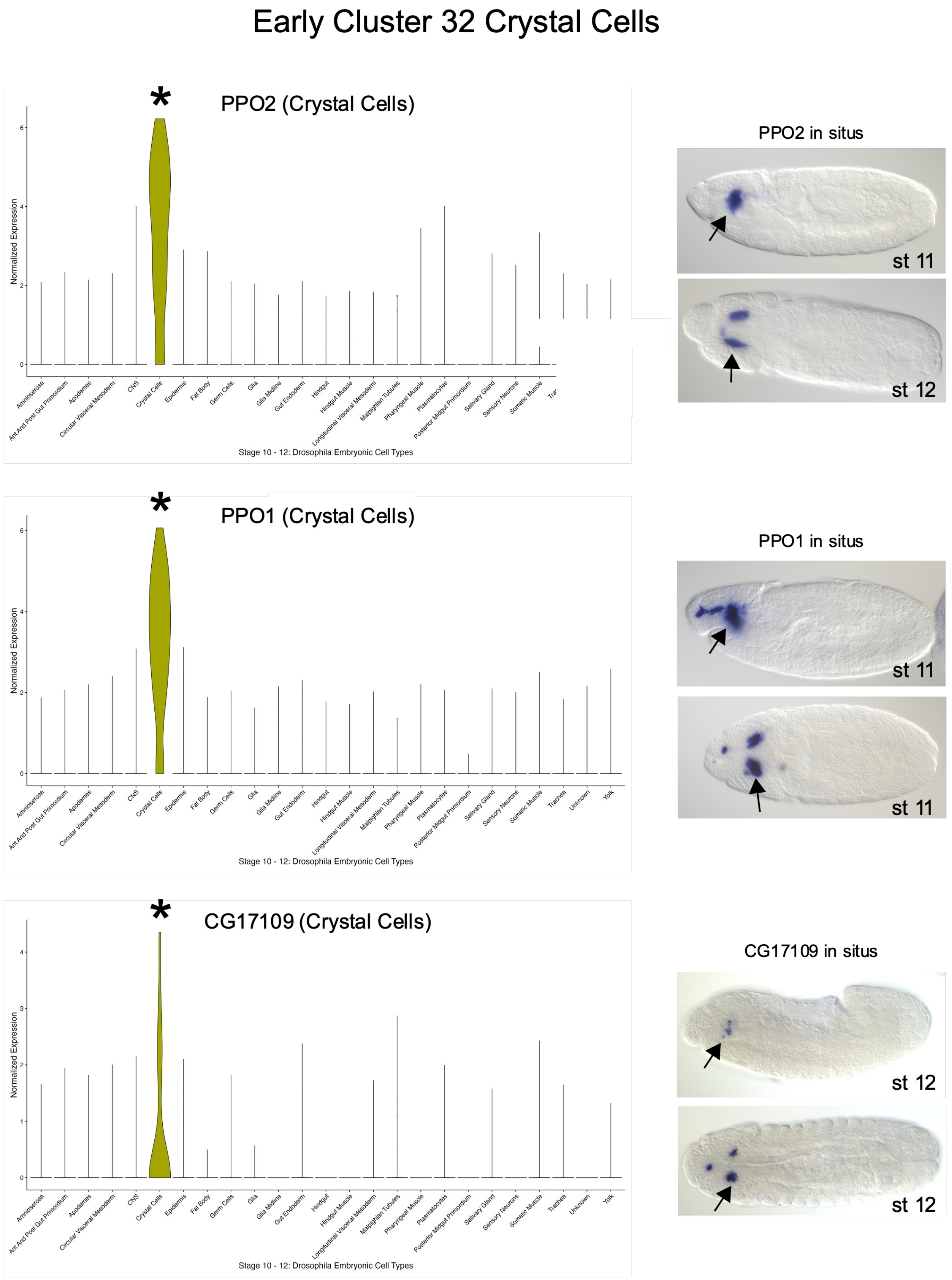

Fig. S19. Violin plots (on the left) and in-situs images from BDGP (on the right) of early crystal cells marker genes: *PPO2* (top), *PPO1* (middle) and *CG17109* (bottom).

Development • Supplementary information

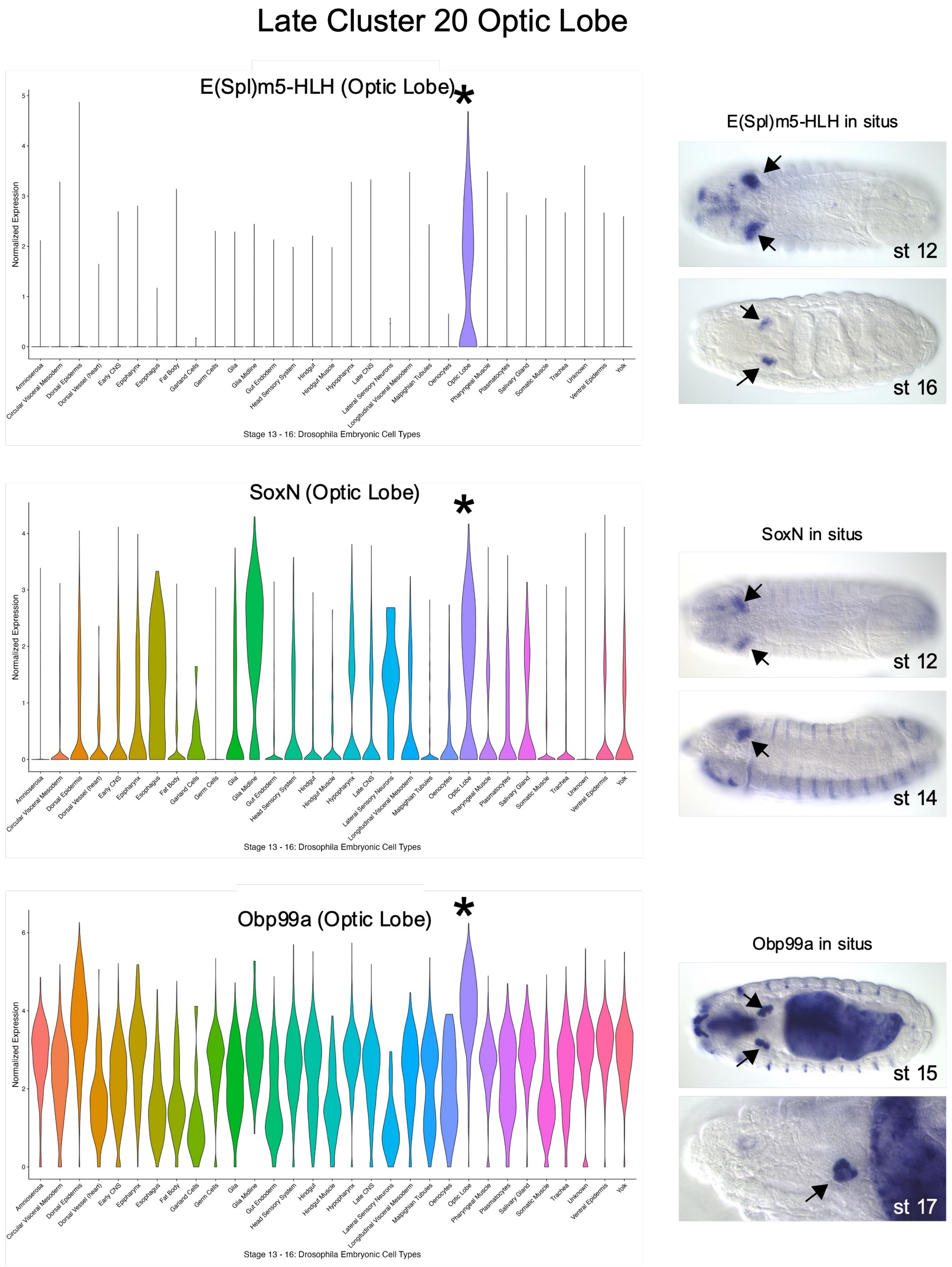

Fig. S20. Violin plots (on the left) and in-situs images from BDGP (on the right) of late optic lobe marker genes: *E(Spl)m5-HLH* (top), *SoxN* (middle) and *Obp99a* (bottom).

Development • Supplementary information

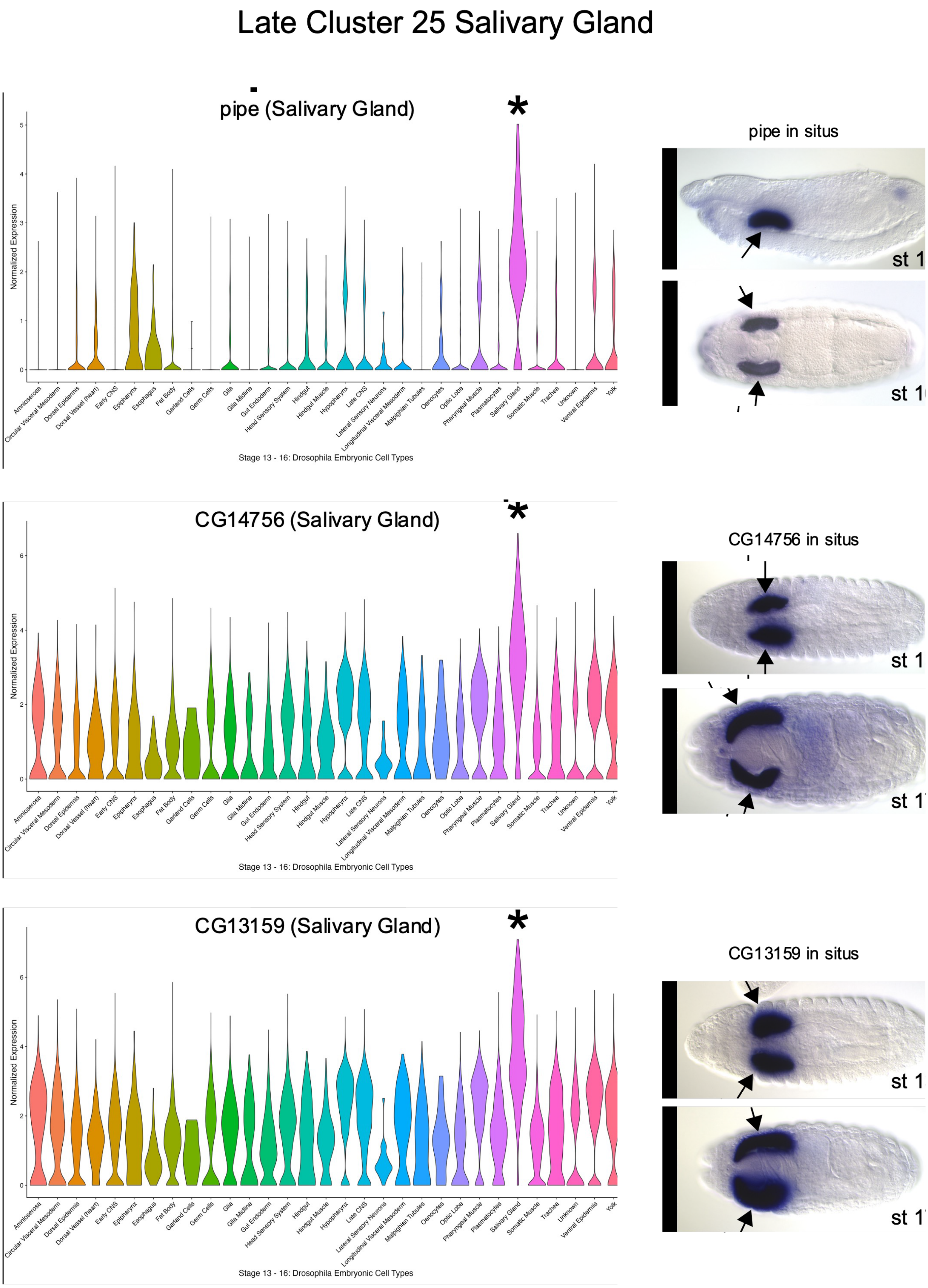

Fig. S21. Violin plots (on the left) and in-situs images from BDGP (on the right) of late SG marker genes: *pipe* (top), *CG14756* (middle) and *CG13159* (bottom).

Development • Supplementary information

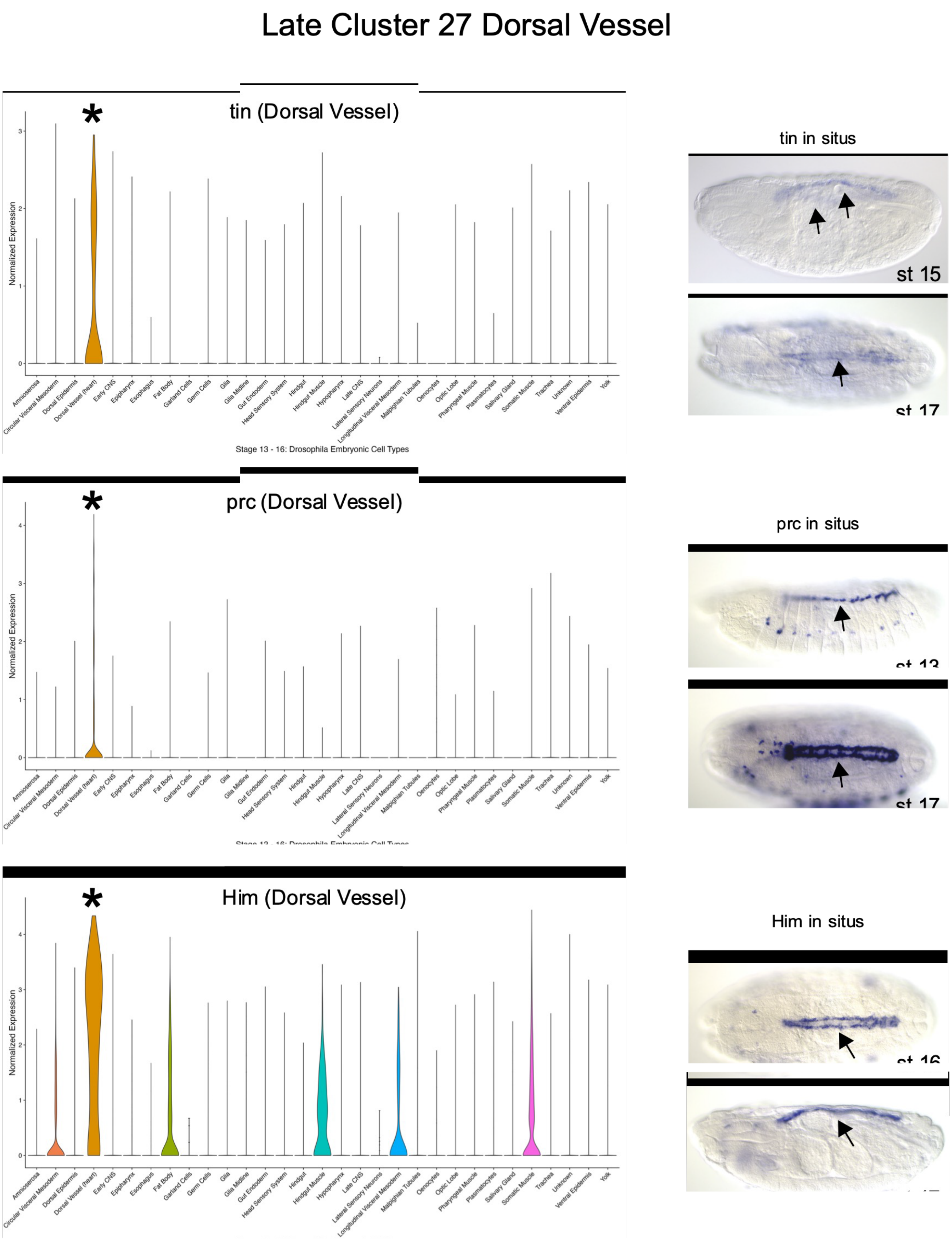

Fig. S22. Violin plots (on the left) and in-situs images from BDGP (on the right) of late dorsal vessel marker genes: *tin* (top), *prc* (middle) and *Him* (bottom).

Development • Supplementary information

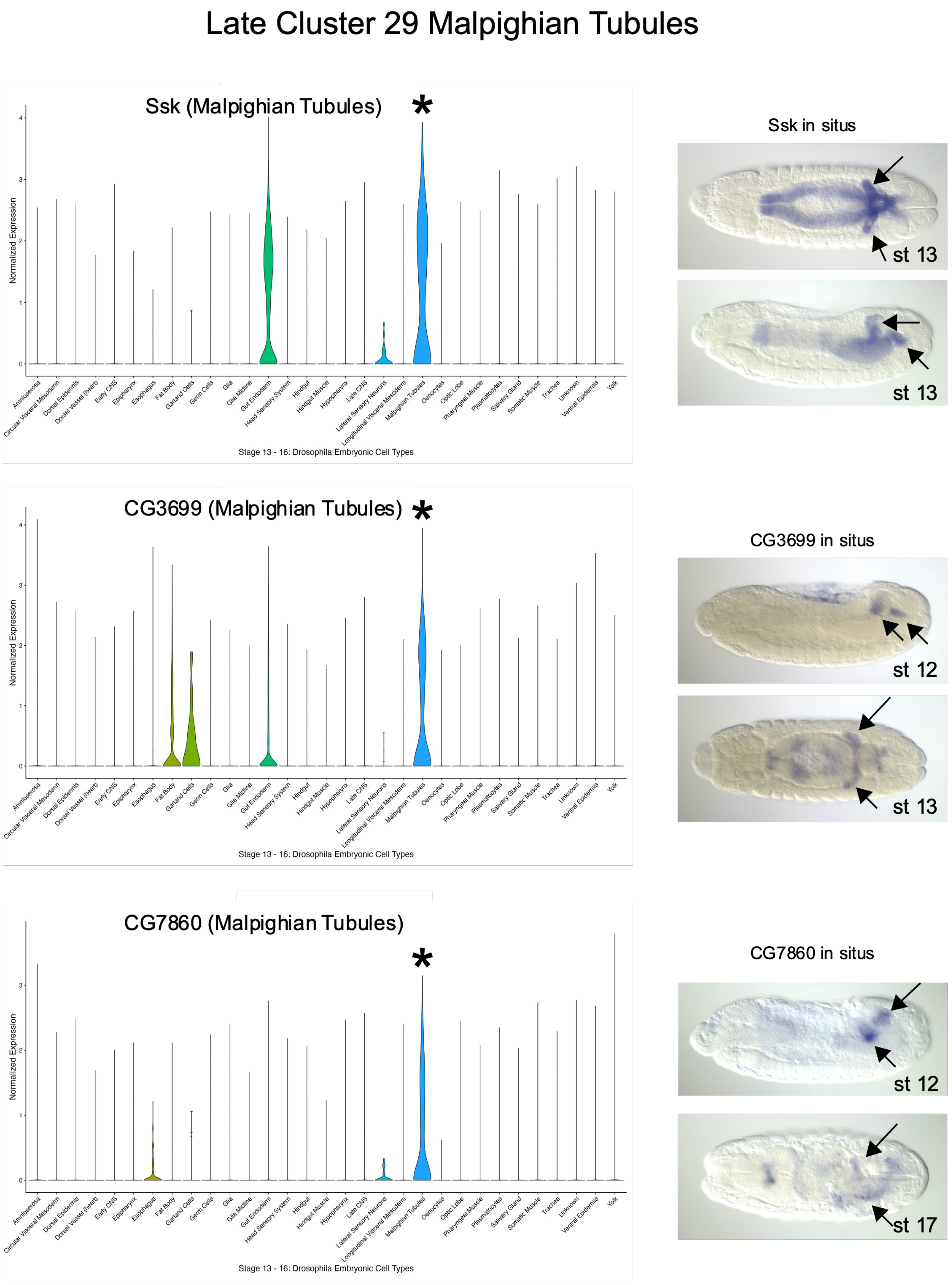

Fig. S23. Violin plots (on the left) and in-situ images from BDGP (on the right) of late Malpighian tubules marker genes: *Ssk* (top), *CG3699* (middle) and *CG7860* (bottom).

Development • Supplementary information

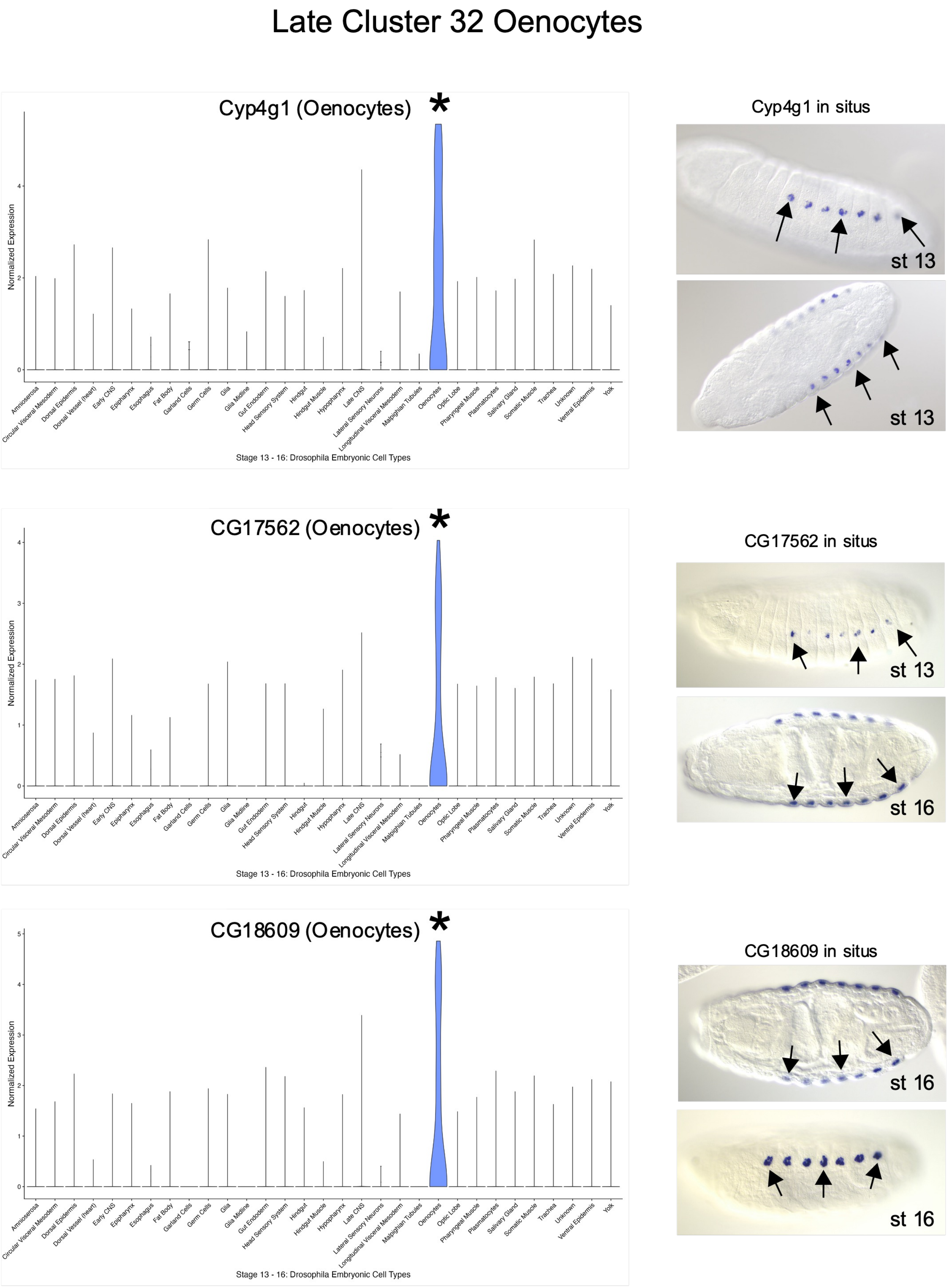

Fig. S24. Violin plots (on the left) and in-situs images from BDGP (on the right) of late oenocytes marker genes: *Cyp4g1* (top), *CG17562* (middle) and *CG18609* (bottom).

Development • Supplementary information

Late Cluster 35 Garland Cells

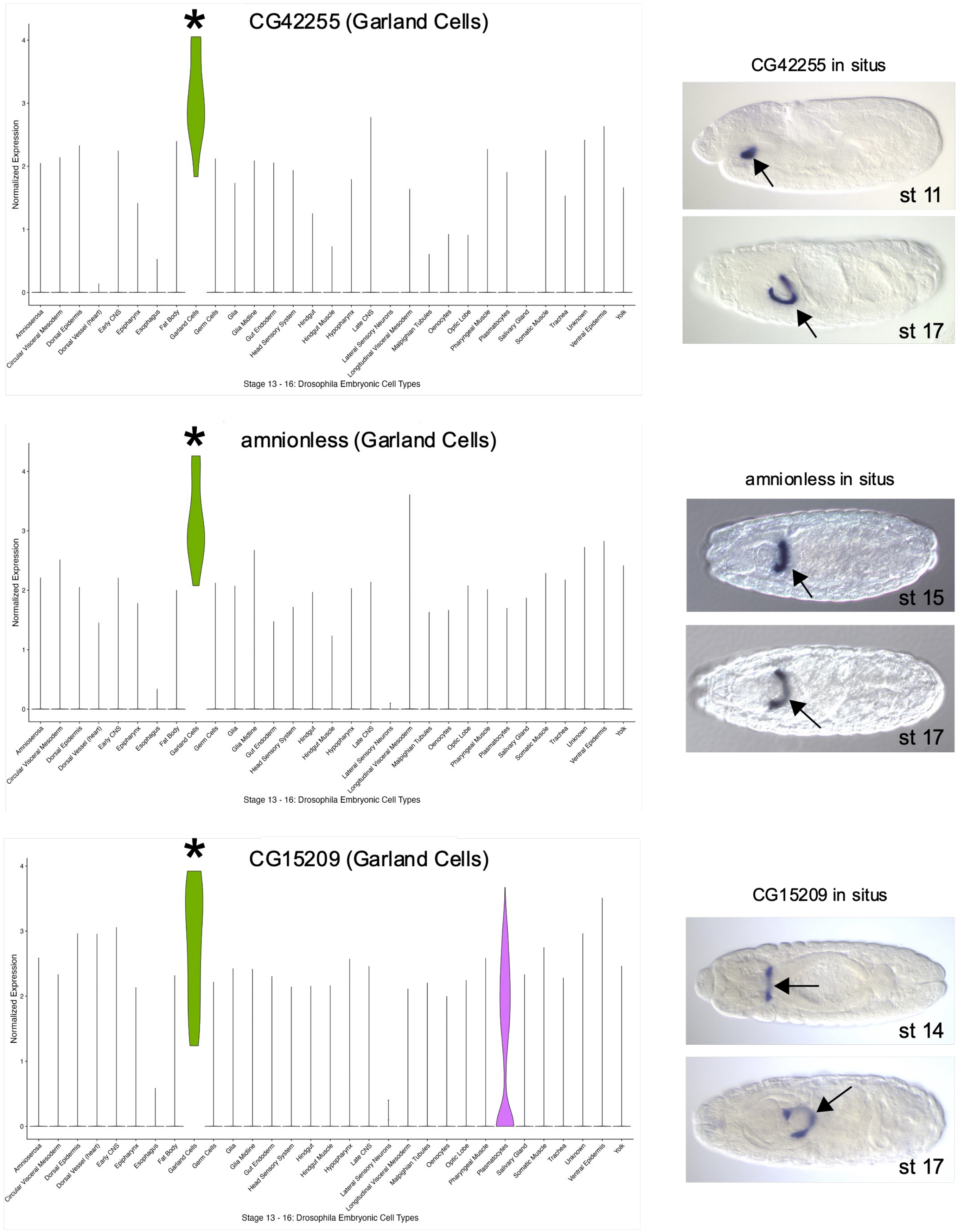

Fig. S25. Violin plots (on the left) and in-situ images from BDGP (on the right) of late garland cells marker genes: *CG42255* (top), *amnionless* (middle) and *CG15209* (bottom).

Development • Supplementary information

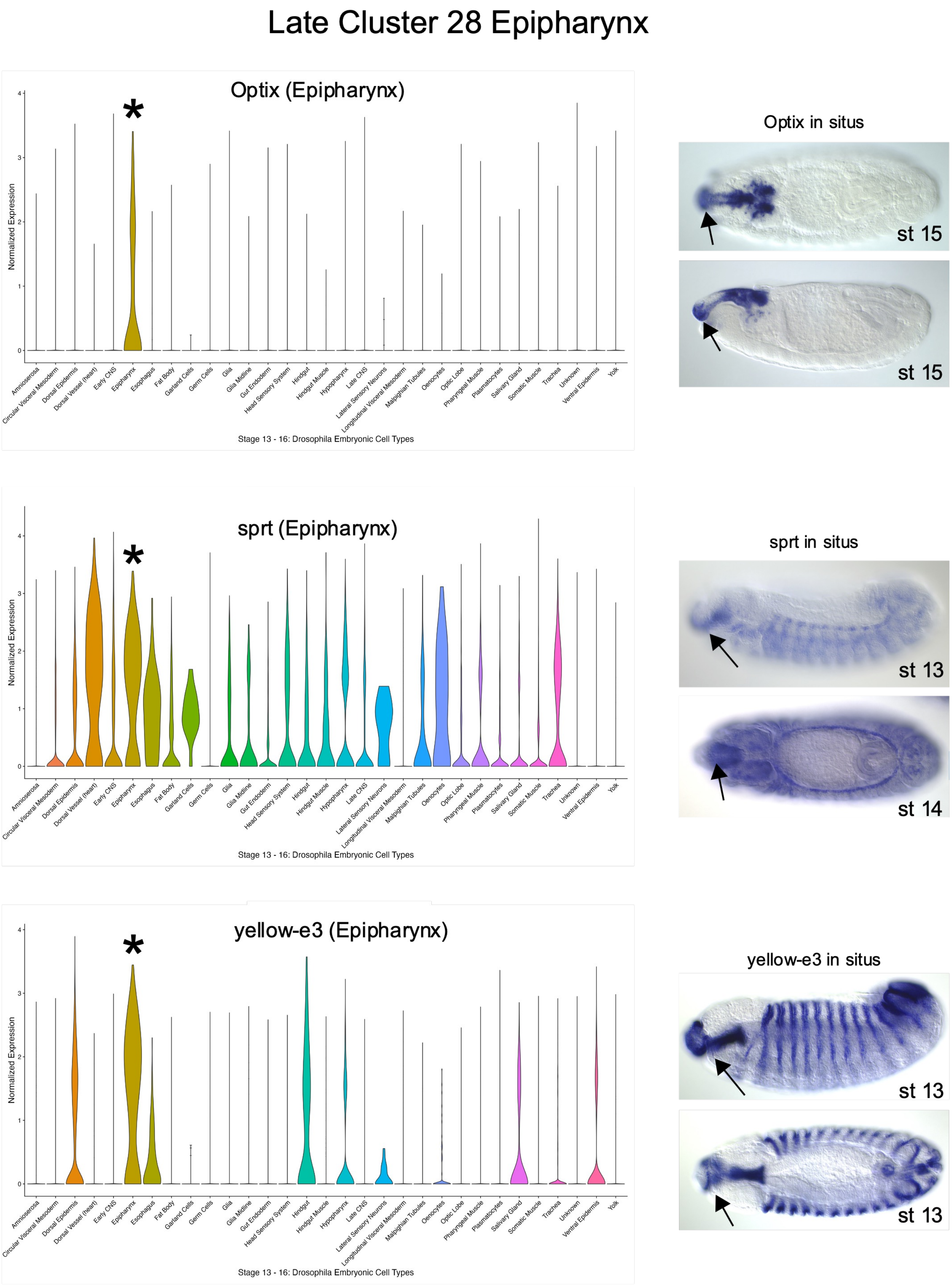

Fig. S26. Violin plots (on the left) and in-situ images from BDGP (on the right) of late epipharynx marker genes: *Optix* (top), *sprt* (middle) and *yellow-e3* (bottom).

Development • Supplementary information

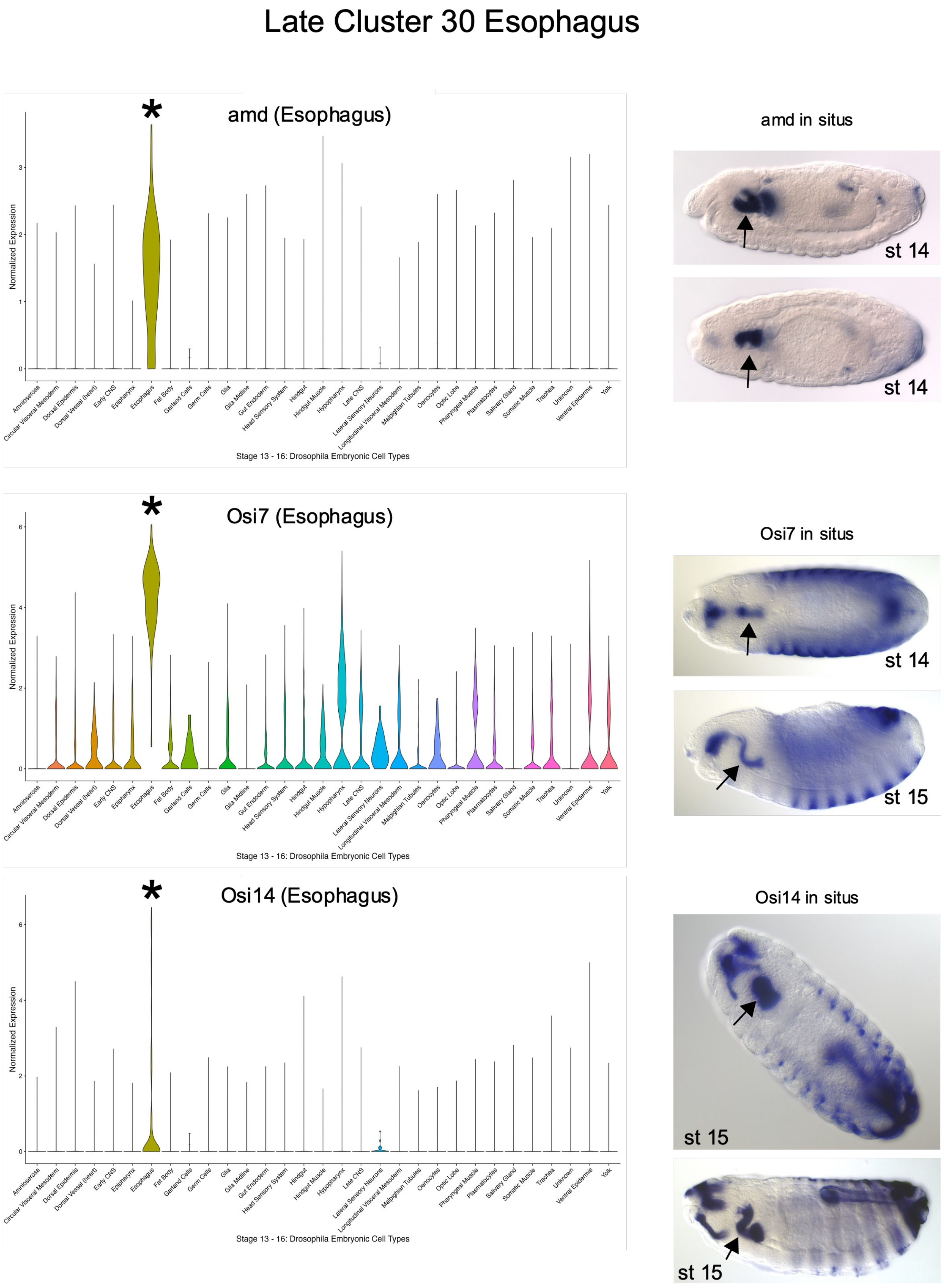

Fig. S27. Violin plots (on the left) and in-situs images from BDGP (on the right) of late esophagus marker genes: *amd* (top), *Osi7* (middle) and *Osi14* (bottom).

Development • Supplementary information

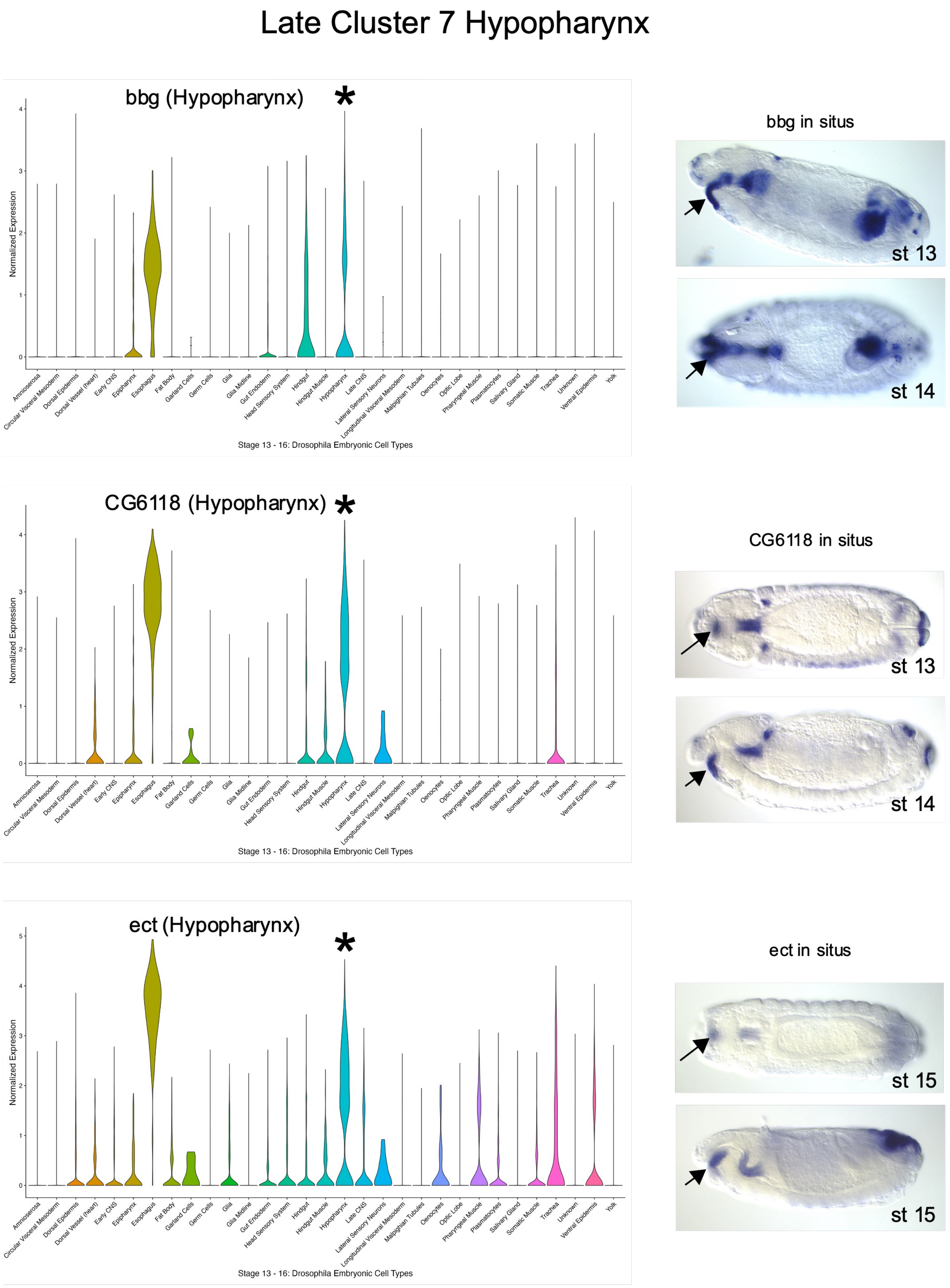

Fig. S28. Violin plots (on the left) and in-situ images from BDGP (on the right) of late hypopharynx marker genes: *bbg* (top), *CG6118* (middle) and *ect* (bottom).

Development • Supplementary information

Late Cluster 21 Hindgut

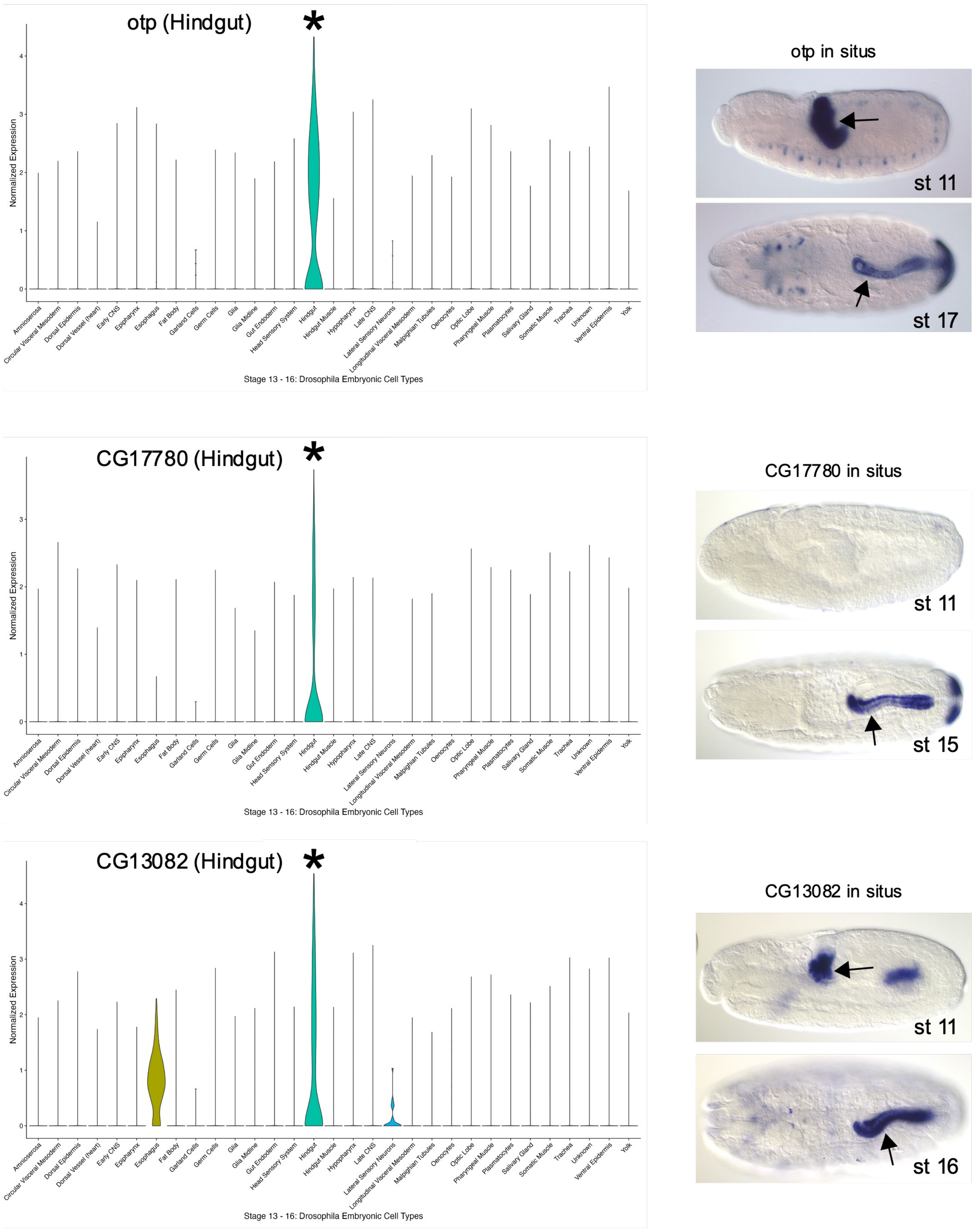

Fig. S29. Violin plots (on the left) and in-situs images from BDGP (on the right) of late hindgut marker genes: *otp* (top), *CG17780* (middle) and *CG13082* (bottom).

Development • Supplementary information

Late Cluster 26 Hindgut Muscle

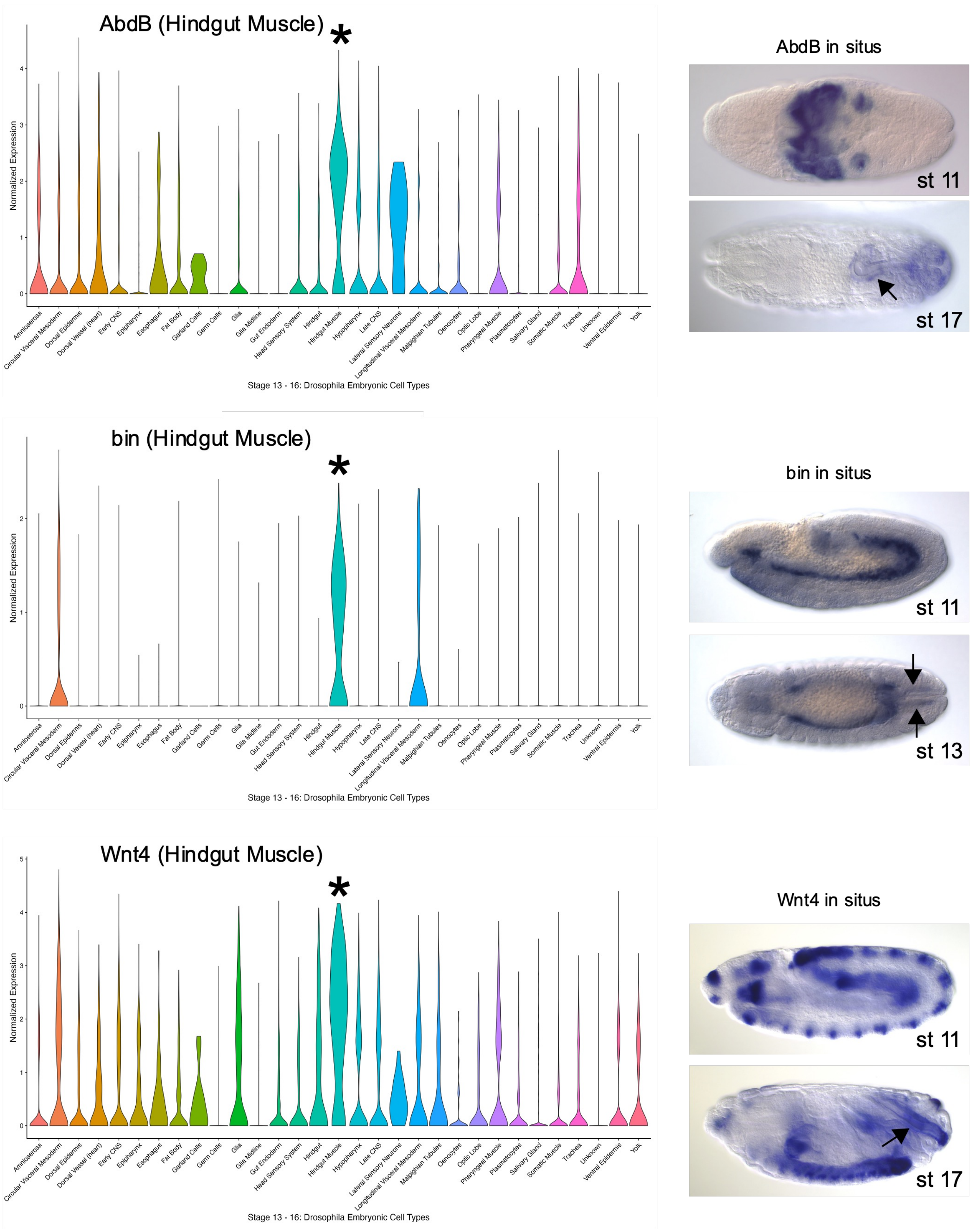

Fig. S30. Violin plots (on the left) and in-situs images from BDGP (on the right) of late hindgut muscle marker genes: *AbdB* (top), *bin* (middle) and *Wnt4* (bottom).

Development • Supplementary information

Development: doi:10.1242/dev.202097: Supplementary information

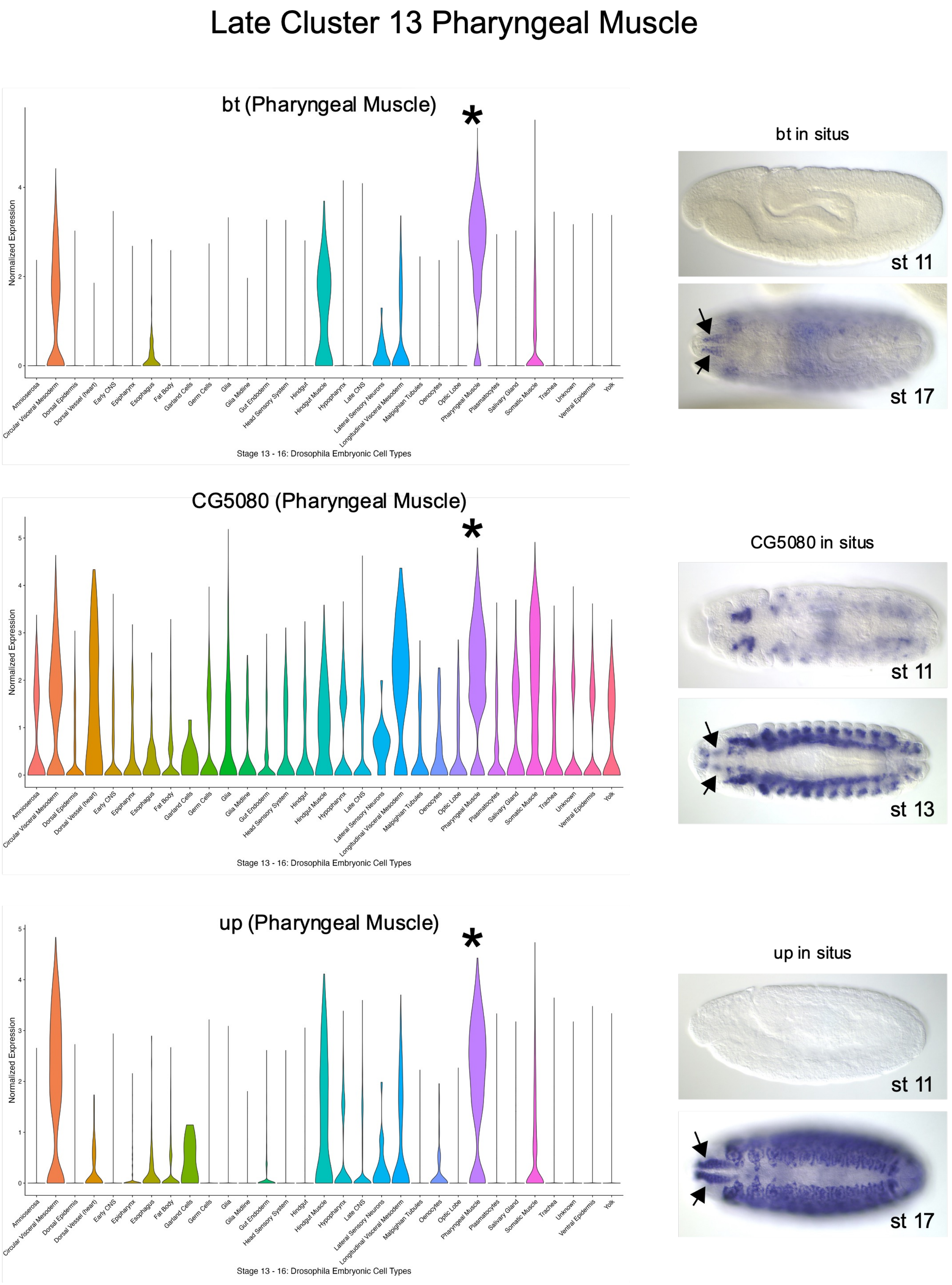

Fig. S31. Violin plots (on the left) and in-situs images from BDGP (on the right) of late pharyngeal muscle marker genes: *bt* (top), *CG5080* (middle) and *up* (bottom).

Development • Supplementary information

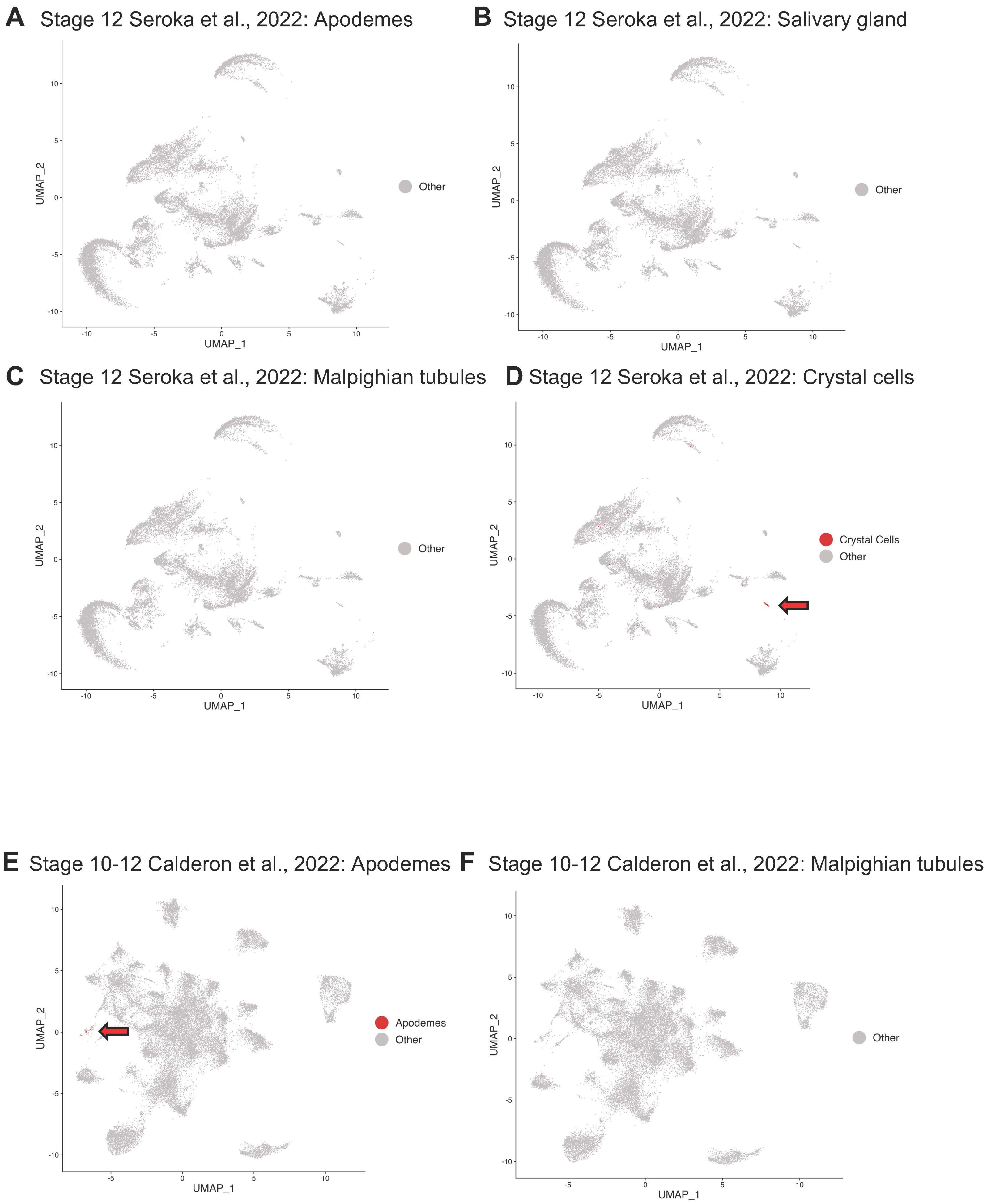

Fig. S32. Classification results of SingleCellNet classifier trained on our data and applied to the data from Seroka et al and Calderon et al. UMAPs showing stage 12 embryonic cells from Seroka et al that are classified as (A) apodemes, (B) SG, (C) Malpighian tubules, and (D) crystal cells. UMAPs showing stage 10-12 embryonic cells from Calderon et al that are classified as (E) apodemes and (F) Malpighian tubules.

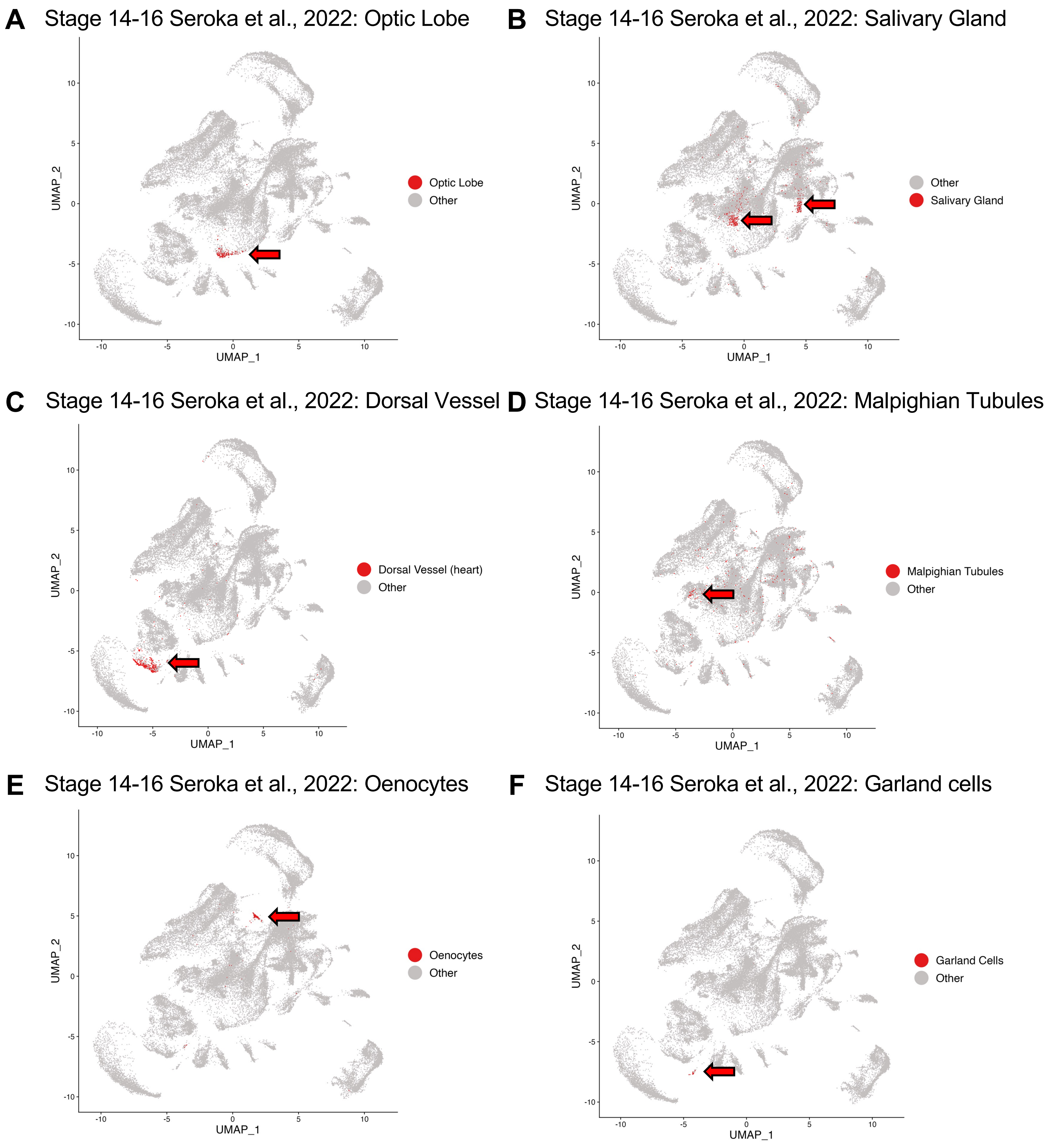

Fig. S33. Classification results of SingleCellNet classifier trained on our data and applied to the data from Seroka et al. UMAPs showing stage 14-16 embryonic cells from Seroka et al that are classified as (A) optic lobe, (B) salivary gland, (C) dorsal vessel, (D) Malpighian tubules, (E) oenocytes and (F) garland cells.

Development • Supplementary information

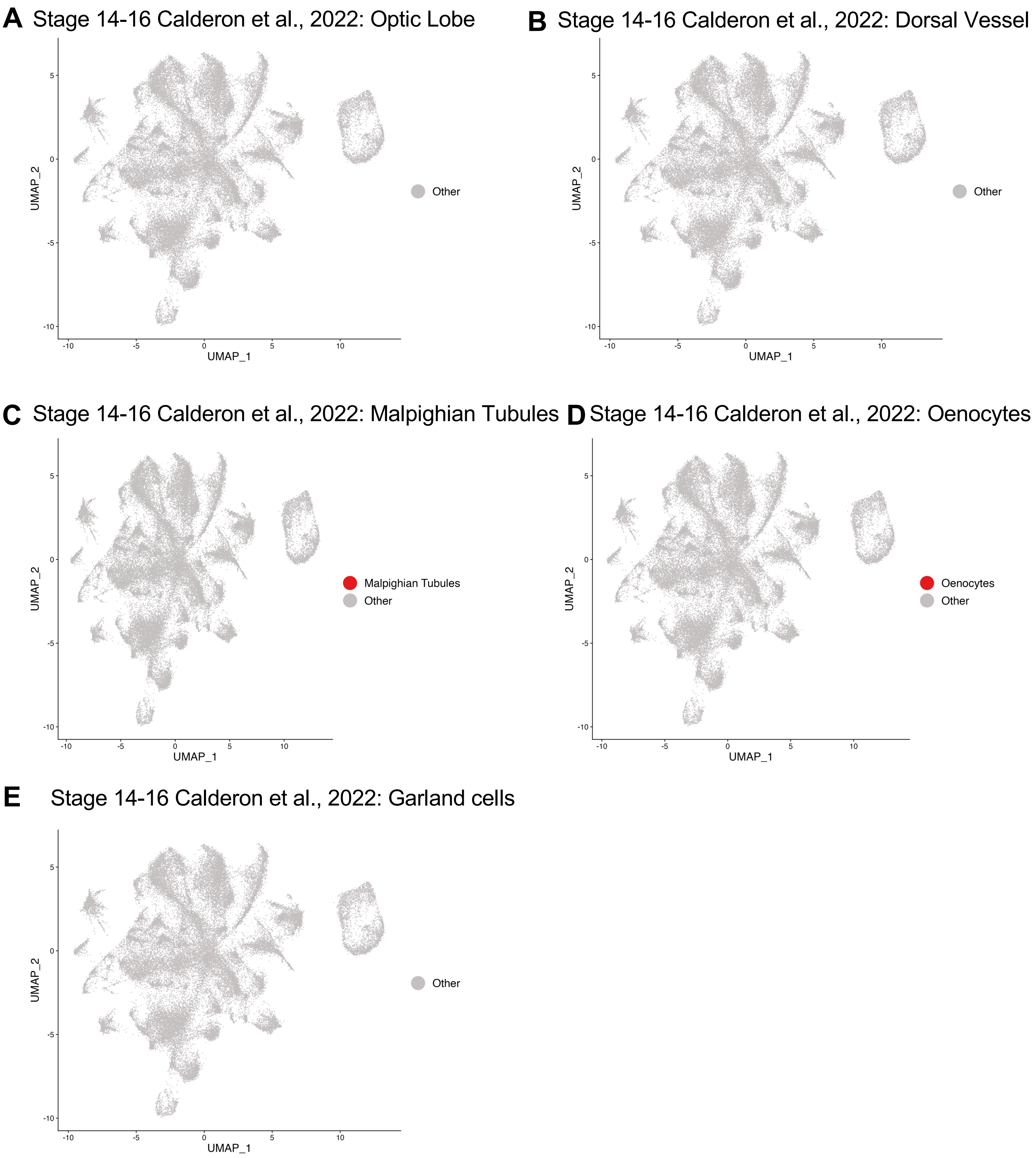

Fig. S34. Classification results of SingleCellNet classifier trained on our data and applied to the data from Calderon et al. UMAPs showing stage 14-16 embryonic cells from Calderon et al that are classified as (A) optic lobe, (B) dorsal vessel, (C) Malpighian tubules, (D) oenocytes and (E) garland cells.

A Average chromosomal expression ratio of Unknown 1 GCs / major trajectory GCs

	3L	3R	2L	2R	4	X
chromosomal expression ratio	0.77	0.75	0.79	0.80	0.81	0.74

B Average chromosomal expression ratio of Unknown 2 GCs / major trajectory GCs

	3L	3R	2L	2R	4	X
chromosomal expression ratio	0.89	0.84	0.86	0.88	1	0.85

C Female marker genes from sexed samples (Li et al, 2021)

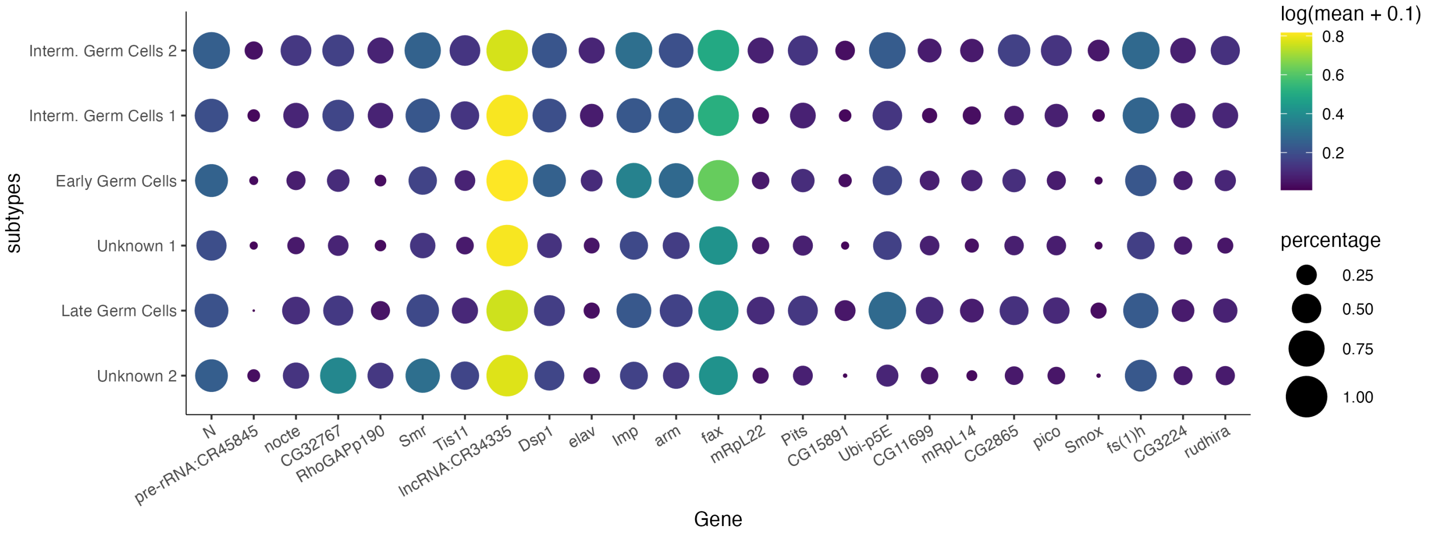

D Male marker genes from sexed samples (Li et al, 2021)

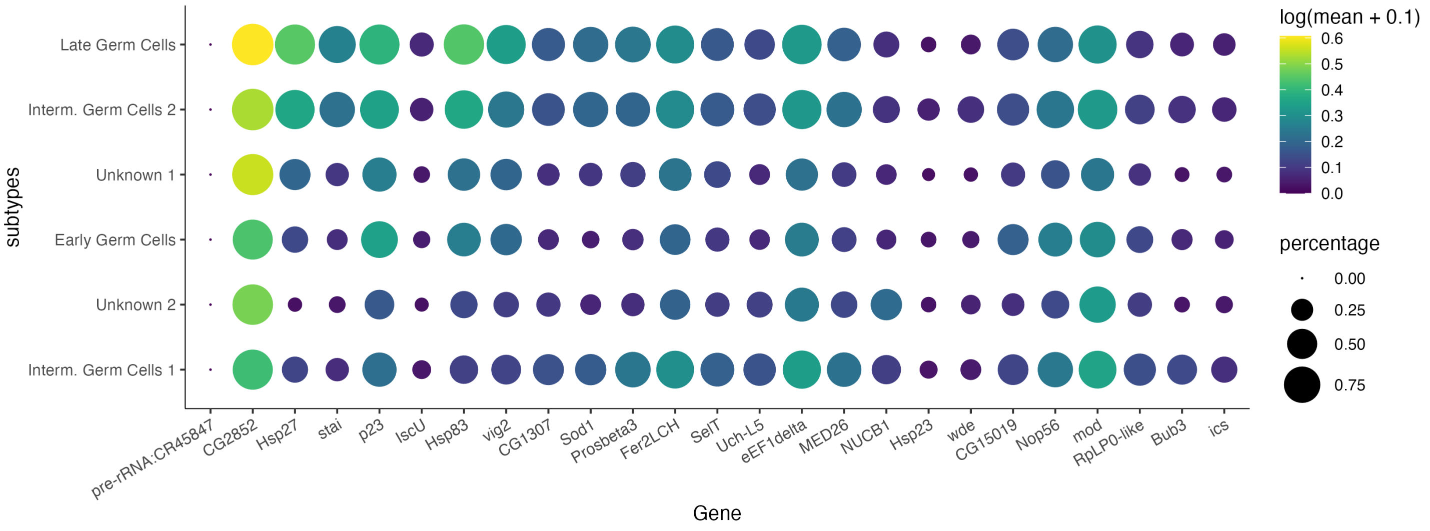

Fig. S35. Ratio of average chromosomal expression of (A) Unknown 1 germ cells to the main trajectory germ cells (Early Germ Cells, Interm. Germ Cells 1, Interm. Germ Cells 2, Late Germ Cells) and (B) Unknown 2 germ cells to the main trajectory germ cells. Dot plots of (C) female marker genes and (D) male marker genes derived from sexed samples from Li et al, 2021. The size of the dot represents the percentage of cells in the sub-population expressing the gene and the color of the dot represents the average expression of the gene in the sub-population.

Development • Supplementary information

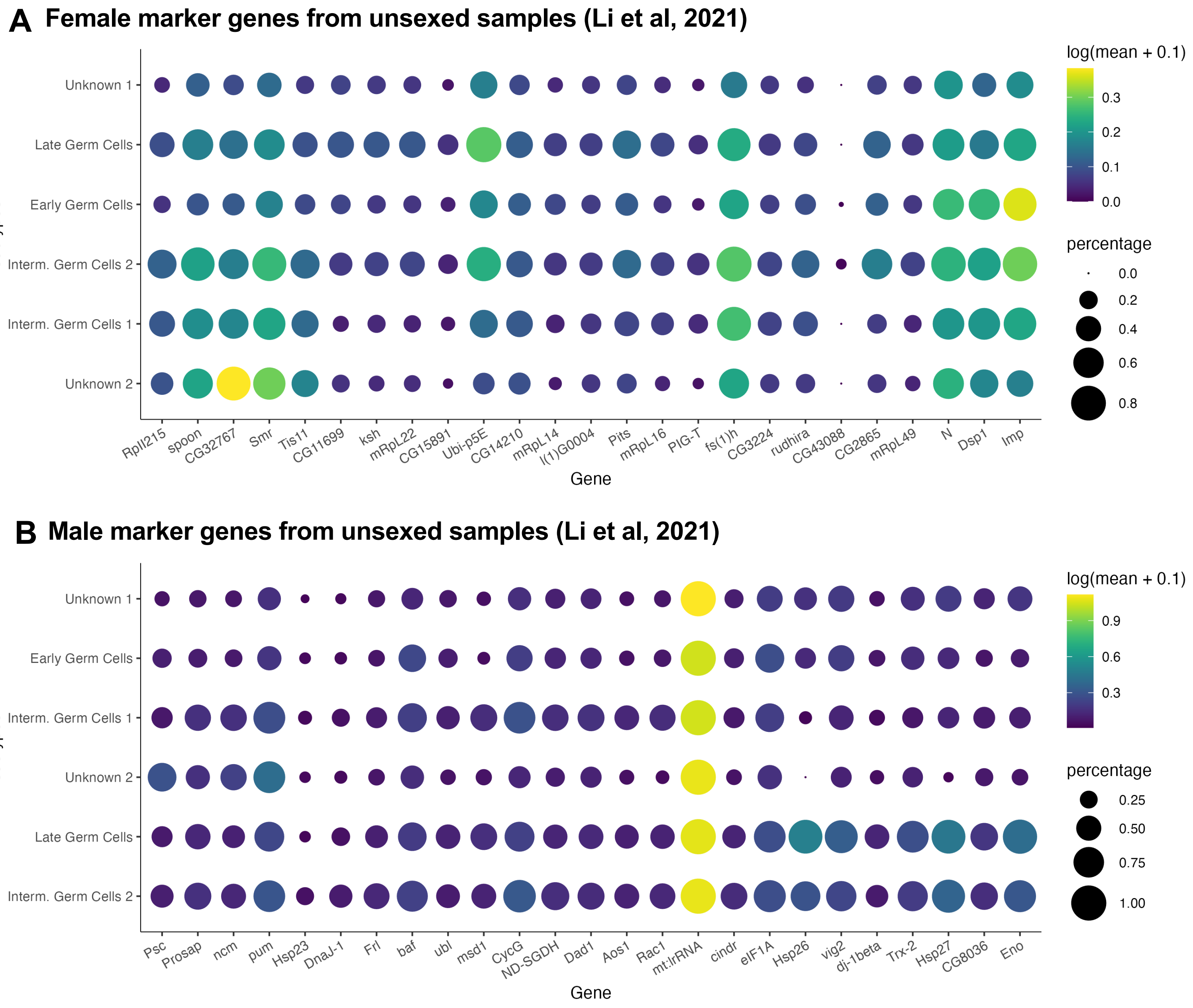

Fig. S36. Dot plots of (A) female marker genes and (B) male marker genes derived from unsexed samples from Li et al, 2021. The size of the dot represents the percentage of cells in the sub-population expressing the gene and the color of the dot represents the average expression of the gene in the sub-population.

Development • Supplementary information

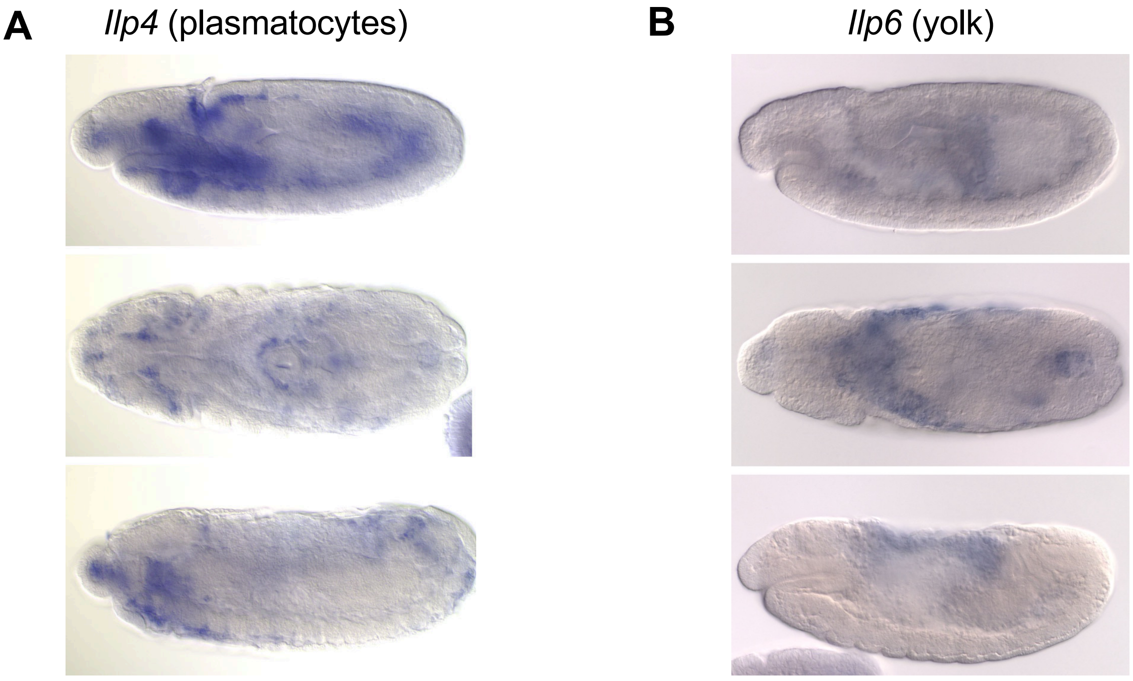

Fig. S37. Newly available in situ images of (A) *Ilp4* and (B) *Ilp6* show expression in plasmatocytes and presumptive yolk, respectively. Note that BDGP calls *Ilp6* expression as amnioserosa, not yolk.

Development • Supplementary information

Table S1. Experimental details of embryo collections.

Available for download at
https://journals.biologists.com/dev/article-lookup/doi/10.1242/dev.202097#supplementary-data

Table S2. Differentially expressed genes for individual clusters relative to rest of the embryonic cells in stage 10-12 and stage 13-16 embryos.

Available for download at
https://journals.biologists.com/dev/article-lookup/doi/10.1242/dev.202097#supplementary-data

Table S3. Selected markers genes for individual clusters in stage 10-12 and stage 13-16 embryos that are cross-referenced with BDGP for cell type identification.

Available for download at
https://journals.biologists.com/dev/article-lookup/doi/10.1242/dev.202097#supplementary-data

Table S4. GSEA results for SG, trachea and germ cells relative to rest of the embryonic cells in stage 10-12 and stage 13-16 embryos.

Available for download at
https://journals.biologists.com/dev/article-lookup/doi/10.1242/dev.202097#supplementary-data

Table S5. Differentially expressed genes for SG sub-populations (early and late SG cells) relative to all other SG cells.

Available for download at
https://journals.biologists.com/dev/article-lookup/doi/10.1242/dev.202097#supplementary-data

Table S6. GSEA results for different SG sub-populations (early and late SG cells) relative to all other SG cells.

Available for download at
https://journals.biologists.com/dev/article-lookup/doi/10.1242/dev.202097#supplementary-data

Table S7. Differentially expressed genes for trachea sub-populations (early tracheal cells, intermediate tracheal cells, late tracheal cells, and tracheal tip cells) relative to all other tracheal cells.

Available for download at
https://journals.biologists.com/dev/article-lookup/doi/10.1242/dev.202097#supplementary-data

Table S8. GSEA results for different trachea sub-populations (early tracheal cells, intermediate tracheal cells, late tracheal cells, and tracheal tip cells) relative to all other tracheal cells.

Available for download at
https://journals.biologists.com/dev/article-lookup/doi/10.1242/dev.202097#supplementary-data

Table S9. Differentially expressed genes for germ cells sub-populations (early germ cells, intermediate germ cells 1, intermediate germ cells 2, late germ cells, unknown 1 and unknown 2) relative to all other germ cells.

Available for download at
https://journals.biologists.com/dev/article-lookup/doi/10.1242/dev.202097#supplementary-data

Table S10. GSEA results for germ cell sub-populations (early germ cells, intermediate germ cells 1, intermediate germ cells 2, late germ cells, unknown 1 and unknown 2) relative to all other germ cells.

Available for download at
https://journals.biologists.com/dev/article-lookup/doi/10.1242/dev.202097#supplementary-data

Table S11. The average expression and cell percent expression of all matrisome genes shown in Figure 6A, S10A, S12 for different cell types in stage 10-12 embryos.

Available for download at
https://journals.biologists.com/dev/article-lookup/doi/10.1242/dev.202097#supplementary-data

Development • Supplementary information

Table S12. The average expression and cell percent expression of all matrisome genes shown in Figure 6B, S10B, S13 for different cell types in stage 13-16 embryos.

Available for download at
https://journals.biologists.com/dev/article-lookup/doi/10.1242/dev.202097#supplementary-data

Table S13. Cell type harmonization for Seroka et al, Calderon et al and this study.

Available for download at
https://journals.biologists.com/dev/article-lookup/doi/10.1242/dev.202097#supplementary-data
