# Supplementary material for: Organogenetic transcriptomes of the Drosophila embryo at single cell resolution
Source: Development. 2024 Jan 19;151(2):dev202097. doi: 10.1242/dev.202097 (PMC10820837; doi:10.1242/dev.202097)
Supplement: Supplementary file 1 [file develop-151-202097_review_history.pdf]

Organogenetic transcriptomes of the Drosophila embryo at single cell resolution

Da Peng, Dorian Jackson, Bianca Palicha, Eric Kernfeld, Nathaniel J Laughner, Ashleigh Shoemaker, Sue Celniker, Rajprasad Loganathan, Patrick Cahan and Deborah Andrew
DOI: 10.1242/dev.202097

Editor: Thomas Lecuit

Review timeline

Original submission:	16 June 2023
Editorial decision:	11 August 2023
First revision received:	10 October 2023
Accepted:	19 December 2023

Original submission

First decision letter

MS ID#: DEVELOP/2023/202097

MS TITLE: Organogenetic transcriptomes of the Drosophila embryo at single cell resolution

AUTHORS: Da Peng, Dorian Jackson, Bianca Palicha, Eric Kernfeld, Nathaniel J Laughner, Ashleigh Shoemaker, Rajprasad Loganathan, Patrick Cahan, and Deborah Andrew

I apologise for the delay in getting back to you. I have now received all the referees' reports on the above manuscript, and have reached a decision. The referees' comments are appended below, or you can access them online: please go to BenchPress and click on the 'Manuscripts with Decisions' queue in the Author Area.

As you will see, the referees express interest in your work, but two of them in particular have some important criticisms and recommend a substantial revision of your manuscript before we can consider publication. If you are able to revise the manuscript along the lines suggested, which may involve further experiments, I will be happy receive a revised version of the manuscript. Your revised paper will be re-reviewed by one or more of the original referees, and acceptance of your manuscript will depend on your addressing satisfactorily the reviewers' major concerns. Please also note that Development will normally permit only one round of major revision. If it would be helpful, you are welcome to contact us to discuss your revision in greater detail. Please send us a point-by-point response indicating your plans for addressing the referees' comments, and we will look over this and provide further guidance.

Please attend to all of the reviewers' comments and ensure that you clearly highlight all changes made in the revised manuscript. Please avoid using 'Tracked changes' in Word files as these are lost in PDF conversion. I should be grateful if you would also provide a point-by-point response detailing how you have dealt with the points raised by the reviewers in the 'Response to Reviewers' box. If you do not agree with any of their criticisms or suggestions please explain clearly why this is so.

Reviewer 1

Advance summary and potential significance to field

© 2023. Published by The Company of Biologists under the terms of the Creative Commons Attribution License (https://creativecommons.org/licenses/by/4.0/).

In this manuscript the authors present a single cell RNA-sequencing study covering broadly the time of organogenesis in Drosophila melanogaster. They sample two timepoint, stages 10-12 when organ primordia on average are specified, and stage 13-16, when morphogenesis of many tissues has neared its end and functioning begins.

Following on from scRNAseq the authors now present an in depth analysis of the data, focussing particularly on a subset of tissues, salivary glands, tracheae and germ cells, as well as the matrisome, the collective group of everything secreted in connection with extracellular matrix, and they suggest the origin of several commonly used tissue Drosophila culture cell lines.

The sequencing analysis is based on large numbers of cells and good read depth and the analysis of the data is mostly very thorough. Apart from stainings (in situ and protein) to illustrate the development of salivary glands, tracheal system and germline all analysis and inference is based on the sequencing data and publicly available in situ analyses (at BDGP).

The publicly available sequencing data provided here, together with this initial analysis will provide a great resource to the Drosophila community. As the authors discuss, there are thus far only few scRNAseq studies of Drosophila embryogenesis, and apart from one they tend to focus on specific stages or tissues, hence this global analysis is an important addition.

Comments for the author

In this manuscript the authors present a single cell RNA-sequencing study covering broadly the time of organogenesis in Drosophila melanogaster. They sample two timepoint, stages 10-12 when organ primordia on average are specified, and stage 13-16, when morphogenesis of many tissues has neared its end and functioning begins.

Following on from scRNAseq the authors now present an in depth analysis of the data, focussing particularly on a subset of tissues, salivary glands, tracheae and germ cells, as well as the matrisome, the collective group of everything secreted in connection with extracellular matrix, and they suggest the origin of several commonly used tissue Drosophila culture cell lines.

The sequencing analysis is based on large numbers of cells and good read depth and the analysis of the data is mostly very thorough. Apart from stainings (in situ and protein) to illustrate the development of salivary glands, tracheal system and germline all analysis and inference is based on the sequencing data and publicly available in situ analyses (at BDGP).

The publicly available sequencing data provided here, together with this initial analysis will provide a great resource to the Drosophila community. As the authors discuss, there are thus far only few scRNAseq studies of Drosophila embryogenesis, and apart from one they tend to focus on specific stages or tissues, hence this global analysis is an important addition. But it is really a resource, and so should be published as such, und er the ‘Techniques and Resources’ section rather than as a research article as it currently seems to be. For a research article, more follow up analyses with further experiments, rather than comparison to and analysis of published data, should have been provided.

Below I will discuss some general comments about this study, as well as detailed comments to individual parts of the analysis and presentation.

*) At times, sections of this manuscript read rather like a large review on the different tissues and processes analysed, this is true for the introduction (sections on page 4 and 5) as well as the discussion (page 24/25:
these pages have a very long discussion of GC knowledge and studies that bears little relation to the manuscript here and would be better in a GC review, this should be condensed).

*) Generally, some conclusions appear overstated, given that all is based on scRNAseq and published in situ images. At times, as pointed out in the detailed comments, conclusion should be phrased more cautiously especially where other studies and data are criticized and stated to be incorrect, but without the authors here actually providing the evidence.

© 2023. Published by The Company of Biologists under the terms of the Creative Commons Attribution License (https://creativecommons.org/licenses/by/4.0/).

*) A general comments about the staging of batches and their relative position along developmental time as well as pseudotime analysis:

Looking at the batches overlaid onto the UMAP of the salivary gland cells as shown in Suppl. Fig.2A and the pseudotime plot in S2B, both panels clearly indicate that the batches mostly split into two groups of either early or late cells, with a small amount of overlap provided by batch 2 of the stage 13-16 embryos. If looked at in a third dimension, is the stage 13-16/rep2 separate from the two early batches? Panel B would suggest possibly yes. I am unsure of for the analysis of rather few cells here, the pseudotime adds any value beyond the positioning along developmental time that the natural separation of the batches already provides.

This seems evident in panels S2C and that basically only show a split along batches and thus what is expressed in early cells and what in late cells. Everything seems to nearly come on at the same time. It might make more sense to focus a pseudotime, if at all here, on either early or late batches individually, but that again might not be possible due to the sparsity of cells obtained for the salivary glands.

Following from this, I wonder whether the authors visually screened the embryos submitted for sequencing before dissociation of have matching IF images? It seems surprising that two batches that were meant to be identical (especially looking at Suppl. Table 1), i.e. the two late batches, were so different in their actual developmental staging.

Detailed comments on introduction, results and discussion:

1) results page 7: In the first paragraph here the number of cell type clusters identified is listed for the two different time points sampled.

The deduction of cell clusters and identities should be better explained and shown. Table 2 is just a very long list, it might be useful to split it into the different tissues for easier accessibility, especially if this is a resource, and also to have the column labels fixed floating at the top rather than part of the table that disappears when you scroll.

For each of the subsequent more detailed sequencing data analysis and conclusions drawn from the data for the salivary glands, tracheae, germline, it would be good to state what the most important, highest differentially expressed markers were that identified these tissues or rather their precursors especially at the early stages. The precursor populations are likely already to some extent non-homogeneous based on the vast literature that is already out there for these tissues and their development. Is for instance Scr or fkh expressed in all cells of the (early) salivary gland cluster defined, is trh or FGFR in all the cells analysed as the tracheal cluster, is vasa etc. in all the ones analysed as germ cells etc? The author do not show a single overlay onto the UMAP of expression of some of their top marker genes for any cluster generated for early and late timepoints. This would be very useful to provide, so readers can judge how specific expression of these marker genes is, and it would also be a good part of a resource. The paper already has a vast number of supplemental figures, so adding these can't hurt.

2) Salivary glands analysis, page 8:

The authors draw a lot of conclusions from the analysis of rather a very small number of cells here. How sure are they that representative cells for possibly different regions of the tissue precursor or forming tissue were captured here? 114 and 271 cells respectively appears small.

3) Middle of page 9: the authors state that the downregulation of several salivary transcription factors is a new revelation of their findings, but of the ones they list and show with BDGP in situs, many were published to behave like this previously, including by the authors themselves:

hkb was published before, (Myat et al., two papers in 2000), as was eyegone (Jones and Beckendorf, 1998), equally Habermann et al 2003 show that trh is only in duct late, and Scr is beautifully shown in Henderson and Andrew, 2000.

© 2023. Published by The Company of Biologists under the terms of the Creative Commons Attribution License (https://creativecommons.org/licenses/by/4.0/).

This statement should be adjusted.

The authors also then come back to this in the discussion (page 21), where they discuss how expression differences early versus late might be regulated. I would question what if this difference in mRNA expression is important for function of these transcription factors? From the authors' own publications, it looks as if at the protein level, at least Fkh and CrebA remain strongly present throughout. Could you comment on this?

4) Page 11, tracheal analysis, cross correlation analysis:

This cross correlation analysis is not well explained here, in the figure legend or the methods (where it is only said that this and this tool is used). I find it impossible to tell if this analysis, based on pseudotime (being is a fairly subjective way of analysis), with production of fairly similar albeit not identical curves reflecting expression of many different genes in each functional group, can really tell you anything about temporal staggering and roles of different functional gene sets.

Please explain more here, so the reader can judge how meaningful this analysis is.

Following on from this, the concluding statement says:

'Altogether, our analysis of the tracheal organogenetic transcriptome reveals the subtle dynamics that underlie the regulatory signature of growth…'.

This is an overstatement and should be phrased more cautiously. Without further follow-on experimental analysis, this is based on pseudotime and cross correlation and in my view can therefore only suggest such dynamics rather than 'reveal' it.

5) Page 12, GC analysis: as with the two previous tissues, it would be worth stating here what the top genes were that the identification of the GCs was based on in the initial analysis.

6) Page 16, conclusion:

What about matrisome expression and ECM secretion by other cells than the plasmatocytes you mostly focus on? The data in Figure 7 clearly show expression also in other tissues/cells, as is also clear from the literature so this should at least be mentioned.

7) Discussion page 22 bottom:

The authors conclude from their expression analysis and published data that expression of e.g. laminin in plasmatocytes must be carefully spatially coordinated with for instance trachea. My understanding is that for many tissues in the embryo, ECM deposition is controlled by expression of integrins that then capture the hemocyte secreted ECM, thus regulation of integrin expression in the tracheal cell might be all that is required rather than complex spatial coordination?

8) Discussion, page 223 bottom:

The authors compare their inference from GC expression data to published studies and disagree with those studies' conclusions.

If the authors want to contrast their findings to a previously published study, shouldn't their data be shown rather than' data not shown'? Also, what are the technical differences between the two analyses? Number of cells sequenced? Read depth? It would be important to comment on this.

Also, first sentence on page 24 'Finding multiple distinct GC populations in our data…' This should be phrased more cautiously, i.e. 'finding multiple suspected or predicted distinct GC populations...' until there is further corroborating data such as in situs or IF of some of the differentially expressed genes actually labeling physically distinct cell populations.

9) Discussion, page 26, just above concluding remarks:

© 2023. Published by The Company of Biologists under the terms of the Creative Commons Attribution License (https://creativecommons.org/licenses/by/4.0/).

The authors disagree with the conclusions of another study, suggesting a Gal4 driver expressed in more cells than assumed led to aberrant conclusions.

Again, this should be phrased more cautiously or suggestive if the authors do not actually show any analysis of srp-Gal4 expression in apodemes but only speculate that this is the underlying cause of the discrepancy!

Detailed comments on methods:

10) Page 29, the authors state ‘ The cluster marker genes were matched with a table of marker genes of various cell types curated from the BDGP database’’ :

Where can this table of marker genes be found and how was it curated? The citation is ‘Table S2’, but this is where the results are shown, not this comparative table.

Detailed comments on Figures and Figure Legends:

11) As a general comment, these figure legends are rather sparse, more information on plots, statistics, what individual panels show etc etc should be provided!

12) Figure 3E and all related dot plots:

This plot and all subsequent related ones need more explanation, citing what the expression and percentage (percentage of cells in cluster expressing gene?) refer to and therefore imply.

13) Figure 3A: In this as in all similar figures/panels (apart form in Figure 1 in the total UMAPs for earlier and later stages) the small coloured dots explaining identity of clusters are too small to be able to identify the colour without having to zoom in, especially in figures like the GC analysis, where there are many clusters and the colours are not that distinct (was a colour-blind friendly colour code taken into account?), can these please be increased in size?

Detailed comments on Figures:

14) Figure 2D and Figure 3E: how do these GO term analyses compare to each other for the salivary glands?
Looking at the scale in both sections, ‘Golgi vesicle transport’ at stage 10-12 in Fig. 2D has a change of ~5 whereas the same GO term in Fig. 3E only has a value of ~1.7, why would that be?

And for stage 13-16 embryos salivary gland, cytoplasmic translation in 2D has a value of ~5 whereas 3E has a value of nearly 30 for the same GO term. Why is that?

15) Dot plots in 3E, 5D and similar:

I find these plots very unintuitive, what about showing the expression of e.g. the four genes in 5D overlaid on the UMAP shown in panel 5A? Or at least show both?

For large scale analyses such as the matrisome these plots make more sense, but for smaller comparison UMAP expression maps are much clearer.

16) Supplementary Figure 3:

Back to general comments, the colour code for the clusters in A is near impossible to match to the plot as the explanatory dots are too small and the colours too similar.

The cluster in the center representing tip cells when looking at the batch plot in B seems to be composed of cells from both the stage 10-12/rep1 and the stage13-16/rep2 cluster. Is there a time progression within this cluster? The two source seem to very clearly split into two, so what is the significance of this?

© 2023. Published by The Company of Biologists under the terms of the Creative Commons Attribution License (https://creativecommons.org/licenses/by/4.0/).

Panel C: This analysis very clearly shows that the repeats of the later batches are very different in timing, so the pseuotime plots presented in Suppl Fig 4 really just seem to represent that. Only the chitin synthesis show any real progression.

17) Supplementary Figure 5:

I am confused by the pseudotime plot in C: why are the cells ordered like this here, with stage 13-16/rep1 appearing to span later developmental time than rep2, when all the previous analyses of salivary gland and tracheae suggested that stage 13-16/rep 2 covers earlier cells than stage 13-16 /rep1? How can this be?
Doesn't the UMAP in B rather suggest that there is a split in the late (green) cluster into different cells?

18) Supplementary Figure 6:

Panel D: the statistical analysis symbols are really hard to see on the background used, please change this.

19) Supplementary Figure 8:

What does the first cluster labelled as ‘artefact’ represent ??

Panel B: the colour scale used for the expression overlay on the UMAP is not ideal. The purple representing no expression is the most obvious colour, with the pale green and even the yellow indicating expression very hard to see, especially where it intersects with purple cells. Non-expressing cells should ideally be a pale colour with expression marked by increasing intensity of a bright colour.

19) Supplementary Figure 12:

Panel B, right hand side: why in this plot does the salivary gland from Calderon highlight all cell types in the stage 13-16 samples, very different to the stage 10-12 sample above?

20) Supplementary Figure 29 and similar:

In these plots, I cannot see what is meant to be shown in A-C, is anything highlighted here? And in panel D, E , where the legend shows two colours for different clusters/cell types it is impossible to distinguish these two colours, please explain, annotate better and use a different colour scheme.

Fig. S30/31 have the same colour scheme problem, he colour that is supposed to highlight expression needs to be clearly different and brighter or darker than the background colour to make this visible.

Little bits:

1) Page 4 bottom , introduction: ‘…the discoveries […] accelerated the process of discovering key players.’
This sentence does not make much sense to me, what do the authors mean?

2) Page 10, end of first paragraph: ‘contiguous’ (i.e. adjacent) makes no sense here, you must mean continuous.

11) methods page30 top, pseudotime analysis:

Various typos or grammatical errors:

‘… was used perform…’, ‘pseodtime’

© 2023. Published by The Company of Biologists under the terms of the Creative Commons Attribution License (https://creativecommons.org/licenses/by/4.0/).

Reviewer 2

Advance summary and potential significance to field

Overall a great paper producing a very useful dataset of embryo scRNAseq, followed by an excellent demonstration of how this can be combined with BDGP in situs for the analysis of organogenesis. The research performed and its presentation are all at the top standard. I am confident it will be of interest to the readers of Development.

Comments for the author

Minor revisions that must be done.
1. Data availability; although putting the raw data in GEO is useful, the authors should also upload it to scRNA expression atlas (https://www.ebi.ac.uk/gxa/sc/home), for several reasons. It will permit researchers with less bioinformatic experience the tools to explore the data. By this database analysing the raw data using the same pipeline, it will also aid comparison with the other published data sets. It will also gain a dataset report in FlyBase via this route.
2. It would be a great help for future curation of this data if the cell types were annotated with the cell type terms from the FlyBase anatomy ontology; assignment of the correct terms could be provided in Supplementary Table 7 3. p7

Table S2, the table needs more explanation of the data in each of the columns. It wasnt clear how the list of genes in this table was produced (top 20-50 differentially expressed genes is vague, and the description in the methods is not that much clearer). This is probably the most generally useful data in this paper, in addition to the raw data, but its not very clear what is being shown. Full names of the genes would also be a helpful addition.
4. p7
" were used to assign 25 distinct cell types (Fig. 1B) from 35 early single-cell clusters (Fig. S1B) and 31 distinct cell types (Fig. 1D) from the 36 late single-cell clusters (Fig. S1D)."
I was not able to relate the 25 to 35 or 31 to 36 from these figures. Perhaps a table is needed? or connect them in one supplementary figure?
5. p7 the statement “the proximity of different clusters in the UMAPs [...] reflect the germ layer of origin, e.g., all mesodermal derivatives [...] are clustered closely”. That may be true but it is dubious whether this has any significance. It is debatable whether proximity of clusters in UMAP embeddings reflect some biological facts about the clusters or are merely consequences of distortions caused by the embedding algorithm (see a recent preprint from Chari & Pachter for intensive discussion on that issue: http://biorxiv.org/lookup/doi/10.1101/2021.08.25.457696).
6. The fact that muscles undergo fusion during these stages of development needs a mention. For example, does that result in a reduced fraction muscle 'cells' relative to using single nuclear RNA sequencing?
7. p13 the last sentence reference to supplementary figure S6C: in figure S6 it is labelled D and there is no C.
8. p15 Section on Basement membrane.
There are only 4 major components of the basement membrane, yet only two are analysed in this section. Add the other: two perlecan/trol and Nidogen
9. It would also be very useful to compare the basement membrane associated laminin alpha chain LanA to the non-basement membrane one wb.
10. I find this statement is going a bit on the wrong track "Given that the primary source of Laminins are the plasmatocytes..." I agree its plasmatocytes produce the highest level, but as clearly indicated in the figure, most cells express laminins, in contrast to other basement membrane components, so its not so clear that the spatial coordination discussed is required.
11. Supplementary figures S2C, S2D and S4, add a label directly in the figure to indicate that the comparison is across the inferred pseudo time (e.g. with a label on the X axis, accompanied by an arrow), instead of just mentioning in the legend.
12. Add the data shown in Figure 6 and supplementary Figures, 7, 9 and 10 as supplementary tsv files, so readers can explore the data themselves, or replot the data.

Suggestions for additional minor revisions.

© 2023. Published by The Company of Biologists under the terms of the Creative Commons Attribution License (https://creativecommons.org/licenses/by/4.0/).

13. I didnt find the second paragraph of the introduction particularly helpful in framing the value of the research presented in this paper; I think it could be removed without detriment. While it is helpful to introduce the BDGP in situs; the third paragraph could also be shortened.
14. Some readers might find it suprising that the tracheal tip cells are similar to axons; so this may merit some further discussion.

Reviewer 3

Advance summary and potential significance to field

In this manuscript, Peng et al. perform single cell RNA-sequencing in Drosophila embryos during two periods of embryogenesis. The single cell RNA-sequencing data is of high quality and will be of interest as a resource to the greater Drosophila community. The authors highlight the sequencing data for three separate cell populations, the salivary glands, the trachea, and the germline. Using GO-term analysis, the authors find that both the salivary glands and the trachea are enriched for GO-terms that are consistent with their function.
They further validate their sequencing data by cross-referencing the BDGP in situ hybridization database to confirm expression of TFs identified in their scRNA-seq data. The authors subcluster the putative germ cell cluster and identify six subclusters to which they assign potential functions. Finally, the authors compare the transcriptomes of five embryonic cell lines with their scRNA-seq data and determine that four out of five of the lines are most closely related to plasmatocytes. While this manuscript represents a quality scRNA-seq data set, the findings presented here do not significantly advance our understanding of organogenesis or embryonic development.

Comments for the author

In this manuscript, Peng et al. perform single cell RNA-sequencing in Drosophila embryos during two periods of embryogenesis. The single cell RNA-sequencing data is of high quality and will be of interest as a resource to the greater Drosophila community. The authors highlight the sequencing data for three separate cell populations, the salivary glands, the trachea, and the germline. Using GO-term analysis, the authors find that both the salivary glands and the trachea are enriched for GO-terms that are consistent with their function.
They further validate their sequencing data by cross-referencing the BDGP in situ hybridization database to confirm expression of TFs identified in their scRNA-seq data. Additionally, the authors subcluster the putative germ cell cluster and identify six subclusters, including two subclusters (unknown 1 and unknown 2) that occupy a separate pseudotime trajectory from the other 3. While the authors suggest that unknown cluster 1 may be GCs destined to differentiate and not become GSCs in the adult, and unknown cluster 2 could be male GCs that will eventually differentiate into spermatogonia, we find that there is insufficient evidence in the data presented to substantiate either of those claims. The authors then look at the matrisome and reveal from their sequencing that two insulin-like proteins (Ilp4 and Ilp6) are unexpectedly highly expressed in embryonic hemocytes, however additional validation is needed to support this claim. Finally, the authors compare the transcriptomes of five embryonic cell lines with their scRNA-seq data and determine that four out of five of the lines are most closely related to plasmatocytes. Overall Peng et al. has generated a helpful scRNA-seq resource of the drosophila embryo at late stages of development however significant experimental revisions are necessary to support some of the claims made in this manuscript. While this manuscript represents a quality scRNA-seq data set, the findings presented here do not significantly advance our understanding of organogenesis or embryonic development.

Major Points:

1. The authors validate their scRNA-seq by referencing the BDGP in situ hybridization database. In many of the figures, including Fig. 3F, the authors use images directly from the BDGP webpage as figures in this manuscript. Instead, it may be more appropriate to just site the BDGP database rather than using images from the database as figures.

2. Are there any unique markers for GC unknown cluster 1 and 2? If so, can these candidate markers be validated by in situs to help determine the identity of the unknown clusters?

© 2023. Published by The Company of Biologists under the terms of the Creative Commons Attribution License (https://creativecommons.org/licenses/by/4.0/).

a. Is it possible that GC clusters unknown 1 and unknown 2 represent somatic gonadal precursors? Can the authors probe for expression of SGP genes (such as cli/eya, Abd-A/B, TJ) in these clusters?

b. The authors suggest that cluster unknown 2 could be male GCs that will eventually differentiate into spermatogonia, however the two transcripts (CG6701 and Pp2C1) that the authors use as examples of male-specific expression are not male specific. CG6701 and Pp2C1 are found in the female germline (see PMIDs 35239393, 33159074, and 34389661), and are upregulated in spermatids rather than spermatogonia (Raz et al.), though they are also found in spermatogonia. Instead, expression of stg is the best marker for spermatogonia in the Raz et al. paper, though expression of stg is not sex-specific. In addition, the gene otu which the authors describe as differentially expressed in the female germline stem cells, is also expressed in the male germline stem cells (Raz et al.). If the authors suspect that cluster unknown 2 encompasses cells that are destined to be spermatogonia, they should look for expression of genes that reside on the Y chromosome, such as FDY, to confirm that the cluster contains male germ cells rather than female.

c. The authors propose that unknown cluster 1 includes GCs “destined to differentiate and not become germline stem cells”, however it is not clear what the authors mean by “differentiate”. Primordial germ cells at this stage are not differentiating, and will not begin differentiating until after they have transitioned to germline stem cells in the larval gonad.

3. The authors state a role for proteolysis in the establishment of germline stem cell fates, however this claim is not substantiated in the data. The only evidence the authors provide is the enrichment of GO terms associated with proteolysis in the stage 10-12 dataset. Additional experimental evidence is necessary to validate this claim.

4. The authors unexpectedly find that Ilp4/6 are expressed most highly in the embryonic hemocytes. Can this finding be validated by in situ hybridization or immunofluorescence?

5. Figure S11C should be used instead of Fig. 7A, and a more accurate description of the similarities between each cell line and various embryonic cells is necessary in the text. For example, in Fig. S11C, S3 cells are very similar to germ cells, gut endoderm cells, and fat body cells, as well as plasmatocytes. From this analysis are plasmatocytes also similar to germ cells, gut endoderm cells, and fat body cells?

Minor Points:

1. More thorough methods for each gene set enrichment analysis should be added. Specifically, GSEA is highly sensitive to setting the correct enrichment background/reference dataset.

Specific methods should be detailed on how reference datasets were picked/identified for each GSEA.

2. The authors describe two timepoints in their scRNA-seq, each with a single replicate. However, in the later timepoint (stages 13-16), the replicates seem to be quite different, in terms of developmental time. This discrepancy should be described in the text.

3. “Pp2C1” is written as “Pg2C1” in the results section text.

4. The authors cite Raz et al. to suggest that GC differentiation into spermatogonia commences at the end of embryogenesis. This is incorrectly cited and an appropriate citation for this should be added.

5. The authors suggest that the proximity of different clusters in the UMAPs for both collection periods reflects the germ layer of origin (Fig. 1B, D). However, UMAP proximity is not a good measure for how similar/different clusters are, only that they are different. Instead, the authors can create a UMAP with only three clusters (restrict Seurat into separating all cells into just three groups), and confirm that those three groups reflect all cells originating from either the mesoderm, endoderm, or ectoderm.

© 2023. Published by The Company of Biologists under the terms of the Creative Commons Attribution License (https://creativecommons.org/licenses/by/4.0/).

6. Some of the figures, such as S11A&C and S12, are difficult to read due to their low resolution.

7. A more detailed methods section describing exactly how long embryos were collected and aged, would be helpful.

First revision

Author response to reviewers' comments

Reviewer 1 Advance Summary and Potential Significance to Field:
In this manuscript the authors present a single cell RNA-sequencing study covering broadly the time of organogenesis in Drosophila melanogaster. They sample two timepoint, stages 10-12 when organ primordia on average are specified, and stage 13-16, when morphogenesis of many tissues has neared its end and functioning begins.

Following on from scRNAseq the authors now present an in depth analysis of the data, focusing particularly on a subset of tissues, salivary glands, tracheae and germ cells, as well as the matrisome, the collective group of everything secreted in connection with extracellular matrix, and they suggest the origin of several commonly used tissue Drosophila culture cell lines.

The sequencing analysis is based on large numbers of cells and good read depth and the analysis of the data is mostly very thorough. Apart from stainings (in situ and protein) to illustrate the development of salivary glands, tracheal system and germline all analysis and inference is based on the sequencing data and publicly available in situ analyses (at BDGP).

The publicly available sequencing data provided here, together with this initial analysis will provide a great resource to the Drosophila community. As the authors discuss, there are thus far only few scRNAseq studies of Drosophila embryogenesis, and apart from one they tend to focus on specific stages or tissues, hence this global analysis is an important addition.

Reviewer 1 Comments for the Author:
In this manuscript the authors present a single cell RNA-sequencing study covering broadly the time of organogenesis in Drosophila melanogaster. They sample two timepoint, stages 10-12 when organ primordia on average are specified, and stage 13-16, when morphogenesis of many tissues has neared its end and functioning begins.

Following on from scRNAseq the authors now present an in depth analysis of the data, focusing particularly on a subset of tissues, salivary glands, tracheae and germ cells, as well as the matrisome, the collective group of everything secreted in connection with extracellular matrix, and they suggest the origin of several commonly used tissue Drosophila culture cell lines.

The sequencing analysis is based on large numbers of cells and good read depth and the analysis of the data is mostly very thorough. Apart from stainings (in situ and protein) to illustrate the development of salivary glands, tracheal system and germline all analysis and inference is based on the sequencing data and publicly available in situ analyses (at BDGP).

The publicly available sequencing data provided here, together with this initial analysis will provide a great resource to the Drosophila community. As the authors discuss, there are thus far only few scRNAseq studies of Drosophila embryogenesis, and apart from one they tend to focus on specific stages or tissues, hence this global analysis is an important addition. But it is really a resource, and so should be published as such, under the ‘Techniques and Resources’ section rather than as a research article as it currently seems to be. For a research article, more follow up analyses with further experiments, rather than comparison to and analysis of published data, should have been provided.

© 2023. Published by The Company of Biologists under the terms of the Creative Commons Attribution License (https://creativecommons.org/licenses/by/4.0/).

We agree that this is an important resource and we considered simply publishing the paper as such including only an analysis of the quality of the data relative to existing datasets; it seemed reasonable, nonetheless, that having generated this resource that we share some of the applications and insight it has provided regarding the analysis of a subset of tissues, processes, and cell lines. We did follow up with BDGP resulting in some corrections to the database and the addition of genes newly discovered to be expressed in a subset of tissues.

Below I will discuss some general comments about this study, as well as detailed comments to individual parts of the analysis and presentation.

*) At times, sections of this manuscript read rather like a large review on the different tissues and processes analysed, this is true for the introduction (sections on page 4 and 5) as well as the discussion (page 24/25: these pages have a very long discussion of GC knowledge and studies that bears little relation to the manuscript here and would be better in a GC review, this should be condensed).

We agree that several of the sections were inappropriately long; these sections have been shortened.

*) Generally, some conclusions appear overstated, given that all is based on scRNAseq and published in situ images. At times, as pointed out in the detailed comments, conclusion should be phrased more cautiously, especially where other studies and data are criticized and stated to be incorrect, but without the authors here actually providing the evidence.

We have rephrased our findings more cautiously, especially where they conflict with previous work.

*) A general comments about the staging of batches and their relative position along developmental time as well as pseudotime analysis:

Looking at the batches overlaid onto the UMAP of the salivary gland cells as shown in Suppl. Fig.2A and the pseudotime plot in S2B, both panels clearly indicate that the batches mostly split into two groups of either early or late cells, with a small amount of overlap provided by batch 2 of the stage 13-16 embryos. If looked at in a third dimension, is the stage 13-16/rep2 separate from the two early batches? Panel B would suggest possibly yes.

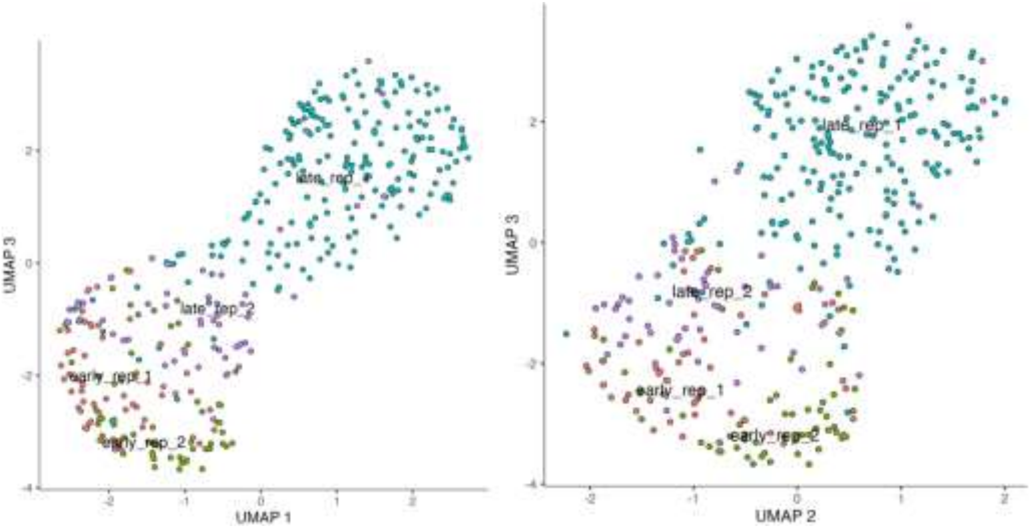

Looking at the third dimension, we think that there is a slight separation between stage 13-16 batch 2 and the early stage data. Consistent with just looking at the data using UMAP_1 and UMAP_2, stage 13-16 batch 2 appears to be in the middle between stage 13-16 batch 1 and stage 10-12 data.

I am unsure of for the analysis of rather few cells here, the pseudotime adds any value beyond the positioning along developmental time that the natural separation of the batches already provides.

© 2023. Published by The Company of Biologists under the terms of the Creative Commons Attribution License (https://creativecommons.org/licenses/by/4.0/).

The review raises a good point regarding the value of the pseudotime. In our perspective, the pseudotime analysis adds two pieces of information. 1) as mentioned previously, it allows us to see the relative positioning of the cells along a developmental trajectory. 2) It gave us a quantitative estimate of how early stage 13-16 batch 2 is relative to stage 13-16 batch 1.

This seems evident in panels S2C and that basically only show a split along batches and thus what is expressed in early cells and what in late cells. Everything seems to nearly come on at the same time. It might make more sense to focus a pseudotime, if at all here, on either early or late batches individually, but that again might not be possible due to the sparsity of cells obtained for the salivary glands.

We thank reviewer for the comments. The main purpose of S3C (previously S2C) is to demonstrate the dynamic activity of these genes even if they are separated strongly by early/late batches. Unfortunately, there are a couple of challenges in running pseudotime on just early or late cells: 1) there are too few cells to reliably construct the pseudotime as suggested by the reviewer. 2) In the tool monocle3, it would be difficult to reliably select the root cells for only early or late batches. When both early and late cells are combined, we select the root cells by locating regions with the highest proportion of stage 10-12 cells.

Following from this, I wonder whether the authors visually screened the embryos submitted for sequencing before dissociation of have matching IF images? It seems surprising that two batches that were meant to be identical (especially looking at Suppl. Table 1), i.e. the two late batches, were so different in their actual developmental staging.

The differences in developmental staging for our late collections was also surprising to us. Our best explanation is that late samples were collected and aged at very different times of the year and RT was slightly different (w/ and w/o air conditioning). We did visually inspect every collection to make sure all embryos were in the appropriate stage range. We do not have IF images of the samples because we were processing the embryos as quickly as possible and we do not have cameras attached to our dissecting scopes. The pseudotime analysis was critical for providing a timeline for all the samples we sequenced - and that timeline seems to be consistent across tissues, even though the pseudotime analyses were done separately.

Detailed comments on introduction, results and discussion:

1) results page 7: In the first paragraph here the number of cell type clusters identified is listed for the two different time points sampled. The deduction of cell clusters and identities should be better explained and shown. Table 2 is just a very long list, it might be useful to split it into the different tissues for easier accessibility, especially if this is a resource, and also to have the column labels fixed floating at the top rather than part of the table that disappears when you scroll.

We thank the reviewer for the suggestions. We have made the adjustment to freeze the top row of Supplementary Table 2. Furthermore, we have added cell type labels to the top of each cluster in Supplementary Table 2. But, we prefer to keep this as a single searchable table instead of multiple tables for users for several reasons. For example, if one wants to know the tissues that express their favorite gene, this table will reveal every tissue just by typing the gene name into the search bar. If we separate the data out as proposed, the person would have to search between 35 and 36 separate tables. For the reader interested in only a single cell type, it is easy to copy paste only that data into a usable table.

We have also made Supplementary Table 3 which contains the top few diagnostic markers that were among the ones used to annotate the cell types for each cluster along with the full gene name, flybase ID and BDGP tissue labels.

For each of the subsequent more detailed sequencing data analysis and conclusions drawn from the data for the salivary glands, tracheae, germline, it would be good to state what the most important, highest differentially expressed markers were that identified these tissues or rather their precursors especially at the early stages. The precursor populations are likely already to some extent non-homogeneous based on the vast literature that is already out there for these

© 2023. Published by The Company of Biologists under the terms of the Creative Commons Attribution License (https://creativecommons.org/licenses/by/4.0/).

tissues and their development. Is for instance Scr or fkh expressed in all cells of the (early) salivary gland cluster defined, is trh or FGFR in all the cells analysed as the tracheal cluster, is vasa etc. in all the ones analysed as germ cells etc? The author do not show a single overlay onto the UMAP of expression of some of their top marker genes for any cluster generated for early and late timepoints. This would be very useful to provide, so readers can judge how specific expression of these marker genes is, and it would also be a good part of a resource. The paper already has a vast number of supplemental figures, so adding these can't hurt.

Thank you, this is important to clarify. We have made two changes to address the issues that are raised in the above discussion. Supplementary Table 3 includes the marker genes used for each tissue. We now also include UMAPs with some of the top marker genes for the tissues we analyzed in detail (SG, trachea, GCs) in Fig S2, S4, S7.

2) Salivary glands analysis, page 8:

The authors draw a lot of conclusions from the analysis of rather a very small number of cells here. How sure are they that representative cells for possibly different regions of the tissue precursor or forming tissue were captured here? 114 and 271 cells respectively appears small.

As is now discussed in the SG section, we did recover fewer SG cells than expected based on the numbers. Given the relative homogeneity of the tissue and depth of sequencing, this is unlikely to be an issue. Moreover, we are detecting genes that from BDGP in situ data are expressed in only a subset of SG genes (sema2A) or only very transiently (e.g. Scr, bnl, hth) or both (Dr, hkb). This is now discussed in the paper.

3) Middle of page 9: the authors state that the downregulation of several salivary transcription factors is a new revelation of their findings, but of the ones they list and show with BDGP in situs, many were published to behave like this previously, including by the authors themselves: hkb was published before, (Myat et al., two papers in 2000), as was eyegone (Jones and Beckendorf, 1998), equally Habermann et al 2003 show that trh is only in duct late, and Scr is beautifully shown in Henderson and Andrew, 2000.

Yes, certainly not all of this is novel. The section on the SG now focuses on how the pseudotime analysis is useful in providing a relative timeline of SG transcription (that applies to all tissues), and how well that timeline fits with what we know from BDGP and our own published data.

The authors also then come back to this in the discussion (page 21), where they discuss how expression differences early versus late might be regulated. I would question what if this difference in mRNA expression is important for function of these transcription factors? From the authors' own publications, it looks as if at the protein level, at least Fkh and CrebA remain strongly present throughout. Could you comment on this?

Yes, we agree this warrants more discussion; this kind of analysis can be a little misleading and should have been explained more clearly. scRNAseq does not capture every single transcript expressed in every cell as discussed in "Concluding remarks" on Page 26. If a gene is abundantly expressed in a given cell type then it is likely to be seen in 100% of those cells, but as the relative abundance goes down (as would the abundance of a transcription factor as it begins to upregulate expression of its downstream targets), the chances of missing the transcript for that TF in any given cell goes up. Thus, we think the data reflect not only genes whose expression is shut off at late stages but also that the abundance of TF transcripts relative to all other transcripts is lower in the more differentiated states.

4) Page 11, tracheal analysis, cross correlation analysis:

This cross correlation analysis is not well explained here, in the figure legend or the methods (where it is only said that this and this tool is used). I find it impossible to tell if this analysis, based on pseudotime (being is a fairly subjective way of analysis), with production of fairly similar albeit not identical curves reflecting expression of many different genes in each functional group, can really tell you anything about temporal staggering and roles of different functional gene sets.

© 2023. Published by The Company of Biologists under the terms of the Creative Commons Attribution License (https://creativecommons.org/licenses/by/4.0/).

Please explain more here, so the reader can judge how meaningful this analysis is.

Cross-correlation is a technique of measuring temporal association between two time-series signals. We have written more detailed explanations of cross-correlations on the results section in Page 12 and more detailed methods Page 30.

Following on from this, the concluding statement says:

‘Altogether, our analysis of the tracheal organogenetic transcriptome reveals the subtle dynamics that underlie the regulatory signature of growth…’.

This is an overstatement and should be phrased more cautiously. Without further follow-on experimental analysis, this is based on pseudotime and cross correlation and in my view can therefore only suggest such dynamics rather than ‘reveal’ it.

Yes, we agree. We have changed the text accordingly.

5) Page 12, GC analysis: as with the two previous tissues, it would be worth stating here what the top genes were that the identification of the GCs was based on in the initial analysis.

We agree and have indicated the key marker genes that identified the GC clusters in Tables S3 and UMAPs of some of these marker genes are now included in the Fig S7.

6) Page 16, conclusion:

What about matrisome expression and ECM secretion by other cells than the plasmatocytes you mostly focus on? The data in Figure 7 clearly show expression also in other tissues/cells, as is also clear from the literature, so this should at least be mentioned.

We now mention the other tissues that express key components of the ECM in our results section (Page 15 – 18).

7) Discussion page 22 bottom:

The authors conclude from their expression analysis and published data that expression of e.g. laminin in plasmatocytes must be carefully spatially coordinated with for instance trachea. My understanding is that for many tissues in the embryo, ECM deposition is controlled by expression of integrins that then capture the hemocyte secreted ECM, thus regulation of integrin expression in the tracheal cell might be all that is required rather than complex spatial coordination?

Yes, the timely expression and trafficking of integrins and other laminin binding proteins to the basal tracheal surface is likely critical - as would be the timely secretion of the ECM components. We have made the additional changes on Page 23.

8) Discussion, page 223 bottom:

The authors compare their inference from GC expression data to published studies and disagree with those studies’ conclusions.

If the authors want to contrast their findings to a previously published study, shouldn't their data be shown, rather than' data not shown'? Also, what are the technical differences between the two analyses? Number of cells sequenced? Read depth? It would be important to comment on this.

The quality of the data generated by Li et al (2021) is quite high – many cells were sequenced for their unsexed (3810 Vasa-GFP positive), male (7222 cells from Sxl-GFP negative embryos) and female (11001 cells from Sxl-GFP positive) data following quality control, and they have good read depth (between 22,241 and 33,487 reads per cell). To test whether sex differences could be the basis for the separation of clusters in our data, we compared ratios of relative gene expression for the two early clusters, the split in the single late cluster and between what we call “unknown 1” versus the “main trajectory” and “unknown 2” versus the “main trajectory”.

© 2023. Published by The Company of Biologists under the terms of the Creative Commons Attribution License (https://creativecommons.org/licenses/by/4.0/).

Although we clearly see that some clusters have higher or lower X chromosome gene expression, they also have corresponding changes in the levels of autosomal gene expression. Thus, we find no evidence of dosage compensation differences among the clusters and thus no support for the hypothesis that, based on dosage compensation, the different clusters in our data correspond to male versus female cells. We also examined the expression profiles of the top 25 sex marker genes described in Figure 5 of Li et al in our data. This analysis also fails to support sex differences they detected as being the basis for the different GC clusters found in our data. We now include the expression data comparisons with respect to clusters and chromosomes as a table in the paper. We have included the results of chromosome ratio comparisons (Fig S35A, S35B), male and female marker genes from sex samples from Li et al, 2021 (Fig S35C, S35D), and male and female marker genes from unsex samples from Li et al, 2021 (Fig S36).

As suggested by reviewer 3, we had already looked for differential expression of Y-encoded genes since this would have been one clear way of knowing which cells were male and which were female. Unfortunately, none of those genes are sufficiently expressed in embryos.

A main difference between our data and that of Li et al., 2021 is that their samples include much earlier cells than ours. Their unsexed sample includes 50% germ cells from 0 - 4 hour embryos and 50% cells from 4 - 8 hour embryos. Our "early" stage 10 - 12 data should overlap only with that from the older cells in their collection. Thus, the differences in X chromosome versus autosome gene expression they observed could be from the younger germ cells, as one might anticipate if dosage compensation in GCs occurs along the same timeline as in somatic cells. We have added additional discussions on Page 24.

Also, first sentence on page 24 'Finding multiple distinct GC populations in our data…' This should be phrased more cautiously, i.e. 'finding multiple suspected or predicted distinct GC populations...' until there is further corroborating data such as in situs or IF of some of the differentially expressed genes actually labeling physically distinct cell populations.

We have made the changes on Page 25.

9) Discussion, page 26, just above concluding remarks:

The authors disagree with the conclusions of another study, suggesting a Gal4 driver expressed in more cells than assumed led to aberrant conclusions.

Again, this should be phrased more cautiously or suggestive if the authors do not actually show any analysis of srp-Gal4 expression in apodemes but only speculate that this is the underlying cause of the discrepancy!

This observation has been phrased more cautiously. We have made the appropriate adjustments to the text on Page 26.

Detailed comments on methods:

10) Page 29, the authors state ' The cluster marker genes were matched with a table of marker genes of various cell types curated from the BDGP database'' :

Where can this table of marker genes be found and how was it curated? The citation is 'Table S2', but this is where the results are shown, not this comparative table.

We now provide two tables of marker genes in comparisons with BDGP cell type labels. The tables can be found in Table S3.

Detailed comments on Figures and Figure Legends:

11) As a general comment, these figure legends are rather sparse, more information on plots, statistics, what individual panels show etc etc should be provided!

We have adjusted the figure and supplementary figure legends.

© 2023. Published by The Company of Biologists under the terms of the Creative Commons Attribution License (https://creativecommons.org/licenses/by/4.0/).

12) Figure 3E and all related dot plots:

This plot and all subsequent related ones need more explanation, citing what the expression and percentage (percentage of cells in cluster expressing gene?) refer to and therefore imply.

We have added additional explanations of the dot plots in Figure and Supplementary figure legends. Here is an example text that was used for Figure 5: "The size of the dot represents the percentage of cells in the GC subtype in which the gene is detected and the color of the dot represents the mean expression of the gene in the GC subtype."

13) Figure 3A: In this as in all similar figures/panels (apart form in Figure 1 in the total UMAPs for earlier and later stages) the small coloured dots explaining identity of clusters are too small to be able to identify the colour without having to zoom in, especially in figures like the GC analysis, where there are many clusters and the colours are not that distinct (was a colour-blind friendly colour code taken into account?), can these please be increased in size?

We thank the reviewer for the comments. We have made adjustments in Fig 3A, 3B, 4A, 4B, 5A, 5B, S3A, S5A, S5B, S8A, S8B. The main color pallet is from RColorbrewer Set 2, which should be a colorblind friendly pallet.

Detailed comments on Figures:

14) Figure 2D and Figure 3E: how do these GO term analyses compare to each other for the salivary glands? Looking at the scale in both sections, 'Golgi vesicle transport' at stage 10-12 in Fig. 2D has a change of ~5, whereas the same GO term in Fig. 3E only has a value of ~1.7, why would that be?

And for stage 13-16 embryos salivary gland, cytoplasmic translation in 2D has a value of ~5 whereas 3E has a value of nearly 30 for the same GO term. Why is that?

Figure 2D is comparing the relative enrichment of GO terms in the tissue in question relative to all other tissues at that stage. Figure 3CD, Figure 4C and Figure 5C are the relative enrichment <u>within</u> the tissue comparing the different subclusters. The differences in the -log(adjusted p-value) could be due to the differences in the types of comparisons that we are doing.

15) Dot plots in 3E, 5D and similar:

I find these plots very unintuitive, what about showing the expression of e.g. the four genes in 5D overlaid on the UMAP shown in panel 5A? Or at least show both?

For large scale analyses such as the matrisome these plots make more sense, but for smaller comparison UMAP expression maps are much clearer.

Yes, we agree. We have done so in Fig S9A.

16) Supplementary Figure 3:

Back to general comments, the colour code for the clusters in A is near impossible to match to the plot as the explanatory dots are too small and the colours too similar.

This has been fixed in Fig S5A.

The cluster in the center representing tip cells when looking at the batch plot in B seems to be composed of cells from both the stage 10-12/rep1 and the stage13-16/rep2 cluster. Is there a time progression within this cluster? The two sources seem to very clearly split into two, so what is the significance of this?

Yes, clearly the "younger" tip cells segregate from the "older" tip cells; something we would expect since gene expression profiles change over time in the other tracheal cell populations as well. We compared expression data from the two populations of tip cells to find the top DE

© 2023. Published by The Company of Biologists under the terms of the Creative Commons Attribution License (https://creativecommons.org/licenses/by/4.0/).

genes (see Fig. S5D); most of the genes that emerge from this analysis are CGs or other genes whose functions in flies have not been explored. The exceptions would include early tip cell expression of Brd, which regulates Notch signaling and could facilitate Notch-dependent fate choices among tip cells (Llimargas, 1999), and verm and serp expression in late tip cells, which we might expect based on their roles in late tube elongation/growth. We have included this results in Fig S5D.

Panel C: This analysis very clearly shows that the repeats of the later batches are very different in timing, so the pseuotime plots presented in Suppl Fig 4 really just seem to represent that. Only the chitin synthesis show any real progression.

We agree.

17) Supplementary Figure 5:

I am confused by the pseudotime plot in C: why are the cells ordered like this here, with stage 13-16/rep1 appearing to span later developmental time than rep2, when all the previous analyses of salivary gland and tracheae suggested that stage 13-16/rep 2 covers earlier cells than stage 13-16 /rep1? How can this be?

We thank the reviewer for this observation. The reason why stage 13-16/rep2 appears to be later than stage 13-16/rep1 (contradictory to what we observed in SG and Tr) is because in the original violin plot, we also included cells from the other trajectory (right- unknown 1 and unknown 2). When we removed those cells from that trajectory in the violin plot, we can see that it is largely consistent with what we have previously observed (Fig S8A). We have also made it clear that the violin plot only contains the cells from main trajectory (Early Germ Cells, Interm. Germ Cells 1, Interm. Germ Cells 2, Late Germ Cells) in the figure legend.

Doesn't the UMAP in B rather suggest that there is a split in the late (green) cluster into different cells?

Yes, based on the UMAP in original Fig S5B (now Fig S8B), there is a split in the stage 13-16 rep 1 cells (green) into different cells (unknown 1 and unknown 2). Due to how the pseudotime was inferred, many of the cells in unknown 1 and unknown 2 have small pseudotime ordering, and that is why in the original Fig S5C, we see that the stage 13-16 rep 1 has an earlier pseudotime than stage 13-16 rep 2. However, if we remove the unknown 1 and unknow 2 cells, then the ordering of the batches match with what we observed in trachea and salivary gland (now Fig S8C).

18) Supplementary Figure 6:

Panel D: the statistical analysis symbols are really hard to see on the background used, please change this.

These have been enlarged in Fig S9D.

19) Supplementary Figure 8:

What does the first cluster labelled as ‘artefact’ represent ⁇

This is a category the authors included in the paper that described the adult single cell data for which they could not assign an identity. Based on the original paper, the ‘artefact’ cluster represents a cluster of cells that expresses nearly all genes as described in (Li et al, 2022) (DOI: 10.1126/science.abk2432). The cluster was retained as we did not do any processing of the scRNA-seq data downloaded directly from https://flycellatlas.org/.

Panel B: the colour scale used for the expression overlay on the UMAP is not ideal. The purple representing no expression is the most obvious colour, with the pale green and even the yellow indicating expression very hard to see, especially where it intersects with purple cells. Non-expressing cells should ideally be a pale colour with expression marked by increasing intensity of

© 2023. Published by The Company of Biologists under the terms of the Creative Commons Attribution License (https://creativecommons.org/licenses/by/4.0/).

a bright colour.

This has been changed so that the highest intensity dots correspond to cells with the highest levels of Ilp6 expression. Please see Fig S11B.

19) Supplementary Figure 12:

Panel B, right hand side: why in this plot does the salivary gland from Calderon highlight all cell types in the stage 13-16 samples, very different to the stage 10-12 sample above?

The main purpose of this exercise is to find the degree of agreement between our cell typing results and those from Calderon et al and Seroka et al. We used a computational tool called SingleCellNet to train on labelled single-cell data from Calderon and Seroka, and applied to our data. The idea is if there is high agreement between the two cell typing results, we would expect to see a diagonal line in Fig S15 suggesting that the cell types labelled by us are also similar to those cells from other studies.

In the case of the late data, the plot indicates that most of our cell types are more or less classified as cells that are labelled as salivary glands from Calderon in stage 13-16. This does not necessarily mean that biologically the cells are transcriptionally similar to salivary gland, there could be multiple technical factors that could confound this analysis, most likely the drastic difference in read depth between Calderon's data and ours, leading Calderon's data to capture most abundant genes - actin, ribosomal proteins, etc that other cell types cannot be distinguished from the SG.

20) Supplementary Figure 29 and similar:

In these plots, I cannot see what is meant to be shown in A-C, is anything highlighted here? And in panel D, E , where the legend shows two colours for different clusters/cell types it is impossible to distinguish these two colours, please explain, annotate better and use a different colour scheme.

There is nothing highlighted there. The main point we want to demonstrate here is that there are no cells that are transcriptionally similar to that of Apodemes, Salivary gland, and Malpighian tubules in Seroka et al's stage 12 data.

In panel D and E, there are rare cells that were not originally labelled in the Seroka et al and Calderon et al paper that were classified as such. Unfortunately, these rare cell types are very small in number in the dataset, we tried to increase visibility by including an arrow pointing to the location of the cells. We also used a red arrow in Fig S32-34 hoping to make it easier for the readers to identified classified cells.

Fig. S30/31 have the same colour scheme problem, he colour that is supposed to highlight expression needs to be clearly different and brighter or darker than the background colour to make this visible.

We have made the appropriate changes in Fig S32-34.

Little bits:

1) Page 4 bottom , introduction: '…the discoveries […] accelerated the process of discovering key players.' This sentence does not make much sense to me, what do the authors mean?

This sentence has been edited.

2) Page 10, end of first paragraph: 'contiguous' (i.e. adjacent) makes no sense here, you must mean continuous.

No, I think we mean contiguous.

11) methods page30 top, pseudotime analysis:
Various typos or grammatical errors:'… was used perform…', 'pseodtime'

© 2023. Published by The Company of Biologists under the terms of the Creative Commons Attribution License (https://creativecommons.org/licenses/by/4.0/).

These typos and any others we have discovered have been fixed.

******* Reviewer 2 Advance Summary and Potential Significance to Field:**

Overall a great paper producing a very useful dataset of embryo scRNAseq, followed by an excellent demonstration of how this can be combined with BDGP in situs for the analysis of organogenesis. The research performed and its presentation are all at the top standard. I am confident it will be of interest to the readers of Development.

Reviewer 2 Comments for the Author:
Minor revisions that must be done.
1. Data availability; although putting the raw data in GEO is useful, the authors should also upload it to scRNA expression atlas (https://www.ebi.ac.uk/gxa/sc/home), for several reasons. It will permit researchers with less bioinformatic experience the tools to explore the data. By this database analysing the raw data using the same pipeline, it will also aid comparison with the other published data sets. It will also gain a dataset report in FlyBase via this route.

We have contacted Dr. Liora Vilmovsky at the Single Cell Expression Atlas. Once the paper is public, we will share it with them and they will upload the data if they decide that the data is suitable for the Atlas.

2. It would be a great help for future curation of this data if the cell types were annotated with the cell type terms from the FlyBase anatomy ontology; assignment of the correct terms could be provided in Supplementary Table 7

We thank the reviewer for the suggestion; however, we think that by forcefully adding FlyBase anatomy ontology terms to the harmonized cell types may create additional confusion. It should be noted that FlyBase anatomy ontology is not entirely consistent. e.g. plasmatocyte is not listed in anatomical terms available through BDGP (hemocytes, lamellocytes and macrophage are listed, but in reality – they should be plasmatocytes). Moreover, plasmatocytes is consistently used in the in-situ descriptions.

Additionally, some of the harmonized cell types are too ambiguous for FlyBase anatomy ontology. For instance, working with the annotation of cell types from Seroka, Calderon and our data, we had to bin foregut and hindgut together for stage 10-12 cell type harmonization because transcriptionally it's difficult to distinguish foregut and hindgut. It would be confusing to forcefully assign that category as either foregut (FBbt:00005379) or hindgut (FBbt:00005384). Another example would be salivary gland labelled cells potentially can take on two FlyBase anatomy ontology salivary gland body primordium (FBbt:00005512) and embryonic/larval salivary gland body (FBbt:00007594).

Overall, we feel the benefit of adding FlyBase anatomy ontology terms (and potentially adding them incorrectly) does not substantially improve the usability of our data.

3. p7
Table S2, the table needs more explanation of the data in each of the columns. It wasnt clear how the list of genes in this table was produced (top 20-50 differentially expressed genes is vague, and the description in the methods is not that much clearer). This is probably the most generally useful data in this paper, in addition to the raw data, but its not very clear what is being shown. Full names of the genes would also be a helpful addition.

We have provided additional details in our methods section "Single cell RNA-seq Processing". We have also provided full gene names for the selected marker genes used to identify cell types in Table S3.

4. p7
" were used to assign 25 distinct cell types (Fig. 1B) from 35 early single-cell clusters (Fig. S1B) and 31 distinct cell types (Fig. 1D) from the 36 late single-cell clusters (Fig. S1D)."

© 2023. Published by The Company of Biologists under the terms of the Creative Commons Attribution License (https://creativecommons.org/licenses/by/4.0/).

I was not able to relate the 25 to 35 or 31 to 36 from these figures. Perhaps a table is needed? or connect them in one supplementary figure?

The tables are now included as panels E and F of Supplemental Figure 1.

5. p7 the statement “the proximity of different clusters in the UMAPs […] reflect the germ layer of origin, e.g., all mesodermal derivatives […] are clustered closely”. That may be true but it is dubious whether this has any significance. It is debatable whether proximity of clusters in UMAP embeddings reflect some biological facts about the clusters or are merely consequences of distortions caused by the embedding algorithm (see a recent preprint from Chari & Pachter for intensive discussion on that issue: http://biorxiv.org/lookup/doi/10.1101/2021.08.25.457696).

This statement has been removed.

6. The fact that muscles undergo fusion during these stages of development needs a mention. For example, does that result in a reduced fraction muscle 'cells' relative to using single nuclear RNA sequencing?

Seems like muscle cells are about ~20% in both stages. We now comment on that in the early part of the results.

7. p13 the last sentence reference to supplementary figure S6C: in figure S6 it is labelled D and there is no C.

This has been repaired.

8. p15 Section on Basement membrane.
There are only 4 major components of the basement membrane, yet only two are analysed in this section. Add the other: two perlecan/trol and Nidogen

These genes have been added.

9. It would also be very useful to compare the basement membrane associated laminin alpha chain LanA to the non-basement membrane one wb.

wb has been added.

10. I find this statement is going a bit on the wrong track "Given that the primary source of Laminins are the plasmatocytes..." I agree its plasmatocytes produce the highest level, but as clearly indicated in the figure, most cells express laminins, in contrast to other basement membrane components, so it’s not so clear that the spatial coordination discussed is required.

Yes, you are correct. The statement has been modified.

11. Supplementary figures S2C, S2D and S4, add a label directly in the figure to indicate that the comparison is across the inferred pseudo time (e.g. with a label on the X axis, accompanied by an arrow), instead of just mentioning in the legend.

This has been done.

12. Add the data shown in Figure 6 and supplementary Figures, 7, 9 and 10 as supplementary tsv files, so readers can explore the data themselves, or replot the data.

We have generated supplementary tables for the matrisome analysis, now shown in the paper as Table S11 and Table S12.

Suggestions for additional minor revisions.

13. I didn’t find the second paragraph of the introduction particularly helpful in framing the value of the research presented in this paper; I think it could be removed without detriment. While it is

© 2023. Published by The Company of Biologists under the terms of the Creative Commons Attribution License (https://creativecommons.org/licenses/by/4.0/).

helpful to introduce the BDGP in situs; the third paragraph could also be shortened.

We would like to keep this flow in the introduction as it provides some context for how recent advances in technology complement historical studies on organogenesis. The text has, nonetheless, been shortened and clarified.

14. Some readers might find it surprising that the tracheal tip cells are similar to axons; so this may merit some further discussion.

We now indicate that this finding is in keeping with models out there suggesting that the process of axon guidance is very similar to cell migration.

******* Reviewer 3 Advance Summary and Potential Significance to Field:**
In this manuscript, Peng et al. perform single cell RNA-sequencing in Drosophila embryos during two periods of embryogenesis. The single cell RNA-sequencing data is of high quality and will be of interest as a resource to the greater Drosophila community. The authors highlight the sequencing data for three separate cell populations, the salivary glands, the trachea, and the germline. Using GO-term analysis, the authors find that both the salivary glands and the trachea are enriched for GO-terms that are consistent with their function.
They further validate their sequencing data by cross-referencing the BDGP in situ hybridization database to confirm expression of TFs identified in their scRNA-seq data. The authors subcluster the putative germ cell cluster and identify six subclusters to which they assign potential functions. Finally, the authors compare the transcriptomes of five embryonic cell lines with their scRNA-seq data and determine that four out of five of the lines are most closely related to plasmatocytes. While this manuscript represents a quality scRNA-seq data set, the findings presented here do not significantly advance our understanding of organogenesis or embryonic development.

Reviewer 3 Comments for the Author:
In this manuscript, Peng et al. perform single cell RNA-sequencing in Drosophila embryos during two periods of embryogenesis. The single cell RNA-sequencing data is of high quality and will be of interest as a resource to the greater Drosophila community. The authors highlight the sequencing data for three separate cell populations, the salivary glands, the trachea, and the germline. Using GO-term analysis, the authors find that both the salivary glands and the trachea are enriched for GO-terms that are consistent with their function.
They further validate their sequencing data by cross-referencing the BDGP in situ hybridization database to confirm expression of TFs identified in their scRNA-seq data. Additionally, the authors subcluster the putative germ cell cluster and identify six subclusters, including two subclusters (unknown 1 and unknown 2) that occupy a separate pseudotime trajectory from the other 3. While the authors suggest that unknown cluster 1 may be GCs destined to differentiate and not become GSCs in the adult, and unknown cluster 2 could be male GCs that will eventually differentiate into spermatogonia, we find that there is insufficient evidence in the data presented to substantiate either of those claims. The authors then look at the matrisome and reveal from their sequencing that two insulin-like proteins (llp4 and llp6) are unexpectedly highly expressed in embryonic hemocytes, however additional validation is needed to support this claim. Finally, the authors compare the transcriptomes of five embryonic cell lines with their scRNA-seq data and determine that four out of five of the lines are most closely related to plasmatocytes. Overall, Peng et al. has generated a helpful scRNA-seq resource of the drosophila embryo at late stages of development, however significant experimental revisions are necessary to support some of the claims made in this manuscript. While this manuscript represents a quality scRNA-seq data set, the findings presented here do not significantly advance our understanding of organogenesis or embryonic development.

Major Points:

1. The authors validate their scRNA-seq by referencing the BDGP in situ hybridization database. In many of the figures, including Fig. 3F, the authors use images directly from the BDGP webpage as figures in this manuscript. Instead, it may be more appropriate to just site the BDGP database rather than using images from the database as figures.

We think it is important to have examples in the actual manuscript as all readers may not want to take the time to go to the website.

© 2023. Published by The Company of Biologists under the terms of the Creative Commons Attribution License (https://creativecommons.org/licenses/by/4.0/).

2. Are there any unique markers for GC unknown cluster 1 and 2? If so, can these candidate markers be validated by in situs to help determine the identity of the unknown clusters?

Yes, in situs with those genes could be done, but may not have the resolution needed. We think it is best to obtain antibodies to differentially expressed genes (if they exist) and do fluorescent immunostaining in combination with a GC marker like VASA. We also think it is important that the data in the paper be accessible to others as soon as possible.

a. Is it possible that GC clusters unknown 1 and unknown 2 represent somatic gonadal precursors? Can the authors probe for expression of SGP genes (such as cli/eya, Abd-A/B, TJ) in these clusters?

We did probe for the SGP genes indicated here as well as for dsx (and other SGP markers) and found the SGPs embedded in a mesodermal cluster. There is no specific enrichment in SGP genes in unknown 1 or unknown 2. It is also worth pointing out that the expression values for SGP genes are low across all germ cell clusters (max expression at ~0.05 for log(mean + 0.01)).

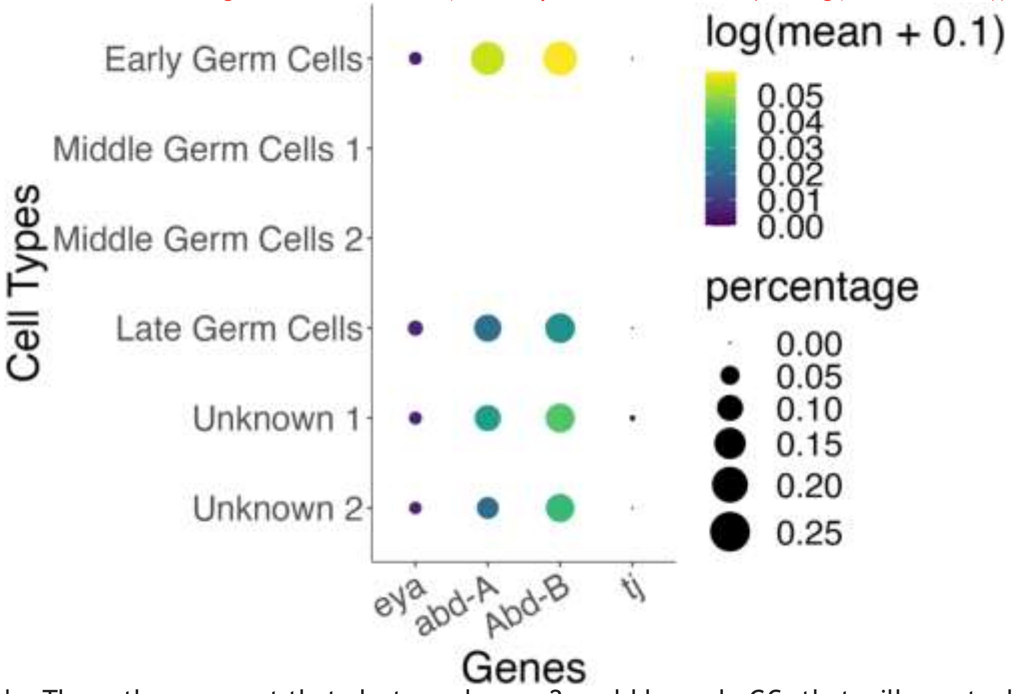

b. The authors suggest that cluster unknown 2 could be male GCs that will eventually differentiate into spermatogonia, however the two transcripts (CG6701 and Pp2C1) that the authors use as examples of male-specific expression are not male specific. CG6701 and Pp2C1 are found in the female germline (see PMIDs 35239393, 33159074, and 34389661), and are upregulated in spermatids rather than spermatogonia (Raz et al.), though they are also found in spermatogonia. Instead, expression of *stg* is the best marker for spermatogonia in the Raz et al. paper, though expression of stg is not sex-specific. In addition, the gene otu, which the authors describe as differentially expressed in the female germline stem cells, is also expressed in the male germline stem cells (Raz et al.). If the authors suspect that cluster unknown 2 encompasses cells that are destined to be spermatogonia, they should look for expression of genes that reside on the Y chromosome, such as FDY, to confirm that the cluster contains male germ cells, rather than female.

Yes, we absolutely agree that looking for Y-encoded genes would be the best approach. We tried that and found that none of the Y encoded genes are expressed in embryos (see below).

© 2023. Published by The Company of Biologists under the terms of the Creative Commons Attribution License (https://creativecommons.org/licenses/by/4.0/).

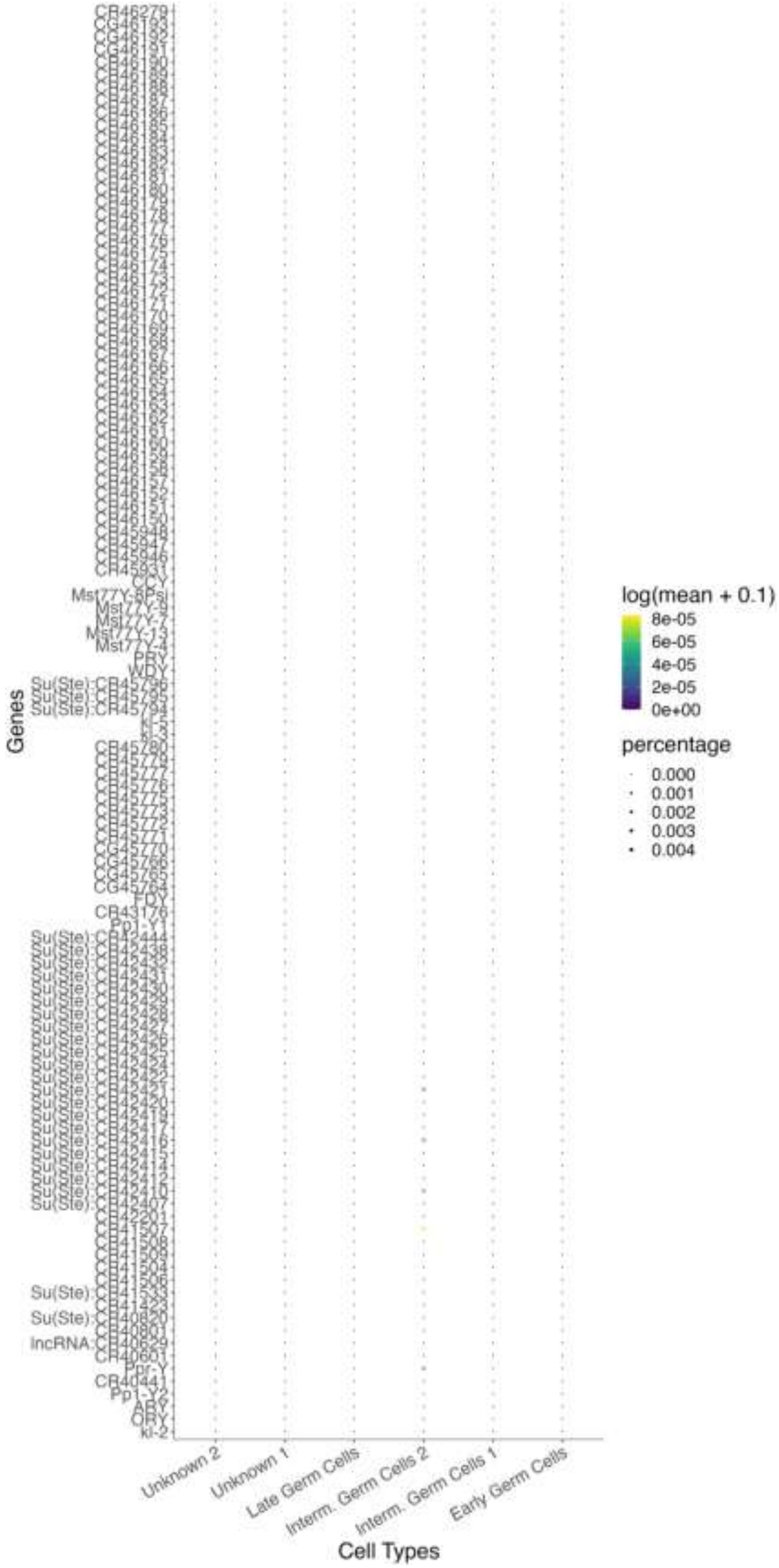

We did not find any noticeable differential expression among the clusters with stg.

© 2023. Published by The Company of Biologists under the terms of the Creative Commons Attribution License (https://creativecommons.org/licenses/by/4.0/).

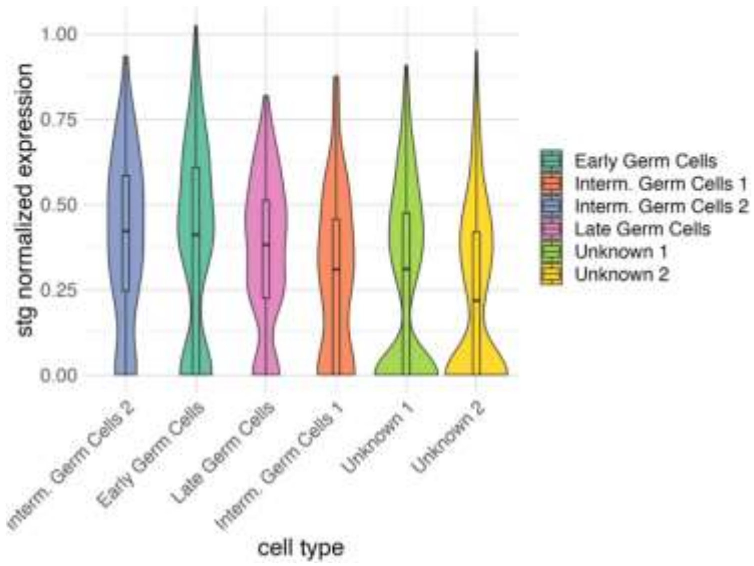

Furthermore, we did not find differential expression among the subtypes with FDY.

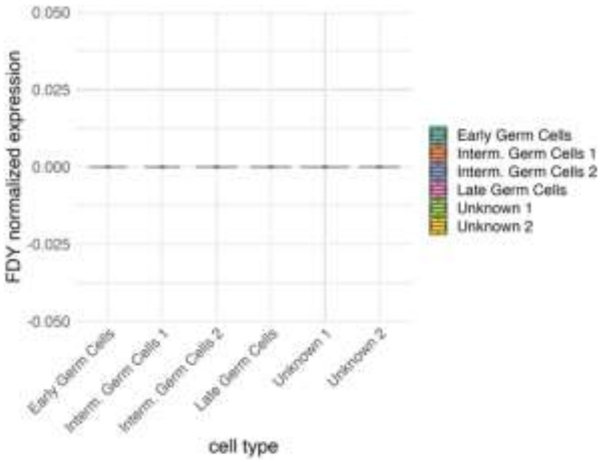

c. The authors propose that unknown cluster 1 includes GCs “destined to differentiate and not become germline stem cells”, however it is not clear what the authors mean by “differentiate”. Primordial germ cells at this stage are not differentiating and will not begin differentiating until after they have transitioned to germline stem cells in the larval gonad.

We propose that these are the cells that will eventually become spermatogonia in male first instar larvae or cystoblasts in females.

3. The authors state a role for proteolysis in the establishment of germline stem cell fates, however this claim is not substantiated in the data. The only evidence the authors provide is the enrichment of GO terms associated with proteolysis in the stage 10-12 dataset. Additional experimental evidence is necessary to validate this claim.

We now simply state that finding these genes suggest a potential role for proteolysis in germline stem cell fates and cite two papers in worms supporting such a role.

4. The authors unexpectedly find that Ilp4/6 are expressed most highly in the embryonic hemocytes. Can this finding be validated by in situ hybridization or immunofluorescence?

We have been working with Sue Celniker at BDGP regarding the SG genes that we did not pick up in our data. This lead to the removal of some of the genes originally on the SG expressed list as well as some modifications in the descriptions (as described in the revised manuscript). She also had the BDGP team do in situs on the set of genes we were most curious about. Ilp4 and llp6 were on that list and data for both genes is now available in BDGP. Ilp4 is clearly expressed in plasmatocytes and Ilp6 is expressed in yolk. The in-situs from BDGP can be found in Fig S37.

© 2023. Published by The Company of Biologists under the terms of the Creative Commons Attribution License (https://creativecommons.org/licenses/by/4.0/).

5. Figure S11C should be used instead of Fig. 7A, and a more accurate description of the similarities between each cell line and various embryonic cells is necessary in the text. For example, in Fig. S11C, S3 cells are very similar to germ cells, gut endoderm cells, and fat body cells, as well as plasmatocytes. From this analysis are plasmatocytes also similar to germ cells, gut endoderm cells, and fat body cells?

The image shown in Figure 7A presents the cell type with the top numerical score in each comparison. The color chart provided in S11C (now Fig S14C) presents a challenge in discerning which cell type scored highest. Therefore, we think that Figure 7A provides the readers a quick and easy information on the most similar cell type, and if the readers want, they could go find the continuous heatmap in Fig S14C.

We also ran the correlational analysis of the plasmatocytes (stage10-12 and stage 13-16) against all the other cell types in the embryos (see figure below). We found that stage 10-12 fat body is decently correlated with stage 10-12 plasmatocytes. Other than that, we don't think plasmatocytes are similar to germ cells or gut endoderm cells.

© 2023. Published by The Company of Biologists under the terms of the Creative Commons Attribution License (https://creativecommons.org/licenses/by/4.0/).

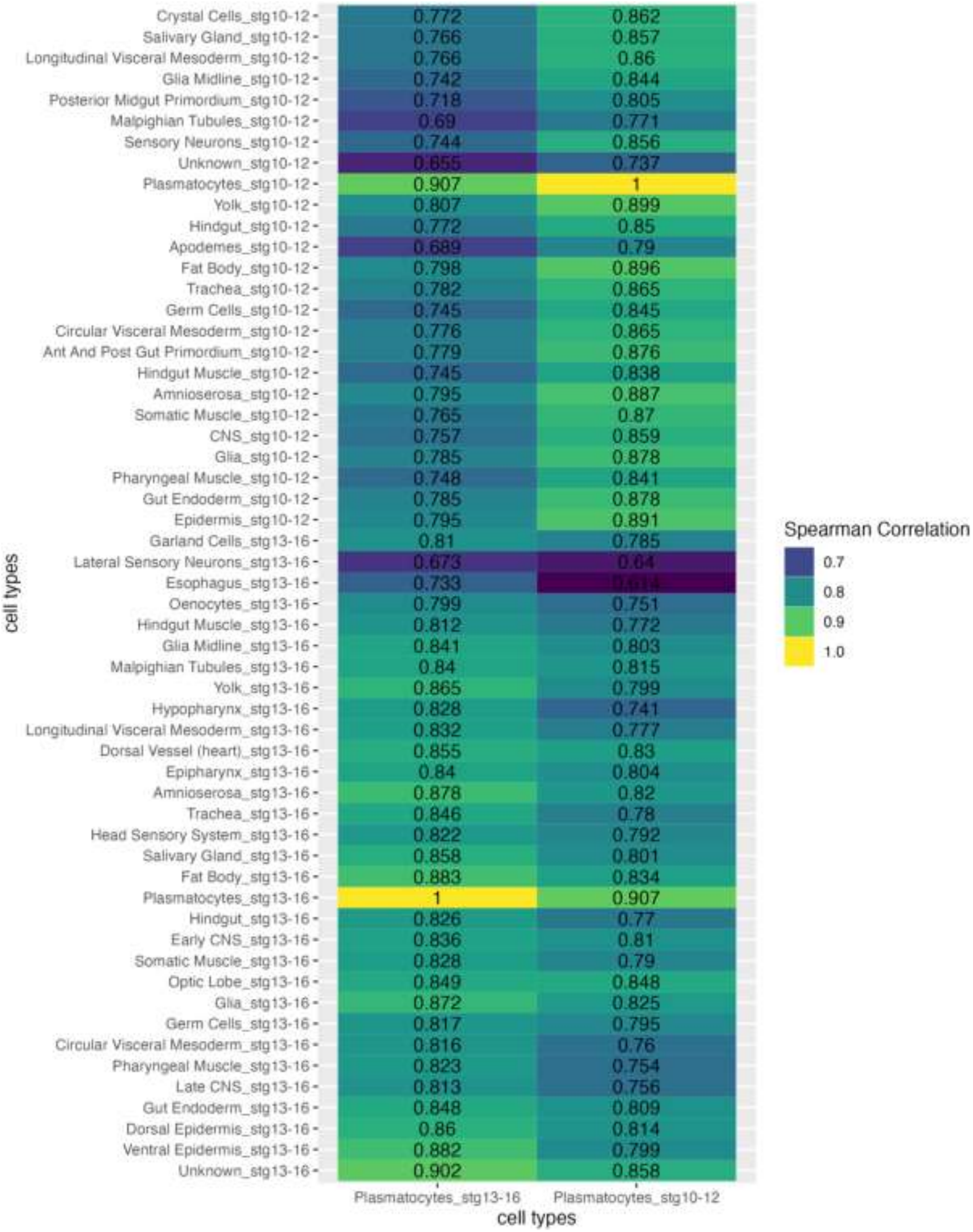

Minor Points:

1. More thorough methods for each gene set enrichment analysis should be added. Specifically, GSEA is highly sensitive to setting the correct enrichment background/reference dataset. Specific methods should be detailed on how reference datasets were picked/identified for each GSEA.

*We have written out the procedural methods in more detail in our methods sections. The R software fgsea (**doi:** https://doi.org/10.1101/060012) was based on an established method (https://doi.org/10.1073/pnas.0506580102). More specifically, in this variant of GSEA, it ranks the logfold change and then calculate enrichment score via Kolmogorov-Smirnov statistics. After*

© 2023. Published by The Company of Biologists under the terms of the Creative Commons Attribution License (https://creativecommons.org/licenses/by/4.0/).

getting the enrichment score, the tool estimates the p-value via permutation test. We have indicated the number of permutations (1000) we used to construct the background for p-value estimation in our method.

2. The authors describe two timepoints in their scRNA-seq, each with a single replicate. However, in the later timepoint (stages 13-16), the replicates seem to be quite different, in terms of developmental time. This discrepancy should be described in the text.

We addressed this issue in the text and provide more details in response to a similar comment from reviewer 1.

3. “Pp2C1” is written as “Pg2C1” in the results section text.

This has been fixed.

4. The authors cite Raz et al. to suggest that GC differentiation into spermatogonia commences at the end of embryogenesis. This is incorrectly cited and an appropriate citation for this should be added.

This has been done.

5. The authors suggest that the proximity of different clusters in the UMAPs for both collection periods reflects the germ layer of origin (Fig. 1B, D). However, UMAP proximity is not a good measure for how similar/different clusters are, only that they are different. Instead, the authors can create a UMAP with only three clusters (restrict Seurat into separating all cells into just three groups) and confirm that those three groups reflect all cells originating from either the mesoderm, endoderm, or ectoderm.

The reviewer is correct and we have removed this comment from the paper.

6. Some of the figures, such as S11A&C and S12, are difficult to read due to their low resolution.

We agree - the low resolution that we have to use to upload the manuscript for review makes the details in some panels very difficult to see. The panels are clearly visible on the originals and should be visible on the high resolution images that will be published.

7. A more detailed methods section describing exactly how long embryos were collected and aged, would be helpful.

This information is now provided in the materials and methods.

Second decision letter

MS ID#: DEVELOP/2023/202097

MS TITLE: Organogenetic transcriptomes of the Drosophila embryo at single cell resolution

AUTHORS: Da Peng, Dorian Jackson, Bianca Palicha, Eric Kernfeld, Nathaniel J Laughner, Ashleigh Shoemaker, Sue Celniker, Rajprasad Loganathan, Patrick Cahan, and Deborah Andrew
ARTICLE TYPE: Research Article

I am happy to tell you that your manuscript has been accepted for publication in Development, pending our standard ethics checks.

Reviewer 1

Advance summary and potential significance to field

© 2023. Published by The Company of Biologists under the terms of the Creative Commons Attribution License (https://creativecommons.org/licenses/by/4.0/).

As before, this is a great resource for the field.

Comments for the author

In this revised version of the paper the authors have addressed all the concerns raised by the reviewers, in particular they have toned down some conclusions, have adjusted figures to make them clearer and have included further panels such as UMAPs to illustrate better the expression of key markers of clusters.

Overall, this manuscript is therefore much improved.

My only remaining minor niggle would be that although the authors say they have taken out some of the weather review or thesis-introduction like detail from he introduction, it is still equally long and only small bits have in fact been removed. I feel there would still be room for improvement.

the figure legends, by contrast, still seem on the rather short side. Are there for instance no statistics associated with plots such as Figure 4 D and E?

And finally, new Suppl Fig S2 E,F do not seem to add anything, at least not as two columns, with both columns in all but one case stating the same information.

Reviewer 2

Advance summary and potential significance to field

as before

Comments for the author

Overall the authors have addressed the points I raised satisfactorily, except for these two.

2. It would be a great help for future curation of this data if the cell types were annotated with the cell type terms from the FlyBase anatomy ontology; assignment of the correct terms could be provided in Supplementary Table 7

Author response: We thank the reviewer for the suggestion; however, we think that by forcefully adding FlyBase anatomy ontology terms to the harmonized cell types may create additional confusion. It should be noted that FlyBase anatomy ontology is not entirely consistent. e.g. plasmatocyte is not listed in anatomical terms available through BDGP (hemocytes, lamellocytes and macrophage are listed, but in reality – they should be plasmatocytes). Moreover, plasmatocytes is consistently used in the in-situ descriptions.

Additionally, some of the harmonized cell types are too ambiguous for FlyBase anatomy ontology. For instance, working with the annotation of cell types from Seroka, Calderon and our data, we had to bin foregut and hindgut together for stage 10-12 cell type harmonization because transcriptionally it’s difficult to distinguish foregut and hindgut. It would be confusing to forcefully assign that category as either foregut (FBbt:00005379) or hindgut (FBbt:00005384). Another example would be salivary gland labelled cells potentially can take on two FlyBase anatomy ontology salivary gland body primordium (FBbt:00005512) and embryonic/larval salivary gland body (FBbt:00007594).

Overall, we feel the benefit of adding FlyBase anatomy ontology terms (and potentially adding them incorrectly) does not substantially improve the usability of our data.

Reviewer response: I am not swayed by this argument. First of all, BDGP is not updated with the latest FlyBase Anatomy Ontology, so that point is not relevant. Secondly, the FlyBase cell type ontology is neither perfect nor written in stone; one of the exciting things about single cell sequencing is the new insights into cell types, and these can be used to update the ontology. So the fact that there are a couple of examples where there is not a matching term does not mean that its

© 2023. Published by The Company of Biologists under the terms of the Creative Commons Attribution License (https://creativecommons.org/licenses/by/4.0/).

not useful to add terms for all the cases were it does match. The authors can also request new cell type ontology terms from FlyBase.

3. p7
Table S2, the table needs more explanation of the data in each of the columns. It wasnt clear how the list of genes in this table was produced (top 20-50 differentially expressed genes is vague, and the description in the methods is not that much clearer). This is probably the most generally useful data in this paper, in addition to the raw data, but its not very clear what is being shown. Full names of the genes would also be a helpful addition.

Author response: We have provided additional details in our methods section “Single cell RNA-seq Processing”. We have also provided full gene names for the selected marker genes used to identify cell types in Table S3.

Reviewer Response: Having read this section of the materials and methods I am none the wiser of what most of the headings are for the supplementary tables. (and what is this supposed to mean in that section of the methods? : "Top significant number of principal components (PCs) was found...") The authors need to add a starting sheet for each table where the non abbreviated title of each column heading is shown and a brief description of the purpose of showing this information.
Examples of the lack of clarity
Table 2- what is the difference between the p_val and p_val_adj?
what are the numbers pct.1 and pct.2 supposed to signify to the reader?
Table 4, are we supposed to guess what ES and NES and leadingEdge mean?
Table 11-what is the difference between the avg.exp and avg.exp.scaled?
what is the purpose of the features.plot column?
etc., etc.

Its great you added full gene names and Flybase gene identifiers in Table 3. Add them to all the tables, there is plenty of room.

Reviewer 3

Advance summary and potential significance to field

In this manuscript, Peng et al. perform single cell RNA-sequencing in Drosophila embryos during two periods of embryogenesis. The single cell RNA-sequencing data is of high quality and will be of interest as a resource to the greater Drosophila community. The authors highlight the sequencing data for three separate cell populations, the salivary glands, the trachea, and the germline. They further validate their sequencing data by cross-referencing the BDGP in situ hybridization database to confirm expression of TFs identified in their scRNA-seq data. In summary, Peng et al. have generated a helpful scRNA-seq resource of the Drosophila embryo at late stages of development. While this manuscript represents a quality scRNA-seq data set and will be a welcomed resource for researchers in Drosophila embryogenesis, the findings presented here do not provide mechanistic insights that would advance our understanding of organogenesis or embryonic development.

Comments for the author

In this revision, the authors make efforts to address some of the points raised by reviewers, however, they did not add any additional experimental validation to support any novel findings or hypotheses. Due to the lack of biological validation, some hypotheses are only minimally supported. While the sequencing analysis performed here is very thorough and will clearly be an important resource for the Drosophila community, the lack of novel biological insight suggests that this particular study is much better suited to be published under the ‘Techniques and Resources’ section, as noted by Reviewer 1, rather than an as a research article. For a research article, significantly more follow up experiments should be performed to test some of the hypotheses that were mentioned throughout the manuscript. Overall, this study is well suited as a resource article as written, but does not seem appropriate as a research article at this time.

© 2023. Published by The Company of Biologists under the terms of the Creative Commons Attribution License (https://creativecommons.org/licenses/by/4.0/).
